# Classification and molecular characteristics of *tet*(X)-carrying plasmids in *Acinetobacter* species

**DOI:** 10.3389/fmicb.2022.974432

**Published:** 2022-08-23

**Authors:** Chong Chen, Ping-Yu Huang, Chao-Yue Cui, Qian He, Jian Sun, Ya-Hong Liu, Jin-Lin Huang

**Affiliations:** ^1^Joint International Research Laboratory of Agriculture and Agri-Product Safety, Ministry of Education of China, Institutes of Agricultural Science and Technology Development, Yangzhou University, Yangzhou, China; ^2^Jiangsu Key Laboratory of Zoonosis, Jiangsu Co-innovation Center for Prevention and Control of Important Animal Infectious Diseases and Zoonoses, Yangzhou University, Yangzhou, China; ^3^Key Laboratory of Prevention and Control of Biological Hazard Factors (Animal Origin) for Agrifood Safety and Quality, Ministry of Agriculture of China, Yangzhou University, Yangzhou, China; ^4^National Risk Assessment Laboratory for Antimicrobial Resistance of Animal Original Bacteria, South China Agricultural University, Guangzhou, China; ^5^Guangdong Laboratory for Lingnan Modern Agriculture, South China Agricultural University, Guangzhou, China

**Keywords:** *Acinetobacter* species, mobile tigecycline resistance, *tet*(X), replicon typing, GR31 plasmid

## Abstract

The rapid dissemination of plasmid-mediated *tet*(X) genes in *Acinetobacter* species has compromised the clinical effectiveness of tigecycline, one of the last-resort antibiotics. However, the classification strategy and homology group of *tet*(X)-positive *Acinetobacter* spp. plasmids remain largely unknown. In this study, we classified them by genome-based replicon typing, followed by analyses of structural characteristics, transferability and *in vivo* effect. A total of 34 plasmids distributed in at least nine *Acinetobacter* species were collected, including three *tet*(X3)-positive plasmids and one *tet*(X6)-positive plasmid from our genome sequencing results. Among them, there were 28 plasmids carrying Rep_3 superfamily replicase genes and classified into six homology groups, consisting of GR31 (82.1%), GR26 (3.6%), GR41 (3.6%), GR59 (3.6%), and novel groups GR60 (3.6%) and GR61 (3.6%). Our *tet*(X3)-positive plasmids pYH16040-1, pYH16056-1, and pYH12068-1 belonged to the dominant GR31 group, whereas the *tet*(X6)-positive plasmid pYH12068-2 was unclassified. Structurally, all *tet*(X)-positive GR31 plasmids shared similar plasmid replication (*repB*), stability (*parA* and *parB*) and accessory modules [*tet*(X) and *sul2*], and 97.6% of plasmid-mediated *tet*(X) genes in *Acinetobacter* species were adjacent to IS*CR2*. Conjugation and susceptibility testing revealed pYH16040-1, pYH16056-1, and pYH12068-2, carrying plasmid transfer modules, were able to mediate the mobilization of multiple antibiotic resistance. Under the treatment of tigecycline, the mortality rate of *Galleria mellonella* infected by pYH16040-1-mediated *tet*(X3)-positive *Acinetobacter* spp. isolate significantly increased when compared with its plasmid-cured strain (*p* < 0.0001). The spread of such plasmids is of great clinical concern, more effects are needed and will facilitate the future analysis of *tet*(X)-positive *Acinetobacter* spp. plasmids.

## Introduction

Tigecycline, the first glycylcycline antibiotic, exhibits a broad spectrum of antibacterial activities against multidrug-resistant (MDR) Gram-negative and Gram-positive pathogens (Sader et al., 2019). However, the recent emergence and spread of novel tigecycline resistance mechanisms Tet(X3), Tet(X4), Tet(X5), Tet(X6), and other variants have compromised its clinical efficacy by enzymatic degradation (He et al., 2019; Sun et al., 2019; Wang et al., 2019; Chen et al., 2021). *Acinetobacter* species is a heterogeneous group of opportunistic pathogens and easily acquires antibiotic resistance genes (ARGs) (Wong et al., 2017). To date, the *tet*(X) genes have been reported in at least 10 different *Acinetobacter* species, including *Acinetobacter baumannii*, *Acinetobacter gandensis*, *Acinetobacter piscicola*, *Acinetobacter schindleri*, *Acinetobacter johnsonii*, *Acinetobacter indicus*, *Acinetobacter towneri*, *Acinetobacter lwoffii*, *Acinetobacter pseudolwoffii*, and *Acinetobacter variabilis* (Chen et al., 2020; Liu et al., 2020; Zheng et al., 2020; Cheng Y. et al., 2021; Li et al., 2021). Worrisomely, the plasmid-mediated *tet*(X3) and *tet*(X6) genes were detected with carbapenem resistance gene *bla*_NDM–1_ in *A. baumannii*, *A. indicus*, *A. schindleri*, and *A. lwoffii* isolates, posing a serious public health threat (Cui et al., 2020; He et al., 2020; Zheng et al., 2020).

The plasmid is a self-replicating component of *Acinetobacter* spp. genome and plays an important role in the horizontal transmission of ARGs, such as *bla*_NDM–1_ and *bla*_OXA–23_ (Wang and Sun, 2015; Silva et al., 2018). With the increasing number of complete *Acinetobacter* spp. plasmids deposited at the National Center for Biotechnology Information (NCBI), a series of plasmid classification schemes were developed based on replication initiator protein, mobilization protein and plasmid size (Bertini et al., 2010; Salto et al., 2018; Mindlin et al., 2020). As the latest research showed, there were a total of 59 homology groups identified by plasmid replicon typing (Li et al., 2022). However, there was a lack of systematic classification of *tet*(X)-positive *Acinetobacter* spp. plasmids since the first mobile plasmid-mediated *tet*(X3) gene in 2019, and their homology groups remained to be analyzed (He et al., 2019; Wang J. et al., 2020; Cheng Y. et al., 2021). Herein, we intend to explore the classification and homology group of complete *tet*(X)-carrying *Acinetobacter* spp. plasmids by genome-based replicon typing, followed by analyses of structural characteristics, transferability, and *in vivo* effect.

## Materials and methods

### Bacterial strains and plasmids

During an epidemiological surveillance between 2015 and 2018, we reported the prevalence of *tet*(X)-positive *Acinetobacter* spp. strains in China (Chen et al., 2020), of which seven isolates belonging to different sources were selected for next analyses in this study (Table 1). These included: *tet*(X3)-positive *Acinetobacter* spp. YH16040 and *tet*(X3)- and *tet*(X6)-positive *Acinetobacter* spp. YH12068 from pig; *tet*(X3)-positive *Acinetobacter* spp. YH16056 from soil; *tet*(X3)-positive *A. pseudolwoffii* YH18001 from human; *tet*(X4)-positive *A. indicus* Q22-2, Q85-2, and Q278-1 from migratory bird. In addition, a newly isolated *tet*(X6)-positive *A. baumannii* YC103 by CHROMagar™ *Acinetobacter* plates (CHROMagar, Paris, France) containing tigecycline (2 μg/mL) from duck in 2019 was also analyzed (Table 1). With the amino acid sequence of Tet(X3) as a template, the complete *tet*(X)-harboring *Acinetobacter* spp. plasmids deposited at the NCBI database were collected by tblastn (Supplementary Table 1; accessed 23 Mar 2022).

**TABLE 1:** Bacterial information of *tet*(X)-positive *Acinetobacter* spp. isolates by WGS.

Strains	Years	Provinces	Sources	*tet*(X)-carrying structures	
				Size (bp)	GenBank
*Acinetobacter* spp. YH16040	2016	Jiangxi	Pig	87,435	CP094542
*Acinetobacter* spp. YH16056	2016	Hunan	Soil	98,709	CP094546
*Acinetobacter* spp. YH12068	2017	Fujian	Pig	100,866	CP094556
				61,481	CP094557
*A. pseudolwoffii* YH18001	2017	Guangdong	Human	5,117	JALHBG010000013
*A. indicus* Q22-2	2017	Qinghai	Migratory bird	15,225	JALHBD010000003
*A. indicus* Q85-2	2017	Qinghai	Migratory bird	7,406	JALHBE010000004
*A. indicus* Q278-1	2017	Qinghai	Migratory bird	22,321	JALHBF010000002
*A. baumannii* YC103	2019	Jiangsu	Duck	4,021,945	CP054560

### Whole genome sequencing and assembly

Genomic DNA of eight *tet*(X)-positive *Acinetobacter* spp. isolates were sequenced by Oxford Nanopore (Nextomics, Wuhan, China), respectively. Combining the clean data with our previous Illumina HiSeq data (Chen et al., 2020) as well as that of *A. baumannii* YC103 (Novogene, Beijing, China), genome assembly was performed by Unicycler version 0.4.1 and corrected by Pilon version 1.12 (Walker et al., 2014; Wick et al., 2017).

### Bioinformatics analyses

All *tet*(X)-harboring plasmids were annotated by Rapid Annotation using Subsystem Technology (RAST) version 2.0 (Aziz et al., 2008). Plasmid-mediated ARGs were analyzed by ResFinder version 4.0 and a heatmap was then constructed by ImageGP (Bortolaia et al., 2020; Chen et al., 2022). Plasmid replication and transfer proteins were detected by the Conserved Domain Database (CDD) (Lu et al., 2020). A maximum-likelihood phylogenetic tree of replication initiator proteins was constructed by MEGA-X version 10.1.8 and visualized by Evolview version 3.0 (Kumar et al., 2018; Subramanian et al., 2019). Plasmid classification was performed according to the *A. baumannii* replicon typing scheme, with at least 80% nucleotide coverage and at least 75% nucleotide identity of replicase genes in the same group (Bertini et al., 2010; Castro-Jaimes et al., 2022; Li et al., 2022). Similarly, the bacterial host ranges of different plasmid groups were also evaluated by querying the NCBI database with representative replicase genes (accessed 24 May 2022). Sequence comparison of *tet*(X)-positive structures was conducted by Easyfig version 2.2.5 (Sullivan et al., 2011).

### Conjugation experiment

Transferability of plasmid-borne *tet*(X)-mediated tigecycline resistance was evaluated by filter mating with rifampin-resistant *Acinetobacter baylyi* ADP1 and *A. baumannii* ATCC 19606 (Chen et al., 2020). The putative transconjugants were selected on Luria-Bertani (LB) agar plates containing tigecycline (2 μg/mL) and rifampin (100 μg/mL), followed by *tet*(X) detection and PCR-based fingerprinting (Versalovic et al., 1991). In parallel, the recipient strains were selected with rifampin (100 μg/mL), and transfer efficiencies were calculated by colony counts of the transconjugant and recipient bacterial cells (Zhu et al., 2013).

### Plasmid curing

The *tet*(X3)-harboring GR31 plasmid pYH16040-1 was cured of *Acinetobacter* spp. YH16040 using sodium dodecyl sulfate (SDS) with adjustment (Chen et al., 2017). An overnight culture was diluted 100-fold in LB broth supplemented with 0.02% SDS and serially passaged at 38°C by shaking per 24 h. One week later, 100 μL dilution was streaked onto LB agar plate and 50 colonies were subcultured with or without tigecycline (4 μg/mL). The colony that did not grow in tigecycline was probably the plasmid-cured isogeneic strain (namely YH16040C) and confirmed by detecting *tet*(X3) gene (Chen et al., 2020) and replicase gene *repB* (repB-F, 5′-GCCCAATCGAATTATCAGCCA-3′; repB-R, 5′-TGGCAACAGAATCTAGGGCA-3′).

### Antimicrobial susceptibility testing

Minimum inhibitory concentrations (MICs) of all strains were determined by broth microdilution and interpreted according to the Clinical and Laboratory Standards Institute guideline M100-Ed28 (Clinical and Laboratory Standards Institute [CLSI], 2018). The tested antibiotics contained tetracycline, tigecycline, eravacycline, omadacycline, amikacin, gentamicin, ciprofloxacin, colistin, cefotaxime, meropenem, sulfamethoxazole-trimethoprim, and florfenicol. Particularly, the resistance breakpoints of tigecycline (≥8 μg/mL) and omadacycline (≥16 μg/mL) referred to the Food and Drug Administration (FDA) criteria for Enterobacteriaceae,^1^ whereas eravacycline was uninterpreted with no breakpoint. *Escherichia coli* ATCC 25922 served as a quality control strain.

### *Galleria mellonella* infection model

As previously described (Dong et al., 2017; Xu et al., 2021), the healthy larvae of *G. mellonella* (Huiyude, Tianjin, China) were randomly grouped (16 per group), and then infected with GR31 plasmid-mediated *tet*(X3)-positive *Acinetobacter* spp. YH16040 or its plasmid-cured strain YH16040C [*tet*(X3)-negative, 5 × 10^6^ colony-forming units] via the last left proleg. After incubation at 35°C for 2 h, the infected larvae were treated with tigecycline (2 μg/g) or phosphate-buffered saline (PBS) by injection into the last right proleg. Finally, the larvae were observed for survival rates per 24 h in the next 4 days. All *in vivo* experiments were performed in triplicate.

## Results and discussion

### Distribution of *tet*(X)-carrying plasmids in *Acinetobacter* species

WGS analyses of eight *tet*(X)-positive *Acinetobacter* spp. isolates successfully revealed four *tet*(X)-carrying plasmids. These included *tet*(X3)-positive plasmids pYH16040-1 (GenBank accession number: CP094542) from *Acinetobacter* spp. YH16040, pYH16056-1 (CP094546) from *Acinetobacter* spp. YH16056, and pYH12068-1 (CP094556) as well as a *tet*(X6)-positive plasmid pYH12068-2 (CP094557) from *Acinetobacter* spp. YH12068 (Table 1). Meanwhile, a *tet*(X6)-positive chromosome cYC103 (CP054560) from *A. baumannii* YC103 was also obtained (Table 1). For the remaining four *tet*(X)-positive strains *A. pseudolwoffii* YH18001, *A. indicus* Q278-1, *A. indicus* Q85-2, and *A. indicus* Q22-2 (Table 1), we failed to acquire the complete *tet*(X)-harboring plasmids or chromosomes despite repeated attempts. By querying Nanopore raw data of *A. pseudolwoffii* YH18001 (*n* ≥ 18, SRR18497244), *A. indicus* Q278-1 (*n* ≥ 4, SRR18497245), *A. indicus* Q85-2 (*n* ≥ 13, SRR18497246), and *A. indicus* Q22-2 (*n* ≥ 4, SRR18497247), a repeated structure consisting of multiple copies of *tet*(X) genes was detected, respectively, which may lead to the failure of complete sequence assemblies.

**FIGURE 1:**
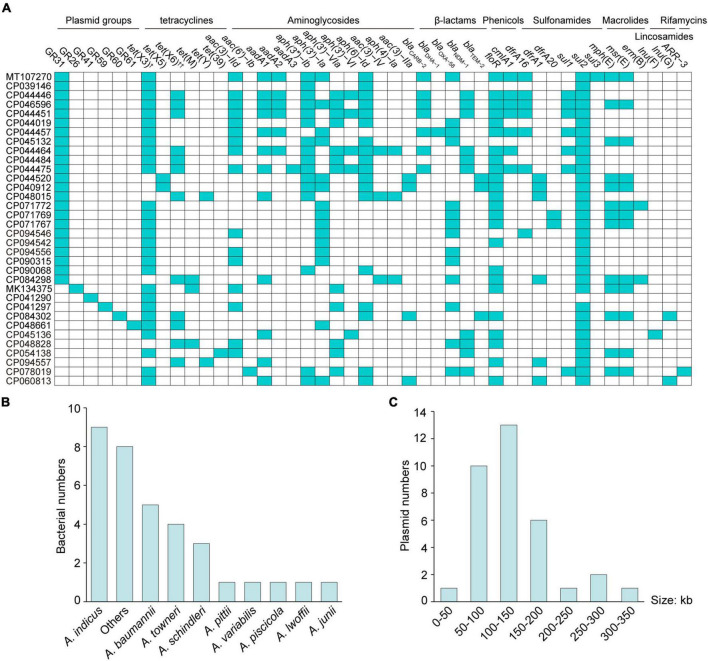
Collection of the complete *tet*(X)-positive plasmids from *Acinetobacter* spp. strains. The distribution of plasmid groups (cyan, **A**), plasmid-mediated ARGs (cyan, **A**), bacterial species **(B)** and plasmid sizes **(C)** is presented. ^†^One *tet*(X3) variant with a 99.7% amino acid identity (CP045132) with *tet*(X3) is included. ^††^Two *tet*(X6) variants (CP048828 and CP048661) with a 97.4% amino acid identity with *tet*(X6) are included.

Since the first report in 2019 (He et al., 2019), the number of complete *tet*(X)-positive *Acinetobacter* spp. plasmids deposited at the NCBI database has been growing (*n* = 34; Supplementary Table 1), including four plasmids mentioned above. There were three *tet*(X) subtypes located on plasmids (Figure 1A), namely *tet*(X3) (82.4%, 28/34), *tet*(X5) (5.9%, 2/34), and *tet*(X6) (35.3%, 12/34), of which *tet*(X3) usually coexisted with *tet*(X6) (23.5%, 8/34). Besides *tet*(X) genes, large amounts of plasmid-mediated genes conferring resistance to tetracyclines, aminoglycosides, β-lactams, phenicols, sulfonamides, macrolides, lincosamides, and rifamycins were present (Figure 1A). Worrisomely, the *tet*(X)-positive plasmids ranging from 42,489 to 332,451 bp were widely distributed in *A. indicus* (26.5%, 9/34), *A. baumannii* (14.7%, 5/34), *A. towneri* (11.8%, 4/34), *A. schindleri* (8.8%, 3/34), *A. variabilis* (2.9%, 1/34), *A. pseudolwoffii* (2.9%, 1/34), *A. piscicola* (2.9%, 1/34), *Acinetobacter junii* (2.9%, 1/34), *Acinetobacter pittii* (2.9%, 1/34), and unidentified *Acinetobacter* spp. strains (23.5%, 8/34; Figures 1B,C). Given the genetic and host diversity, more attention should be paid to the evolution of *tet*(X)-positive MDR *Acinetobacter* spp. plasmids.

### Classification of *tet*(X)-positive *Acinetobacter* spp. plasmids

**FIGURE 2:**
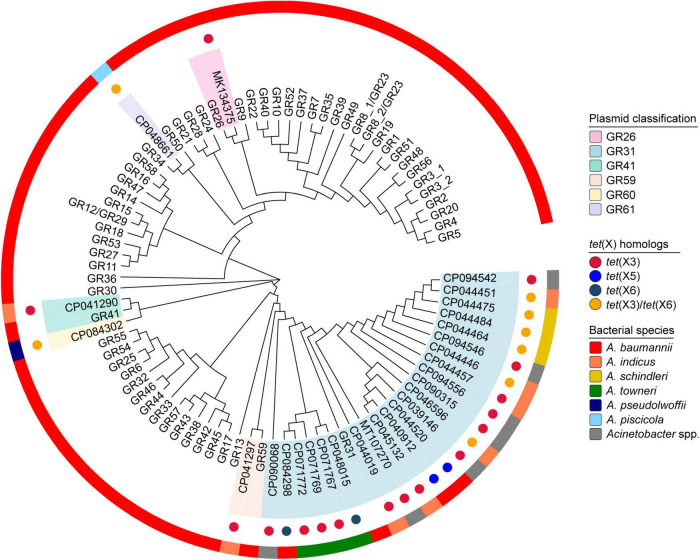
Maximum-likelihood phylogenetic analysis of *Acinetobacter* spp. plasmids based on replication initiator proteins. In parallel, the plasmid classification, *tet*(X) homologs and bacterial species are indicated from the inside out.

Replicon conserved domain analyses showed that 82.4% (28/34) of *tet*(X)-positive *Acinetobacter* spp. plasmids carried replicase genes and all of them belonged to a Rep_3 superfamily (pfam01051). Therefore, plasmid classification was conducted based on nucleotide sequence alignment with already classified replicase genes (Castro-Jaimes et al., 2022; Li et al., 2022). Among the *tet*(X)-positive Rep_3 superfamily plasmids, a total of six homology groups were successfully identified, such as the dominant GR31 (82.1%, 23/28), GR26 (3.6%, 1/28), GR41 (3.6%, 1/28), and GR59 (3.6%, 1/28; Figure 2). Especially, our *tet*(X3)-positive plasmids pYH16040-1 (CP094542), pYH16056-1 (CP094546), and pYH12068-1 (CP094556) fell within the same group GR31, which has been sporadically detected with *tet*(X) genes in *A. baumannii*, *A. indicus*, *A. schindleri*, *A. towneri*, and other *Acinetobacter* spp. strains (Figure 2 and Supplementary Table 1). However, our *tet*(X6)-positive plasmid pYH12068-2 (CP094557) was unclassified due to the lack of replicase genes. In contrast, the replicase genes of *tet*(X3)- and *tet*(X6)-carrying plasmids pXMC5X702-tetX-145k (CP084302) and pYH12207-2 (CP048661) have < 75% nucleotide identities with existing homology groups GR1-GR59, and therefore were defined as novel groups GR60 (3.6%, 1/28) and GR61 (3.6%, 1/28), respectively (Figure 2). We provided the updated replicase gene and protein sequences in Supplementary Files 1, 2, and hoped they will facilitate the future analyses of the increasing number of *tet*(X)-positive *Acinetobacter* spp. plasmids.

In order to evaluate the host range of plasmids belonging to GR26, GR31, GR41, GR59, GR60, and GR61, we conducted a blastn search against the NCBI database and confirmed they were mainly distributed in *Acinetobacter* spp. isolates (99.0%, 201/203). In essence, the GR31 plasmids have been detected in 13 validly named *Acinetobacter* species, including *A. baumannii* (18.3%, 15/82), *A. lwoffii* (17.1%, 14/82), *A. indicus* (12.2%, 10/82), *A. towneri* (8.5%, 7/82), *A. schindleri* (7.3%, 6/82), and others (Supplementary Figure 1). The plasmids belonging to GR26, GR41, GR59, GR60, and GR61 have also been identified in at least four *Acinetobacter* species, except two *E. coli* strains carrying GR41 plasmids (Supplementary Figure 1). Particularly, a few unassigned *Acinetobacter* species harbored GR26, GR31, GR59, GR60, and GR61 plasmids (Supplementary Figure 1), but the bacterial characteristics remained to be explored. All the results suggested a potential transmission risk of plasmid-mediated *tet*(X) genes especially in the genera *Acinetobacter*.

### Structural characteristics of *tet*(X)-carrying *Acinetobacter* spp. plasmids

**FIGURE 3:**
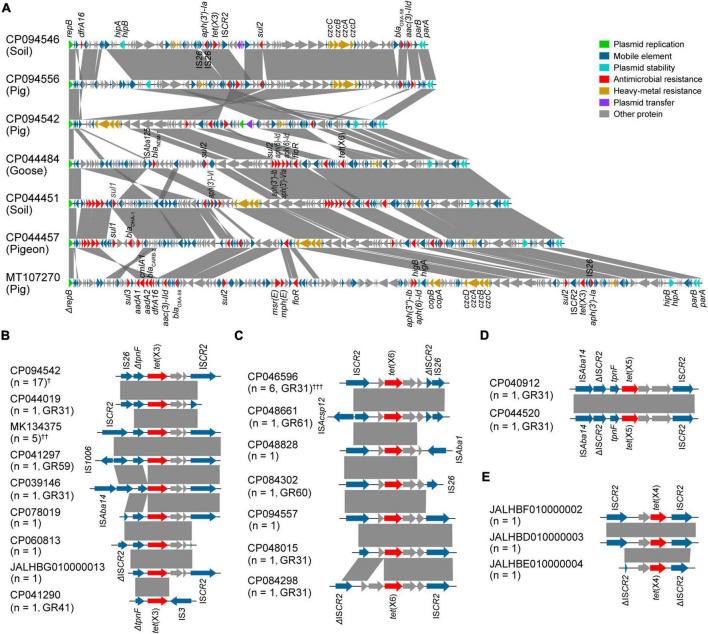
Characteristics of *tet*(X)-carrying structures in *Acinetobacter* species. The arrow represents the position and transcriptional direction of ORFs, while the Δ symbol indicates the gene is truncated. Regions of > 85% nucleotide sequence homology are marked by gray shading. **(A)** Linear sequence alignment of *tet*(X)-positive GR31 plasmids. The bacterial sources are listed in parenthesis. **(B–E)** Genetic environments of *tet*(X3) **(B)**, *tet*(X6) **(C)**, *tet*(X5) **(D)**, and *tet*(X4) **(E)** genes. The number of genetic environments and the plasmid homology groups are also given in parenthesis. ^†^These include *tet*(X3)-carrying GR31 (*n* = 15, e.g., CP094542), GR60 (*n* = 1) and GR61 plasmids (*n* = 1). ^††^These include *tet*(X3)-carrying GR26 (*n* = 1, MK134375), GR31 (*n* = 2), and unclassified plasmids (*n* = 2). ^†††^All of these belong to GR31 plasmids (*n* = 6, e.g., CP046596).

Predominantly, we compared the complete plasmid sequences of pYH16040-1, pYH16056-1, and pYH12068-1 with *tet*(X)-carrying GR31 plasmids available in the NCBI database. A total of 23 plasmids belonging to GR31 were analyzed and they exhibited GC contents ranging from 39.1 to 46.1%. According to our WGS results, pYH16040-1 was 87,435 bp and harbored 94 putative open reading frames (ORFs), whereas pYH16056-1 was 98,709 bp consisting of 107 ORFs and pYH12068-1 was 100,866 bp consisting of 115 ORFs. Although they originated from different sources, pYH16056-1 and pYH12068-1 showed an average 76.5% nucleotide coverage and 99.8% nucleotide identity to pYH16040-1, with the encoding sequence insertion, deletion and rearrangement (Figure 3A). Moreover, they shared high homologies (≥64.0% nucleotide coverage and ≥ 96.2% nucleotide identity) with MDR plasmids from the pig (MT107270), goose (CP044484), pigeon (CP044457), and soil (CP044451; Figure 3A). Archetypically, *Acinetobacter* spp. plasmids contained the backbone (namely plasmid replication, stability and transfer modules) and the accessory modules (Brovedan et al., 2020). Our results revealed all *tet*(X)-positive GR31 plasmids owned a *repB* gene involved in plasmid replication, and *parA* and *parB* genes responsible for plasmid stability. However, only 21.7% (5/23) of them harbored plasmid transfer modules, including conjugal transfer (*n* = 3) and mobilization genes (*n* = 2). For accessory modules, they mainly consisted of ARGs [e.g., *tet*(X) and *sul2*, 100% (23/23)] and heavy-metal resistance gene clusters [e.g., *czcA*-*czcD*, 78.3% (18/23)].

Genetic environments of all plasmid-mediated *tet*(X) genes in *Acinetobacter* species, including *tet*(X3) (*n* = 28), *tet*(X6) (*n* = 12), and *tet*(X5) (*n* = 2), were further analyzed (Figures 3B–D). The result showed 97.6% (41/42) of them were adjacent to IS*CR2*, which is able to transpose ARGs through a rolling-circle transposition process (Liu et al., 2022). For GR31 plasmids, the IS*CR2*-mediated transposition units were constantly truncated by IS*26* (*n* = 22) and IS*Aba14* (*n* = 3). Similarly, the truncation by IS*26* was found on GR60 (*n* = 2) and GR61 (*n* = 2) plasmids, one of which was also upstream truncated by IS*Acsp12*. Except the GR26 plasmid carrying a complete IS*CR2*-mediated transposition unit (*n* = 1), GR41 and GR59 plasmids were detected with truncation by IS*3* (*n* = 1) and IS*1006* (*n* = 1), respectively. In addition, our WGS results of *A. pseudolwoffii* YH18001 (JALHBG010000013), *A. indicus* Q278-1 (JALHBF010000002), *A. indicus* Q85-2 (JALHBE010000004), and *A. indicus* Q22-2 (JALHBD010000003) revealed the incomplete *tet*(X)-carrying structures were also related to IS*CR2* (Figures 3B,E). The close association between *tet*(X) variants and IS*CR2* as well as truncation by other insertion sequences indicated the frequent recombination events.

### Transferability of *Acinetobacter* spp. plasmid-mediated *tet*(X) genes

Conjugation experiments showed the GR31 plasmid-mediated *tet*(X3) genes were successfully transferred from *Acinetobacter* spp. YH16040 (pYH16040-1, CP094542) and YH16056 (pYH16056-1, CP094546) to the recipient *A. baylyi* ADP1 (transfer efficiency, approximately 10^–8^), but failed to *A. baumannii* ATCC 19606. In *Acinetobacter* spp. YH12068, the *tet*(X3)-carrying GR31 plasmid pYH12068-1 (CP094556) couldn’t be transferred, which may be explained by the lack of entire conjugative transfer regions (Figure 3A). Meanwhile, the *tet*(X6)-positive unclassified plasmid pYH12068-2 (CP094557) carrying plasmid transfer modules was transferred from *Acinetobacter* spp. YH12068 to *A. baylyi* ADP1 with a similar efficiency mentioned above. MICs of all transconjugants against tetracyclines increased by at least 32-fold when compared with *A. baylyi* ADP1, including tetracycline (≥64 μg/mL), tigecycline (≥4 μg/mL), the newly FDA-approved eravacycline (≥2 μg/mL) and omadacycline (≥8 μg/mL; Table 2). According to previous reports (Wang et al., 2019; Cui et al., 2020), the GR31 plasmid-mediated *tet*(X5) gene (CP040912) was also able to be transferred from human-derived *A. baumannii* to *A. baumannii* 5AB via electro-transformation, whereas the GR31 plasmid co-harboring *tet*(X3) and *tet*(X6) genes (CP044451) was from environmental *A. indicus* to *A. baylyi* ADP1 by natural transformation. The transferability of *tet*(X) genes between *Acinetobacter* species of human, animal and environment origins reminded us to consider the “One Health” approach to prevent mobile tigecycline resistance.

**TABLE 2:** MICs of *tet*(X)-positive *Acinetobacter* spp. isolates, transconjugants, and plasmid-cured strain.

Strains^†^	MICs (μg/mL)							
	TET	TGC	ERA	OMA	CIP	GEN	FFC	SXT
*Acinetobacter* spp. YH16040	128	32	>8	>16	64	0.25	128	>320
*Acinetobacter* spp. YH16040C	8	1	0.06	0.125	64	0.25	8	20
*A. baylyi* ADP1 + pYH16040-1	128	8	4	16	0.25	0.25	128	320
*Acinetobacter* spp. YH16056	128	16	8	>16	64	32	128	>320
*A.baylyi* ADP1 + pYH16056-1	64	8	4	16	0.25	16	4	320
*Acinetobacter* spp. YH12068	256	32	>8	> 16	4	16	256	>320
*A.baylyi ADP1* + pYH12068-2	64	4	2	8	0.25	0.25	128	320
*A. baylyi* ADP1	0.5	0.125	≤0.004	≤0.008	0.25	0.25	4	10

^†^ *All of them were susceptible to amikacin, cefotaxime, meropenem, and colistin. TET, tetracycline; TGC, tigecycline; ERA, eravacycline; OMA, omadacycline; CIP, ciprofloxacin; GEN, gentamicin; FFC, florfenicol; SXT, sulfamethoxazole-trimethoprim.*

At the same time, the sulfamethoxazole-trimethoprim resistance (320 μg/mL) was transferred with *tet*(X3)-mediated tigecycline resistance from *Acinetobacter* spp. YH16040, YH16056, and YH12068 to *A. baylyi* ADP1, as well as florfenicol resistance (128 μg/mL) from *Acinetobacter* spp. YH16040 and YH12068 and gentamicin resistance (16 μg/mL) from *Acinetobacter* spp. YH16056, which was consistent with the result of plasmid-mediated ARG mining (Table 2 and Figure 1A). Recently, our study has also reported the co-occurrence of mobile *tet*(X)-mediated tigecycline resistance and *bla*_NDM–1_-mediated carbapenem resistance between *Acinetobacter* species (Cui et al., 2020). With the co-transfer of multiple ARGs, the continuous application and selection pressures of tetracyclines, sulfonamides, aminoglycosides, β-lactams, and phenicols may promote the spread of *tet*(X) genes, as previously described for resistance determinants *mcr-1 and bla*_IMP–1_ (Wang Y. et al., 2020; Cheng Z. et al., 2021).

### *In vivo* effect of GR31 plasmid-mediated *tet*(X3) gene

To explore the potential effect of GR31 plasmid-mediated *tet*(X3) gene, a plasmid-cured strain YH16040C [*tet*(X3)-negative] was obtained by serially passaging of *tet*(X3)-positive *Acinetobacter* spp. YH16040. For tigecycline, a significant decrease in MIC was detected (1 μg/mL) when compared with the parental isolate YH16040 (32 μg/mL; Table 2). MICs of *Acinetobacter* spp. YH16040C against tetracycline (8 μg/mL), eravacycline (0.06 μg/mL), omadacycline (0.125 μg/mL), florfenicol (8 μg/mL) and sulfamethoxazole-trimethoprim (20 μg/mL) also exhibited at least 16-fold decrease. The result further confirmed the elimination of GR31 plasmid pYH16040-1 (CP094542) that co-harbored resistance genes *tet*(X3), *floR* and *sul2* (Table 2 and Figure 1A).

**FIGURE 4:**
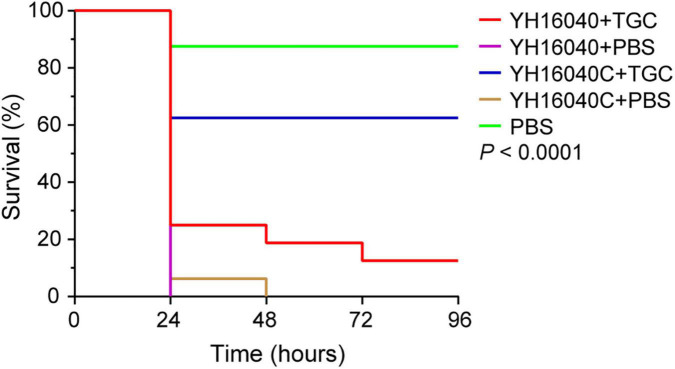
Survival curve of *G. mellonella* larvae under the treatment of tigecycline. The *P*-value is calculated by Log-rank (Mantel-Cox) test between GR31 plasmid-mediated *tet*(X3)-positive *Acinetobacter* spp. YH16040 and its plasmid-cured strain YH16040C [*tet*(X3)-negative] using GraphPad Prism version 8.3.0.

Subsequently, the *in vivo* effect of pYH16040-1-carrying *tet*(X3) gene was evaluated using a *G. mellonella* model. 96 h later of tigecycline treatment (2 μg/g), the larval mortality rate of *Acinetobacter* spp. YH16040C significantly decreased to 37.5% while *Acinetobacter* spp. YH16040 was 87.5% (*p* < 0.0001; Figure 4). The experiment was performed in triplicate with similar results, indicating the GR31 plasmid-mediated *tet*(X3) gene compromised the clinical effectiveness of tigecycline. Notably, the infection by *Acinetobacter* spp. YH16040 and *Acinetobacter* spp. YH16040C under the treatment of PBS resulted in a greater larval mortality rate (100%) than PBS only (12.5%) after 48 h (*p* < 0.0001), whereas no significant difference was observed between *Acinetobacter* spp. YH16040 and *Acinetobacter* spp. YH16040C (*p* = 0.32; Figure 4). The result suggested virulence genes outside pYH16040-1 remained to be studied. As previously reported (Jiang et al., 2021; Xu et al., 2021), *G. mellonella* models infected by plasmid-mediated *tet*(X6)-positive Enterobacteriaceae bacteria have also been constructed, and therefore *G. mellonella* was available for *in vivo* functional analyses of plasmid-mediated *tet*(X) genes.

## Conclusion

Taken together, the data presented in this study highlighted the diverse distribution of *tet*(X)-carrying MDR plasmids in *Acinetobacter* species. Our results classified the *tet*(X)-positive *Acinetobacter* spp. plasmids of Rep_3 superfamily into six homology groups, including two newly assigned GR60 and GR61. To the best of our knowledge, this study first revealed a dominant GR31 plasmid mediating the horizontal transfer of tigecycline resistance gene *tet*(X) across different *Acinetobacter* species and contributed to the failure of tigecycline treatment *in vivo*. Accordingly, more efforts are needed to monitor and prevent the plasmid-mediated *tet*(X)-positive *Acinetobacter* spp. strains.

## Data availability statement

The datasets presented in this study can be found in online repositories. The names of the repository/repositories and accession number(s) can be found in the article/Supplementary material.

## Author contributions

J-LH, Y-HL, and JS designed the study. CC, P-YH, C-YC, and QH performed the experiments. CC and P-YH analyzed the data. CC wrote the draft of the manuscript. All authors reviewed, revised, and approved the final report.

## Footnotes

1
https://www.fda.gov/
