# Supplementary material for: Classification and molecular characteristics of tet(X)-carrying plasmids in Acinetobacter species
Source: Front Microbiol. 2022 Aug 23;13:974432. doi: 10.3389/fmicb.2022.974432 (PMC9445619; doi:10.3389/fmicb.2022.974432)
Supplement: Supplementary file 1 [file Table_1.pdf]

Supplementary Table 1 | Information of the *tet*(X)-positive *Acinetobacter* spp. plasmids.

Plasmids	Accession numbers	Groups	Sizes	Bacterial species
p34AB	MK134375	GR26	277,864 bp	*Acinetobacter baumannii*
pYUSHP10-1	MT107270	GR31	174,032 bp	*Acinetobacter* spp.
p10FS3-1-3	CP039146	GR31	73,803 bp	*Acinetobacter* spp.
pCMG3-2-1	CP044446	GR31	120,957 bp	*Acinetobacter indicus*
pFS42-2-1	CP046596	GR31	140,938 bp	*Acinetobacter indicus*
pMMS9-2-1	CP044451	GR31	121,603 bp	*Acinetobacter indicus*
pAI01	CP044019	GR31	116,992 bp	*Acinetobacter indicus*
pB18-2	CP044457	GR31	136,195 bp	*Acinetobacter indicus*
p18TQ-X3	CP045132	GR31	93,847 bp	*Acinetobacter indicus*
pHZE23-1-1	CP044464	GR31	138,456 bp	*Acinetobacter schindleri*
pHZE30-1-1	CP044484	GR31	110,146 bp	*Acinetobacter schindleri*
pHZE33-1-1	CP044475	GR31	132,305 bp	*Acinetobacter schindleri*
p29FS20-1	CP044520	GR31	66,277 bp	*Acinetobacter baumannii*
pAB17H194-1	CP040912	GR31	88,002 bp	*Acinetobacter baumannii*
pAT205	CP048015	GR31	158,867 bp	*Acinetobacter towneri*
pGX7	CP071772	GR31	178,739 bp	*Acinetobacter towneri*
pGX5	CP071769	GR31	178,808 bp	*Acinetobacter towneri*
pGX3-1	CP071767	GR31	148,166 bp	*Acinetobacter towneri*
pYH16056-1	CP094546	GR31	98,709 bp	*Acinetobacter* spp.
pYH16040-1	CP094542	GR31	87,435 bp	*Acinetobacter* spp.
pYH12068-1	CP094556	GR31	100,866 bp	*Acinetobacter* spp.
pYUSHP17-1	CP090315	GR31	89,040 bp	*Acinetobacter* spp.
pYUSHP14-1	CP090068	GR31	187,536 bp	*Acinetobacter* spp.
pLHC22-2-tetX-162k	CP084298	GR31	162,264 bp	*Acinetobacter baumannii*
p94-2-tetX3	CP041290	GR41	42,489 bp	*Acinetobacter indicus*
p80-1-2-tetX3	CP041297	GR59	53,584 bp	*Acinetobacter indicus*
pXMC5X702-tetX-145k	CP084302	GR60	145,524 bp	*Acinetobacter lwoffii*
pYH12207-2	CP048661	GR61	148,815 bp	*Acinetobacter piscicola*
pXG01-X3	CP045136	/	103,629 bp	*Acinetobacter indicus*
pABF9692	CP048828	/	264,805 bp	*Acinetobacter baumannii*
pHNJXA13-1	CP054138	/	206,931 bp	*Acinetobacter pittii*
pXM9F202-2-tetX-90k	CP060813	/	90,430 bp	*Acinetobacter variabilis*
pYH12068-2	CP094557	/	61,481 bp	*Acinetobacter* spp.
pAJ_351-2	CP078019	/	332,451 bp	*Acinetobacter junii*
