# Supplementary material for: Classification and molecular characteristics of tet(X)-carrying plasmids in Acinetobacter species
Source: Front Microbiol. 2022 Aug 23;13:974432. doi: 10.3389/fmicb.2022.974432 (PMC9445619; doi:10.3389/fmicb.2022.974432)
Supplement: Supplementary file 2 [file Data_Sheet_1.DOCX]

>GR1_CU468231

TTGAAAAAAATATGTGTACTTATGAAGAAGGAACTTGTTGTCAAAGACAATGCACTAATA

AATGCCAGTTATAATTTAGACCTTTCAGAACAACGTCTAATATTGTTAGCAATCCTTGAA

GCTAGACAATCAAACACACCCAATGATAAAGATTTAACAATTCATGCTGAAAGCTATATC

AACCATTTTAACGTTCATAGAAATACAGCCTATAAAGTCCTTAAAGATGCATGTAAGAGT

CTATTTGATCGTAGATTCAGCTATCAAAAACTAACTCAGAAGGGCAACATTGAAAATGTA

ATAAGCCGATGGGTACAACGCATATCTTATGTTGAGAATGAAGCTCTTGTTCGTATTAAG

TTTTCTGATGATGTTGTACCGTTGATTACAAACTTAGAAAAACACTTCACCAGTTATGAA

TTAGAACAAGTCAGTAGTTTAACCAGTGTTTACGCTATACGCTTATATGAATTGCTTATT

GCATGGCGTAGTACTGGTAAAGTCATTTTGGTAGAGCTAGAAGAACTTAGATTAAAACTA

GGTATAGAATCCCATGAATATAAGAGAATGGGGCAATTTAAAGAAAAAGTTTTACACCTT

GCTATTGATCAAATAAACAAATACACCGATATAAAAGCAGAGTATGAACAACACAAACGT

GGCCGTTCGATTATTGGCTTTTCATTTAAGTTTAAACAGAAACAACAACCCCAAAAAGCA

GATTCCAAGCGAGCCCCTAACACCCCAGACTTCTTTGTCAAAATGACCGATGCACAACGC

CATCTATTCGCCAATAAAATGTCTGAGATGCCTGAAATGAGCAAATATTCACAAGGCACA

GAAAGCTATCAACAGTTTGCTATCCGTATCGCTGACATGCTTTTAGAGCCTGAAAAGTTT

AGAGAGCTTTATCCAATCTTAGAAAAAGCAGGGTTTAAAGGTTAA

>GR2_NC_010605

ATGAGAGATTTAGTTGTAAAGGACAATGCCTTAATCAACGCAAGCTATAACTTAGACTTA

GTAGAACAACGTTTAATTTTATTGGCTATTGTTGAAGCAAGGGAAAGTGGGAAAGGGATT

AATGCAAATGATCCATTAGAAGTTCATGCAGATAGTTATATCAATCAATTTGGTGTACAC

CGTAATACAGCTTATCAAGCCTTAAAAGATGCTTGTAAGGATTTATTCGCGCGTCAATTT

AGCTATCAAGAGAAAAAAGCTAATGGGAATATCCGAAATGTTATGAGTCGTTGGGTATCT

CAAATTGCTTATAACGACAATGAAGCAACTGTAGACTTAATATTTGCACCTGCTGTTGTT

CCCTTCATAACCCGACTGGAAGAACAATTTACTAAATATGAATTACAGCAAGTTAGTAGT

CTTAGTAGCGCTTATGCCATTCGCTTATATGAGCTTCTAATTCAGTGGCGAAGCACTGGT

AAAACCCCAACCATAGAACTACAAGAATTTAGAAAGAAGTTAGGCGTTCTTGATAATGAA

TATTTACGGATGGCTCATTTAAAAGAGCGTGTTTTAGAGCTTTCAATTAAACAAATAAAT

GAGCATACGGATATAACTGTAAAATATGAACAGCATAAAAGAGGACGTTCTATTTCAGGA

TTTTCTTTTACCTTTAAACAGAAGAAGAAGGATAGCCCATCAATAGAAAGAGATCCGAAC

ACTTTGGAGCTTTTTTCAAAGATGACCGATGCTCAACGGCATATGTTTGCAAATAAACTT

TCAGAACTCCCTGAAATGGGTCGCTATTCACAAGGAACAGAAAGCTACCAACAGTTTGCT

GTACGTATTGCTGAGATGCTACAAGATCCCGCTCAATTCAAAGAGCTATACCCATACCTA

AAAAAAGTGGGATACATGCCATCAAATAAAAAGGACACCGTAAATGGCTAA

>GR3_1_GU978997

ATGAAAACAGAACTAATAGTTAAAGATAATGCATTAATTAATGCCAGTTATAACCTTGAT

CTAGTCGAGCAACGGTTAATTCTTCTAGCTATCGTTGAAGCAAGGGAATCGGGTAAGGGA

ATAAATGCTAATGATCCATTAACAGTTCATGCTGAAAGCTACATCAATCAATTTGGTGTA

CATCGAAACACGGCTTATCAGGCATTAAAAGATGCTTGTGATGATCTATTTGCAAGACAA

TTCAGTTATCAAAGTCTTAGTGAAAAAGGCAACATTATTAATCACAAGTCAAGATGGGTG

AGTGAAGTCGCTTATATTGATAATGAAGCGGTTGTTAGACTTATTTTTGCTCCCGCTATC

GTGCCTTTAATTACAAGACTAGAAGAACAATTTACAAAGTATGAAATACAACAAATAAGC

AATTTAACAAGTGCTTATGCCGTTCGGTTATATGAGATATTGATTGCATGGCGTAGTACA

GGAAAAACGCCTCTTATAACTTTGTCTGATTTCAGACAAAAAATAGGTGTACTCGATACT

GAATACAAACGAATGTATGACTTTAAAAAATATGTCTTAGACATTGCATTAAAGCAAGTA

AATGAACATACCGACATTACTGTTAAAGTTGAACAGCATAAGACTGGTAGATCAATTACA

GGTTTTTCATTTAGCTTTAAACAAAAAAAATCAGTGACGAAGTCAGCTAAAAGTATAGGT

GTAAGCGAAGATATAACGATCACTTTAACAGATGCACAACGCTATTCATTTGCGAGTAAA

TTGTCAGAGCTTCCAGAAATGGGAAAACTTTCACAAGGCACCGAAAGCTATGAACAATTT

GCTGTACGGATTGCAGATATGCTAAAACAGCCGGAGAAATTAAAAGAACTTACTCCATTA

CTTCGAAAAGTTGGCTTTCAATAA

>GR3_2_GU978996

ATGAAAACAGAACTAATAGTTAAAGATAATGCCTTAATTAATGCCAGTTATAACCTTGAT

CTAGTGGAGCAACGGTTAATTCTTTTAGCGATCCTTGAAGCAAGGGAATCGGGTAAAGGA

ATAAATGCTAATGATCCTTTAACAGTTCATGCTGAAAGTTATATCAATCAATTTGGTGTT

CATCGAAATACGGCTTATCAAGCATTAAAAGATGCTTGTGATGATCTATTCGTAAGACAA

TTTAGTTATCAAAGCCTTAGTGAAAAAGGAAATGTTATTAATCACAAATCAAGATGGGTG

AGTGAGGTTGCTTATATTGATAACGAGGCTGTCGTTAGACTTATCTTTGCTCCCGCTATT

GTGCCTTTAATTACTAGACTAGAAGAACAATTTACAAAGTATGAAATACAACAAATAAGC

AATTTAACAAGTGCTTATGCCGTTCGTTTATATGAAATATTAATTGCATGGCGTAGTACC

GGAAAAACGCCTCTCATAACCCTGTACGACTTCAGACAAAAAATAGGTGTACTCGATACT

GAATACAAACGAATGTATGATTTTAAAAAATATGTCTTGGACATTGCATTAAAACAAGTC

AATGAACATACCGATATTACTGTCAAAGTTGAACAGCATAAGACGGGCAGATCAATTACT

GGCTTTTCATTTAGCTTTAAACAAAAAAAGTCAGCTACTCAGTCTGTCGGATCTAAAAGA

GATCCAAATACATTGGACCCTTTTTCAACAATGACAGATAAACAACGTCATCTATTCGCT

AGTAAACTCTCCGAGCTTCCTGAGATGAGTAAATATTCACAAGGTACGGAAAGCTATCAG

CAGTTTGCTGTACGTATCGCTGGCATGCTGCAAGATACAGAGCGATTTAGGGAAATTAAT

TCCTTTGCTCAAAAAAAGTAA

>GR4_GU978998

GTGCGGGATTTAGTAGTTAAAGATAATGCATTAATTAATGCGAGTTATAACTTAGATTTA

GTAGAACAGCGTTTAATCTTATTAGCTATTGTTGAAGCAAGAGATAGTGGTCGAGGCATT

AATGCCAATGATCCATTAGAAGTCCATGCTGAGAGCTATGTAAATCAATTTAATGTTGCA

AGACAAACAGCGTACCAAGCGTTAAAAGATGCTTGTAAAGATCTATTCGTACGCCAATTT

AGCTATCAAGAAATCAATAAGAGAGGAAATGTAGAAAATGTTTTAAGCCGCTGGGTCAGC

GAGATTAGATATATCGATGATGAAGCAACCGTGAAGTTAATATTTGCTCCTGCAATTGTC

CCACTTATTACACGTTTAGAAGAGCAATTTACTAAATATGAATTACAACAAATTAGTAAT

CTCAGCAGTGCGTATGCTGTGCGGTTATATGAATTGTTAATAGCTTGGCGCAGTACAGGC

CAAACTCCTATTATTGAACTAGCAGAGTTCAGGAAAAAAATAGGTGTTCTTGATGATGAA

TATACAAGAATGGGGAATTTCAAAGACCGAGTATTAAATTTGGCTATTGCTCAAATTAAT

GAACATACAGATATTAAAGTCCAATGTCAGCAACATAAAAAGGGACGTAATATTTCTGGC

TTTTCATTTACCTTTAAACAGAAAAAGGTCGTTATAGCTAACAATAAAAAGCAAACTACT

CTTGAGATTTTCTCAAAATTTACTGATGCACAGCGACATTTTTTTGCTAATAAGCTATCT

GAGCTTCCAGAAATGAATAAATATTCTCAAGGTACTGAAAGCTATTCGCAGTTCGCAGTT

CGAATTTCTGAAATGCTAAAAGATCTACAAAAATTTGAAGAACTACTGCCATATCTAGAG

AAAGTAGGCTTTAATGCAAAATAA

>GR5_GU978999

ATGCGAGATCTAGTTGTAAAAGATAATGCTTTAATTAACGCAAGCTATAACCTAGATCTA

GTTGAACAGCGACTTATTCTTTTAGCTATTGTTGAAGCAAGAGAAAGTGGTAAAGGCATT

AATGCAAACAACCCTTTAGAGGTGCATGCAGAGAGCTATATCAATCAATTCAATGTTGCA

AGACAGACTGCCTATCAAGCATTAAAGGATGCTTCAAAAGATTTATTTGCTAGACAATTT

AGCTATCAAGAGATGAATAAACGAGGAAACATCGAAAACGTACTAAGCCGATGGGTTAGT

GAGATTCGTTATATTGATGCTGAAGCGACTGTTAAGTTAATTTTTGCACCCGCTATTGTT

CCATTAATTACTAAACTCGAAGAACAGTTCACTAAGTATGAATTACAGCAAGTTAGTAAT

CTCAGTAGTGCTTATGCTGTACGCCTATATGAATTATTGATCGCATGGCGTAGCACTGGC

CAAACTCCTGTTATAGAGCTTGAAGAGTTTAGAAAAAAAATTGGTGTGCTTGATGATGAG

TACACAAGAATGGGGAACTTTAAAGACAGAGTCTTACATCTAGCTATAGATCAAGTTAAT

GAGTTTACAGATATCACTGTTAAATATGAGCAGCATAAAAAAGGACGTTCAATTTATGGC

TTTTCATTCTCATTCAAGCAAAAGAAAAACGTTAACAAACCAAATCTAGAAGCTAGAGAT

CAAAACACCTTAGATATTTTCACCAAGTTAACAGATGCCCAGCGTCATTTATTTGCTAAC

AAATTGTCAGAACTGCCTGAAATGAGTAAGTATTCTCAAGGCACCGAAAGCTATCCGCAA

TTTGCCGTACGAATTGCCGAAATGCTATTAGATGCTGAAAAATTTAAAGAACTATATCCA

TATCTAGTAAAGGTTGGCTTTCAAACAAAATAA

>GR6_CP002524

ATGGGCATACTTATGACTGTAAACTCTGTTAATTTAAATTCTAAAAAAGACTTTATAATC

AATAGATTATATGAAAACCTACCAAAAAAACCTTACTGTACCAGTGACTTCTTCGGATTA

AAGATTCGTGACAAGAAACAAGCTATACGTCACTCACATATACAAATTAATCATCCGAAT

TTTAAAAGATATATAGTTATTGATGCTGATTATCCAGGCGCAGCAACAGCTTGGCGGTAT

GATTTTGATGACAATATTCCAGTACCAAATTTAATTGTAGTTAATCCAGAAAATACTCAT

TGCCATTTTTACTATGAACTTGAAGCTCCAGTAAGTTTTACTGAAAGTTCCAGCAAGAGA

GCTCAAGAATTTTATAATTCAGTTTCTAAAAAGCTCACTGAAGTATTAAAAGGTGACAGC

AAGTACGTGGGACTAATAGCAAAGAATCCTGCACATGAAAAATGGATTGTAGAAGTACCA

CGATTAGAAAAGTATTCATTGCATGAACTTGTTGAACATTTAGAACTTAAGCCACATGAA

TACAGAAATATAAATTCAGAAAAAACCGGTATAGAGAAATTTGTAATTAACGGCCGTAAT

GATCATCTATTTAACGAAATACGGCATCAAGCATATATCGACATTAGAAGTTATAGAAGT

AAAACATTTGTTGAGTGGTTTGATCATGTAAAAAGCTTATTAATAAATGCAAATAAGAAT

TTTAGCGTTCCGCTACCATATTCAGAAGTTTGTGCTACAGCAAAATCAATCGCAAAATAT

TGTTGGAAAAAAGATAGCTACTGCTTTCAAGAGTTTTGTGAAAGGCAACATATTAAAGCA

AAGAAAGGTGGACGTGCAAAATCCGATAAATATGTTGAAATGAGGCGCACGGCTGCGCGT

TTATTGCGTTCTGGTAAAACTAAAACCTATATATCAGAATTACTTCAAGTATCGTACAGA

AGCGTTCTCCGTTGGCTTCAAGGCATAAAAGTACAAGCTGCAATAATGCATTTATCAGAA

TTAAAAAAGTTATGTGACAATGCCCAAAATCAGATATTAGCCTGCTTCATTGCTTCTCTA

ATCGTTATTATTTTAGATGAGCACATTTATGATTTTAATGAATCTGAAGAATTAAAAATT

ACCATCAAATTCAAATTAAAAGTTCCAATTCAATAA

>GR7_CU468233

ATGAAGAACAGTTTAGTTGTGAAAGATAATGCTTTAATCAATGCCAGTTATAACTTGGAA

TTAACAGAACAGCGTCTGATTATGCTTGCCATCATTAATGCCAGAGAATCAGGGCAGGGC

ATCACAGCCGATAGCAAGTTAGAAATTCATGCTAGTGATTATGCAAAGTTGTTTAATGTA

TCCATTGATGCTTCGTATAAAGCCCTTAAAGAAGCAGTGAATAACTTATTTAACCGTCAG

TTTAGCTATACAGCCGAATATAAAAGAACAGGGAAAACTGGTGTTGTACGTTCACGTTGG

GTTAGCCGTATTTTTTATGTTGATGATTTAGCATTACTAGAAATAACTTTTGCTCCTGAT

GTTGTCCCACTGGTAACACGCTTAGAAGAACACTTCACAAGCTACCAAGCCAAACAAGTC

GCACACTTAACAAGTAAGTACGCTACTAGGCTTTATGAGCTTCTTATAGCTTGGCGTGAA

GTCGGTAAAGTGCCACAAATAGAAATTAGTACTTTTAGAAATAGACTAGGACTTTTAGAA

AATGAGTACACAGCAATGAGTGACTTTAAAAAGCGTGTACTGGAGCCTTCTATTAAGCAG

ATCAATGAACATACAGATATTACCGTGACGTATGAACAGCATAAAAAAGGACGTTTAATT

TCAGGCTTTTCCTTTAAGTTAAAACAGAAGCAACAGCCAAAAATTGAAGTTAAGCGTGAT

CCCAATACACCTGACTTTTTTGTCAAAATGACTGATGCCCAACGTCATTTATTTGCCAAT

AAAATGTCTGAAATGCCTGATATGAGTAAATATTCGCAAGGAACAGAAAGCTACCAACAA

TTTGCTATTCGTATCGCTGATATGCTTTTAGAGCCTGAAAAATTTAGAGAGCTTTATCCT

TGCTTAGAAAAAGCTGGATTTCAGCCAGCTTAA

>GR8_1/GR23_GU979000

ATGAGTGAATTAATCGTAAAGGATAATGCTTTAATTCAGGCTAGCTATACTTTAGATACA

GTTGAACAAAGACTGATCTTATTAGCTATTGCTGAAGCTCGAGAAACAGGACATGGGATA

AATGAAAATAGCCTTCTACAAGTACATGCAAGTAGCTATATAAATACCTTTAATGTCGAG

AAACATACTGCCTATACCGTACTTCGAGATGCATCTAAAAGCTTATTTGATCGCTATGTC

ACATACCATGATATTAATCCTAAGACTGATAAAGACCGTAGCTTTCACTGCCGCTGGGTC

GACAAAATTGGATATGAACCTCAATCCGGAATCGTTTTCCTACGATTTACACAAGACATT

GTTCCACTCATAACTCGTCTGGAAGAAAATTTCACAAAATATGAACTGCAGCAGGTTTCA

AGGTTAACTAGCTCTTACGCTATTCGGTTATACGAGTTATTAATTCAATGGGGATCTCGA

GGGAAAACTCCAACTTTTGATTTACATGTCTTTAGAAACCGACTTGGTGTTGAAGATGGG

CAATATAAGACTATGTGCAATTTTAAACAATTTGTCTTAGATTTTGCTTTAAAACAAATT

AATCAATTTACAGACATCATAGCGAAATATGAACAGCATAAATCTGGACGAAAAATTACA

GGCTTTAGCTTTACCTTTAAATTTAAAAATAATAAAAACGTAAAAGAAAAATTAGTTGAA

AAAACTGAGTTTTATAAGCTCACTGAATCGCAACTAGACCTATTTGCAAAAAAGCTAGCG

CATTTACCCGAACTTGGACATTTAGCAGACGAAGGTATGTCTTATGAGGAATTTTATTCT

AAATTAAAAAGCATTTTAAAAGATCCAGAACAGCAAAAAAAATTAGTTCCCTATTTTGAA

AAAGCGGGATTAAATCCTAAATAA

>GR8_2/GR23_AY541809

ATGAGTGAATTAATCGTAAAGGATAATGCTCTAATTCAAGCAAGTTATACCTTAGATACA

GTTGAACAAAGACTGATCCTATTAGCCATTGCTGAAGCTCGAGAAACTGGACATGGGATA

ACTGAAAATAGTCTTTTAGAAGTACATGCAAGTAGTTATATAAATACTTTTAATGTCGAG

AAACATACCGCTTATACCGTACTCAGAGAGGCATCTAAAAGCTTATTTGATCGCTATGTC

ACATACCATGACATTAATCCTAAGACAGGTAAGGATCGTAGCTTTCATTGTCGTTGGGTC

GACAAAATTGGGTATGAATCTCAATCAGGAATTATTTTCCTACGATTCACCCAAGATATC

GTTCCACTCATAACTCGACTTGAAGAAAATTTCACTAAATATGAATTGCAGCAGGTTTCT

AGGTTAAGTAGCTCATATGCTATTCGGCTATACGAGCTATTAATTCAATGGAGATCTGCC

GGAAAAACGCCACTTTTTGATCTATCTATCTTTAGACAACAACTTGGTGTCAAACCTCAT

CAATACAAAACAATGAGTAACTTTAAAACATATGTTTTAGATTTTGCTCTTAAGCAGGTA

AATGAGTTAACCGATATAACAGCTAAATATGAGCAACATAAAAAAGGGCGTTCTATTTCA

GGTTTTTCATTCACTTTCAAACAGAAAAAAATGAGTAATCTGCCAATAAAAAATAAGCGT

GACCCAGACACTATAGATATTTTCTCAAAAATGACAGATGCTCAACGCCATCTGTTTTCC

CACAAACTGTCAGAACTTCCTGAAATGGGAAAGTATTCTCATGGTACAGAAAGCTATCCG

CAATTCGCTGTACGCATTGCAGAAATGCTTCAAAACCCAGAAAAGTTTAAAGAACTCTAT

CCTTATCTCCAAAAAGTAGGCTTCAAAGCTGCATAG

>GR9_CU468233

ATGGCAAATGATTTAGTCATTAAAAATAATGCGTTAATTGATGCAAGTTATACATTAAGC

CTAGTGGAGCAACGCTTAATTGGATTGGCTTTGGTTAAAGCGAATAATCAACATCAGGAA

ATTACTAGCGATACTGTGCTGACCATACATGCAGGGGAATATGCTCAACAATTCAATGTT

GATGGTTCGGTCGCTTACCGAGCGTTAAAGGAAGCATCAGAGCGTTTATTTTTACGCTAT

TTTTCTTACACGCTATATGGCTTAGATTTTGGTAAGGAGTACACACTTAAACGCCCAAAA

AAGTTAAAAGATGGTGATATCCCCACCATTATGAAGTCCCGTTGGGTACAAAAAGTAGGT

TATACAGAGTCAGAGGGGTTGTTACACTTTCAATTGACTAGTGACGTTGTACGATTGGTT

GCTAATTCAAAAGAGTATTTCACCAGTTACTACTTATCACAAACAACTGAATTTACTAGC

ACCTACGCAACTCGTTTATTTGAACTTCTAATGAAATGGAAAAATGTAGGACATATTCCA

TTTATTGAAATAGAACAATTACGAGGACAACTGGGTGTAGAGCCAAAGCAATATAAGATA

ATTTCTAACTTCAAGTTACGTGTCTTAGATGTGGCTGTAGAGCAAGTGAATCAATACTCT

GATTATAAAATTGAATATGAGCAACATAAGCAAGGAAGGACGATCACAGGCTTTTCATTT

AAATTTCAGCCCAAAGCAACGAAAACAAAGAAGATAGAATCAAGACGTGACCCAAACACA

CCCGACTTCTTTATAAAAATGACTGATGCACAACGCCATCTATTCGCCAATAAAATGTCT

GAAATGCCTGAAATGATTAAATATTCTCAAGGCACAGAAAGCTATCAACAGTTTACAATT

CGCATTGCTGATATGCTTTTACAACCTGAGAAATTTAGAGAGCTTTATCCAATCTTAGAA

AAAGCAGGGTTTAAAGGTTAA

>GR10_NC_010605

ATGAAAACTGAACTAGTAGTTAAAGATAATGCTCTTATTAATGCTTCTTATAATTTAGAG

CTTGCAGAACAAAGACTAATTTTACTTTCCATTGTAAAAGCTCGGGAAACAGGGCGAGGG

ATTACTTCTGACAGTCGTTTAGAGGTTCATGCTAGTGACTATATGAAGCAGTTTAACGTG

GAGAAAAGTGCAGCTTATGAAGTATTAAAGAGCGCATCAGAAAGTCTCTTTAATCGTTAC

TTTTCCTATAAGGAACAAAGACATGATGGAACGGAATTTGTGGTTAAATCACGTTGGGTC

AGTCGTGTAGCATATGCTCCCAATGTAGCTATATTGGAAGTAACTTTTGCACCAGATGTT

GTGCCATTAATTACTCGATTAGAGCAGCATTTCACTAGCTACCAACTAAAGCAAGTTTCT

CAACTTACAAGTAAATACGCTATTAGATTATATGAGATGCTGATTGCATGGCGTAATGTA

GGAAAATGTTCTTTTGAGCTCATCAATTTGAGAGATAGCTTAGGGATTGCATCTGATGAG

TATAAGCAAATGGGGCATTTCAAAAGTCGTGTTCTAGACGCATCAATTGCTCAAATTAAT

GAGTACACAGACATCAAAGTGACTTATGAACAACAGAAGAATGGTCGAACTATTAGTGGA

TTCACCTTCAAGTTAAAGCCTAAACAAGTACAACAAGAAATTACCATCCTAGATACAAAA

GCATCGTTAATTCCTTCTGATCTGACTCCAAACCAACGTGTTACTTTTGCTAGTAAATTA

TCAAAACTCCAAGAATTAGGAGGAAAAGCAGAACCTGGTGAGGAAGTAGAGGCATTTGCA

AAACGAATTGAGTTATGGCTAGAGGATGAAAAAAAATTGAAGATGCTTACTCCATTTCTC

TATCAAGTAGGATTTAAAAAAGCTAAACCAAAGAAAGTATCTCAGTAG

>GR11_NC_010401

ATGAATAAAGAAAATAGTTATGATAAATCTTATCCAGTAACAACAATGGCTATTCAAAAC

AAAGTTACTGAATGCTTTAAAAGCATGTCTGTAGATGAAAAAAGAATTTTAATTATGGCT

TCTCCGATTGCTAGAAATGTCGATGCAAGTGAACAAGATCAAATCTTAATATCTGCTCAA

CAATTTGCTGATGACTGTGGTATCAAAGTCAATTCTGCTTATAAACAAATTGAAAATGCG

TCAAAAAAACTAGTAGATCGGTCCTTTTCATACGTTAATGATAGGGGGAAAAAGGTCTAC

TCTAACTGGGTAATTGATGCTACTTATGAAGATGCAGGGATATCTTTAAGGTTTACATCT

ATTGTTTTGGTGATGTTGAAAATTTTAGATAAATACAATCCATACACTCGTTATAAAAAA

GATGTAGTTCTAAAATTAAAAAAAGACTACTCAATAGACTTTTACCATTTAGCGAAAAAA

AATCAGGCAAAAAATGGCTTTGAATTAACGCTAGATGAAATGTTTACAGAGTTTGGTTTA

CCAGAATCTTATAGAGATTTGAGGAACTTAAAACGAAGAGTTTTGAAGAGCTCATTAGAT

GAAATTAATGAATTTACCGATGTAACAGTTGACTATAGTCCAGTTAAAAAAGGACGTTCT

GTTGTCGGCTTTAAGTTCACTGTGAAAGAAAAATCTAAGCCAAAATTAATAGCTCCTGAG

CGAGATCCAAAAACAATAGATATGTTCTGCAACCTGTCTGATGCTCAAATTAACAAGTAC

AGTGCTATTTTATCTAAACTTTCTGAGCTATCAGACCTAAGTAACTTCCAGGACTATCCT

AGTTTTGCCCTTTGGATTAGTGGCATCTTACGAGATCCGAAAAGTGTAAGAGAGGAAACA

GCAAAGCGGATTTTTAAAGCTCTTCATAGCAAGACGGATTTTAAACCATGA

>GR12/GR29_CU468232

TTGAATAAAAATCACGTTGTAAAATCAAATCAAGTAATAGAAGCCTCATATCAATTAAGT

GCCGTAGAGCAACGAATTGTCTTAGCTGCCATTTCACGGATTCCAAAAAATCAGCCTATT

ACAGATGATGAGCTATATCCTGTTAGTATCAATGAGTTAAGGCAATTAGGGGTACATGAA

AAAACTGCATACAGAGATTTAAAGGAAGGTATTAATAGACTCTATGAACGATCTATTAAC

CTCAGTATTGATGACAAGTCTATAAAAATGCGATGGGTACAAGAAGTTCAATTTCTAGAT

AGTCAAAGTGTCATTGGTATTCGTTTTTCAAAACCAATTCTGCCTTTCATATCTAATCTA

AGTAGAGAATTCACCAAATATGCCCTGTCAGACATCGCTGGAATAAATAGTGGGTACGGT

ATTCGTATTTATGAGCTACTTGTTCAATATCGACAAATAGGTAAGCGTGAAATTTCAGTT

GAGAATTTGCGGACAATGCTTGAGCTTGGTAAAAAGTACCCTTTATTCGCAGATTTCAAG

AAACGAGTAATTGATACTGCTATAGACCAAATAAACGAATGCAGCCCTTTAAATGTCACT

TATGAACAGAAAAAGACTGGTCGTAAAGTTACTAGTATTATTTTTTCATTTAAAGAAAAG

ACTAAGAGTATTAGTCATCAGAATACAGATGTACCTAAAGAATTTTATAAACTAACAGAT

GCTCAAATTAATATGTTTGGAAATCAACTTTCTCGTTTACATGAACTATCACATCTAGCA

CAGCAAGGTGAAAGCTATGATGATTTAGCTATAACAATTAAAGATATGTTGAGAGATCCA

AAACAGCAAAAACAGTTTTTACCTTATCTAAAAAACTTAGGCTTTAAACTTTGA

>GR13_NC_010404

ATGAGCAATCACAACGAACCAGAAGAATTAGAACATCTACCTTATTGTATTGGTAACATT

CGACATAATGGAGTAGTCAGCACTAGTAACCGTCTGATTAGACCAATTGAATTATCATCT

AATGAGTATAAAGCTCTTCTTTATGCAATGGCTGTTGCGAATTATGGTGAGAAGAATAAT

CAAGATCGTGAAATTACAGAGCAAACTTATATTTATTTGCATAAAGATGATTTAGGCGAA

TTATTAGGATTAGATAAAAAGAATTCCATTAATGTTGCTATTGATCGTATTTATAAAGAA

TTATCATCACGAGTAGCTCATTTTGTAATTGAAGAGCCCGTGGATGATAATAAGCGTAAA

GTTAAAAAAGTACACTCAGTAGTACCAATTATTCGTGAACTCCGTTGGGAAGATGATGCT

AAAAATGCTTTGCAGATACGTTTCACCAGTGAAGTACTTCCGTATTTTACTCGTTTAGCA

AATGGAAATTTTACGACCTATCAATTGAAAGATTTATTTGCTTTGGACTCTGTAACCAGT

ATGAGTTTGTATTCATACTTTATCAAAAATGAATTTAAGTATGCTAATCAAGATTCTTAT

GAGGTTGAACTTTCACTTGAGAATATCAAAGCATTAATTGATATTGGGGAGAAAAAATAC

GATCGTTGGGTTGATTTTAGACGATACGTTTTAGACAAGATTATTGAAGAAATTAATGAA

AGGACTAGTCTTCAATTAGAGTACGATACCATCAAGAAAGGTCGACCTATTGTCGGAGTT

CGATTTAAAATTCTTAATAGGCATGCCACTGAAGTTGTTGTATCTAATACTAATGATAAG

ACTCAAATCTATTTAGATGTCAATTTTGATGATAACGCGCTTGTTAAAGAATTAGGTGCG

AAGTTCGATATGACTGTAAGGTCTTGGTACATCTATGCAAACGACCCAAATTATAAACAG

TTTAGTAAGTGGTTTAAGACAGAAGGGTGCTTAACTGAATCTCAAGCAAATGTGATAGTT

AATGATATTATGTTCCAAATGGATTTTGCTGTAGCTGGCAGTTCAATGAGTGAATTTAAG

AAAGAAATGAAATATAAATTAAAAAATAATCCAAATTTTGTAAAAGAAAATAGAAAACGA

TTGAATGAAATCTTTGGAAAAGACGTTATATGA

>GR14_NC_010403

TTGCAGAATTTAGATAAGAAAAAACCCCTGTTATCGGACAGTTTGGCGACCGGTGATAAC

AAGGGTTTTGCATCTCCCAAAGGAGATCAACATAGGGATAGAATAACACGTTTTGGCATT

TTGAAACATAGATCGAAGCAACAAGAAAACTATTTATTTTCGTTAGCTAAGATTAAAGAA

AATTATCATGCCGATGTAAAAAACGATGAATCTATTCGCGCCATGAAAACTGCCCAAAAA

TTAAATGGGTGCGGTAATTTTCTTCTATTCAAAAATTTTTACACCATTAATCAAATTAAA

CTCGCCAAGTTCCAAGCTTGTAGTGAGCATTTGTTATGTCCGTTTTGTGCTGGTATTAGA

GCTTCTAAGGCAATTCAAAAATACTCTGAGCGTGTTGATCAAGTCTTATCTGAAAATCCT

CGTTTAAAGCCCGTTATGATCACGTTTACGGTTAAAAATGGGGTAGACCTAGGGGAACGG

TTCACCCATCTTATAAAATCGTTTAGAACGCTTATAGAGCGTCGTAGGGACTATATTAAA

AAAGGGCGTGGCTTTAATGAATTTTGCAAAATTAATGGTGCGATGTATTCATATGAGAAT

ACTTACAATGAAAAAACTAATGAATGGCATCCTCATATTCATATGTTTGCACTTTTGGAT

GATTGGATAGATCAGGATGAATTGTCTCAATATTGGCAATCCATTACTGGGGACTCTATG

GTCGTTGATATTCGTAGAGCCAAAAAACAAAAAGACTTAGGCTATTCAGGTGCTGCTGCT

GAAGTCTGTAAATATGCTCTCAAATTTGGTGATCTTTCTGTAGAAAAGACTTGGGAAGCT

TTCAAAGTTTTGAAAGGTAAGCGATTAAGTGGGGCTTTTGGATCTCTTTGGGGCGTGAAA

ATTCCTGAATCATTGATAGATGATCTTCCAGACGATTCTGATTTACCTTATTTAGAAATG

ATTTATAAGTTCGTCTTTTCTAAGAAGTCTTATTACGATTTACAACTTACTCGTCATGTC

GAACCTACAGGTAAGGACGACGCCGACGAGCTTCGAGGAGAAGAAGGACGCAACCTGTTG

GTGAGCATGGACGGGCGAGGAGCGAGCGACGCTGGGAGGGCCCGCACTGGCGCGCTAGCC

CCGCAGCACGGACGAAAAAAACAACACTGGCAAATTCCACCAGTTACTCGTGTTCGGGTT

CGGAAGCGAATCCGAAGATGGGACGGATATTTATGTGTCTTACATTTATAG

>GR15_CU468233

TTGGAAATAAAAATGATGAGTCCATCTAAAAAAGAACTAGTAGTTAAATCAAATCAAGTT

ATTGAGGCTTCATATCAACTCAGCTCAACAGAACAGCGTATTGTGTTAGCAGCTATTAGT

AAAATTAGTCGGGCTGAAGACATTACAGATGATGAAATTTATCGCGTAACTATTGATGAC

TTAAAAAAACTTGGGGTTCATGAAAAAACAGCTTATAGAGATCTAAAAGATGGTGTAAAC

CGCTTATATGACAGATCAATTAATTTAGCTATTAATGATGAATCAATCAAAATGAGATGG

ATTCAATCTATACGATTTTTAGAGAGTAAAAGCGTTGTAGGAATTAGGTTTTCTAAAGAA

ATTTTACCTTTCATTTCTAATCTAAGTCGTGAATTCACTAAGTATTCATTATCTGACATT

GCTGGTATGAGTAGTGCCTATGCTATTCGTATTTATGAGTTATTAAGCCAATACCGTTCA

ATAGGAAAACGGGAGATTCCCATCGAATCTCTAAGAAGTATGTTGGAGCTAGGAAAAAGA

TATCCATTATCTGCTGATTTAAAAAGATGGGTTATTGATACAGCTGTAGATCAAATCAAT

GAACATAGCCCTTTAAATGTATCTTACCAGCAAATAAAAACAGGCCGAAAAGTCACACAC

ATTCAATTCACGTTTAAAGAAAAATCAAAAAATATTGAGCATAAATCTGAACAGAATGAT

TTTTATAAATTGACTGATTCACAAATTAATATGTTTGGTAATCAGCTTTCACGCTTACAT

GAAGTATCTCACCTAGCACAACAAGGTGAAAGTTATGACGACTTAGCTATCAAAATTAAA

GATATGCTGCGAGATCGAATACAACAAAAACAACTTATTCCTCATCTTAAAAATTTAGGT

TTTAAGGCATAA

>GR16_L77992

ATGTCGATGAATATATTTTATGGGGAAAATCCGTCCTCAGCAAGCTTAAAGAGCCTCGAA

AATAGCCAGTCCCTTGGTATAAAGACGAAATCTCATGTAATGCTTACGCCACAAGGCTTT

CAGCGTGTGCATGATTATCTATTACAAGACCAATCTAGAAAACTTCTTCCTAAAGAACGT

GTATCTAAATGTAGACGTCTCCGGATCGATAAAACTAAGACTAGAACTGTTATGTATAAC

GAGCATCGAGAGAAGGCTCATTATGGCAATGTTCAAATCTGCGGTTCTATTTGGTCATGT

CCTGTTTGTGCCAAGCAAATCACACAGAAAAGACGTAATGAATTAGGTAAGGGCATAGAG

TCGTGGAAAACGGTTCATAATGGCTCTGTATATCTCCTTACGCTTACTTTTAGCCATTCA

CCTGACCAATCCCTCAAAAGTAATTTAGAGGGCCTTAAACGCGCAATGAAGCGTTTTTAT

GAGACAACTCGAGTTCAGGCTATTTTTAAAAAACTATCTGTTTTTCACAAAATAAAAGGC

CTAGAAGTTACATACGGTCAGAATGGTTGGCATCCCCATCATCATGTACTTCTTTTAGCT

GAACATCATGATTTACGTTTTAAAGATTACACTTCTGAATTAACGGAGTTATGGATTAAA

GCCTGTATTAAATCAGGATTAAATGCTCCATCGATGCGCCACGGTTTAGATCTTCGAAAT

GGCTCTTATGCTGACCAATATGTGTCTAAGTGGGGCCTTGAAGATGAACTTTCGAAAGGG

CATGTGAAAAAAGGTCGCAATGGTGGTTTTACCCCTTTTGATCTTTTAAATTTTTCTATT

GAAGATAATGAAATTTATGGAAAAAAACCTTCTAAACTTTTCCAAGAATTTGCCATTTCT

ATGAAGGGTGCTCGCCAACTAGTTTGGTCTCGAGGTCTTAAAAAACTTTTAGGTATTGAA

GAAAAAAGTGACGAGGAACTTGCAGTAGAAACTGACAAAGCTTCCATTACTTTGAATCGT

GTTGAAGATCTCGTTTTTGAACTTTTATGTCGTTATCAATTGCGTCATCAATATCTTGAA

GCAATTAAACATGATTATGAGACTGGCTCTTTTGGTTCTGGATTAGCAGATCAACTTATT

GAGCAAGTAGTGAATTATGAAATTAAACAAATGCAGCAGGTATTTTCGTGA

>GR17_CP000522

ATGGCTAAGTTATCGCTAAGCGAAGTATCCAAACGCTTTAACGTCAGTCGTTCTACATTA

TATAGAGCAATAAAGGAAGGCCGCATATCCCGAAATGCAGATGGTTATTTTGACGTTGCG

GAAGTTATTCGATGCTTTGGAGAACCCAGCAAAAAGCATGAGCAAAACCAAGAAATAGAT

AAGCCTAAAGATGATACTGATCTACGTCAGCTAGTTGATTTCATGAGAAAGGAAATTGAT

TCATATAAGGATCGAGAAAAACGTTATTTAGATCAGATTGACCGCTTCCAATTATTATTA

GGGCATAAAGAGTCCGAAGAGAAAATGTCTCATGACACATCAGTGAGACAAACCAATGAC

ACACCTTGTGACAACAATCATGAAACACATAAAGATACTGTAAATCAAGAATTTTATTCT

ACTGAATCACCTCATGATACACCACTAACACATCTCAATGAAGCACCACAAAACATATCA

AAGACACATCATGAGACAAAGAAAAAACGTGGCTTATTTGGCCGTGTTTTAAATGCCGTA

TTTGATAATGATTGA

>GR18_CU468232

ATGTTATTCTCATATTTGATAACAAGGAATACAAAAATGAGTAAGCTCTTAGTAGTCAAG

GCAAATAATATTATTGAAGCTAGTTATCAACTGTCTTTGAATGAACAGCGCTTAATCTTG

GCGGCTATAGCCTGTATACCTAAAGGTGAAGAAGTGACAGATAACACAGGTTATTGCGTC

ACAAGGGAATCATTTATTGAATTAGGTGTTAACCCCAAAACAGCAAGCAGGGAAATCAGA

GAAGCATGCGATCGGCTTTTTAATCGGGTTATCACAATAACAACTGAGGCAGGGACGTTT

AAAACTCGATGGGTTCAAGACATCATGAAATACAATAGTGATTGGGCTTTGGCTAATCCT

GAATTTATCCAAGAGGTGGCAGGGAGCGATCCTTATGCAGAAGATTATATTTTGGCTGCA

ATAAGATTTAGTAAGTCTGTATTACCTTTTATTAGTAACTTATCGTCTAACTTCACACAA

TATTTTCTTCAAGACATAGCAGGAGTAAGTAGCGGGTATAGCGTGCGATTTTATGAATTA

ATGATGCAGTTCAAGAGTACAGGTTATCGAAAAATAAGGCTTGATGACTTGCGTAATATG

CTTGATCTGAATAATAAGTATCCACTGACGGCAGACTTAAAAAGATGGGTAATAGATACC

GCAATTGATGAACTTAATGAGAAATCCCCCATTACAATCAAATATAAACTATTAAAAACA

GGCCGTAAGTTCACACACATAGAATTAAAATTTAAGCAAAAGTTATCACCCAAAAAAATA

GAATCTCAACGAGATCAAAAAACTATAGATATGTTCAGCAACTTATCAGACAGTCAAATT

AAGACCTACAGTTCAGTATTATCTAAGGTTCATAGCATTTCAGACTTAGCCGACAATAAA

GATTATTCAGCGTTTGCTATATGGATTGCCAATGTTCTACGTGACCCTACATCCGTCCGA

GAGGAAACAGCAAAGCGGATTTTTAAAACATTGCGGACTGAAACAGATTTTAAAGGTTAA

>GR19_GQ861437

ATGCTCATTGTTAAAGATAATGCACTGATTAACGCTAGCTATAATCTAGAACTTGTTGAA

CAAAGATTGATTTTGTTGGCAATTATTGAAGCTAGGCAAAACGGAAAAGGAATTAACACA

AATGATCATTTAATAGTTCACGCTAGTACATACATAGAACATTTTAATGTTGAAAAACAT

AGTGCTTACATGAGTCTTAAAGAAGCATGTAAGAATCTATTTGCAAGACAATTCAGTTAT

GAAGAAATAAATCCAAATGGTAGTAGTACTCAATATACAAGTCGTTGGGTATCAAAAATT

GGATATACAAAAAAAGAAGGAACTGTTCATATTATTTTCGCACCAGATGTTGTACCCCTT

ATAACCAGGCTCGAGAAGCATTTTACAAGTTACGAACTAGAACAGGTTGCACAACTTCAA

AGTAAGTACGCAACTCGGTTATATGAAATTTTAATTGCATGGCGCAGCACTGGGAAAGTG

CCTGAAATTTCACTATCTGAATTTAGGGCAAAATTAGGAGTTTCTGATTCTGAATATAAA

ATAATTTCAAATTTTAAATTGCGAGTACTAGATGTTGCTGTTAGCCAAATCAATAAATAT

ACCGATATTACTGTCACGTATGAACAGCATAAAAAAGGACGAACAATTATAGGCTTTTCA

TTCAGATTTAAACAAAAGCAACTAGCAAAGAAAATTGAATCTAAGCGAGATCTAAACACA

CCCGACTTTTTTATAAAAATGACTGATGCACAACGTCATCTATTTGCGAATAAAATGTCT

GAAATGCCTGAAATGTCTAGCTATTCACAGGGTACTGAAAGCTT

>GR20_NC_012813

ATGAGAGAATTAGTTGTAAAAGACAATGCCTTAATTAATGCAAGCTATAACTTAGATTTA

GTAGAACAACGTTTAATTTTATTGGCCATTGTTGAGGCAAGAGAAAGCGGGAAAGGTATT

AATGCTAATGATCCCCTTGAAGTACATGCAGAAGGCTATATCAATCAATTTGGCGTACAT

CGCAATACGGCTTATCAAGCATTAAAAGATGCCTGTAATGATTTATTTGCAAGACAATTT

AGCTATCAAAAAATAAATGAACGAGGGAATATTGAGAACTATAGATCCCGTTGGGTTAGT

GAAATTGGATATGTAGATAATGAAGCAGTGGTTAAACTTATCTTTGCCCCAGCCATAGTT

CCCTTAATTACACGCTTAGAAGAGCATTTCACTAAATACGAATTGCAGCAAGTTAGTAAT

CTCAGCAGTGCTTATGCTGTTCGCTTATATGAATTATTAATTGCTTGGAGAAGTACTGGC

TCTACTCCTATTATAGAGGTAAGTGATTTCCGTCAAAGAATTGGCGTACTCGATACAGAG

TACAAGCGTATGGAACGCTTTAAAACTAGTGTACTTGAGCTTGCTATTAAACAAATTAAC

GAACATACAGATATCACTGTGAAGTATGAGCAACACAAAAGAGGTCGATCAATTTCAGGA

TTCTCTTTTACTTTTAAACAGAAAAAGAAGGACAACCCACCGATAGAAAGAGATCCGAAT

ACGTTAGATCTCTTTACAAAGATGACTGATGCACAACGCCATCTGTTTGCAAATAAACTT

TCTGAACTTCCTGAAATGGGTCGTTATTCCCAGGGAACTGAAAGCTATCCGCAATTTGCT

ATTCGTATTGCTGAGATGCTGCAAGACCCTGACCGAATAAAAGAACTATACCCATACCTA

AAAAAAGTGGGATATATGCCATCAAATAAAAAGGACACCGTAAATGGCTAA

>GR21_KY984046

ATGGGTGAGTTAGTAGTTAAGTCAAATGATTTAATTAATGCATCTTATAATCTAGGAGTT

GTCGAACAAAGATTACTCTTATTATGTATTATTGCTGCACGGAAAAAAGACAGAGTCTTG

TCTCCTTCTGATATCTTTTATATACATGCATCTGAATATATTGAACAATTTGATGTAGAT

CGTAGTGTAGCTTATCGAGCATTAGCCGAAGGCATAAAAGGAATTTATGACTCTGAAATT

AAATTAACATCCAAGAATTCCAGAAAAAAAATTAATATTAGATGGTGCTGGAAAGCTGAA

TATGATGAAGATCATGCAACTGTCGGAGTTGCATTTACAGACGATGTTATTCCTCTTATT

TCTGCACTTGAACAACGTTTTACATCATATGATATAGATCAGATCGCTAAATTAACAAGT

AAGTATGCAATTAGGCTATACGAATTAGTTATAGCATGGCGTTCAATTAATAAAACCCCT

GTATTCGAGTTAGAAGACTTTAGAAATAAGCTTGGCTTAGGTGTTAGTGAATACAAAACA

ATGAGTAATTTTAATAGTAATGTTTTAAATATTGCTATTCAACAAATCAATAAATTTACA

GACATAAAAATTAAAGTTCACAAACATAAAAAAGGTGTACGAATTGTTGGCTTTTCGTTT

GAGCTAACACAAAGAAAAATGAAAAATCAAAATAGTACTAAGGATACGTTTTACAGATTA

ACAGACTCACAAATCAACATGTTTGGTAATCAGTTATCTAGGCTACATGAAGTAGCCCAT

TTAGCAGTTGAAGGTGAAAGCTACGAGATATTAGCAGCAAAAATCAAAGAAATGCTGAGA

GATCCAATACAACAAAAGCAGTTTTTACCCCACCTACAAAACCTTGGCTTTAAAGCTTGA

>GR22_KY984047

GTGAAAAATGACCTCGTAGTGAAGGATAATGCGTTAATCAATGCTAGCTATAATTTAGAC

ACAACAGAACAACGATTGATTCTCTTGGCGATTGTGCAGGCAAGAGAAGTAAGCAAGGAT

GTGGACGCTAATAGCACGTTAGAGGTTCATGCTCATCATTACATGAAGCAATTCAATGTT

GATAAGCATGCAGCCTATGAGGGGCTTAAAAACGCTGCTAGCAACCTATTTGAGCGCAAA

TTCAGTTACAAGGGTATCCATGAGGGAACTCAACAGGAAAAGATCGTCAAATCACGGTGG

GTTTCCAAAATAGCCTATGTTGATTCTGCAGGTATCGTAGAACTCACTTTTGCACCTGAT

GTCATTCCATTGATTACGCAGCTTGAGAAGTCATTCACTGCTTACGAACTTAAGCAAATT

AGCTCTCTTACAAGTAAGTATGCTATTCGCCTTTACGAGTTACTCATCCAATGGCGTAGT

GTTGGAAAGACACCTATGTTTGATATTGATGACTTTCGATTCAAGCTTGGGTTAGCTGAA

GGTGAATATGCAAAGATGGCGAACTTTAAGGTTCGAGTACTAGATATAGCATTAAACCAG

ATCAATGAACTGACTGATATCACAGCTTCTTACGAACAACACAAAGTAGGGCGTACGATT

AGTGGTTTCTCTTTTTCATTCAAACCTAAGCAACATGTGGATGCAATTACGCATAATAAA

CCTAAGAAATTGACAGATAAACAGATTCAATTCTTTGCCAACAAGCTAGCTCACCATGAT

CCATTTGCAAGCAAAAAAGCTGCAGTAGGGGAAAGCTATGCTGATCTAGAGAAAAGGCTT

CTGATTGAACTACAAGATGTCGAGTTTGTCAGAAAGTATGCTAGTGTTCTCAAAGAGTTA

GGTCTCGAGGTATGA

>GR24_AFCZ02000003

ATGGCAGAACTTATTAGGAATTCAGATGTTTATAAAGCGAATGCATTGATTAATGCAAGC

TACGCTTTGGACACTGCTGAGCAAAGAATAATTCTACTCGCCATTTTAGTTTCCAGAAAC

AAGAATGCAGATCTGACTGCCGAAACGATTATCGAGATTCCGGCTTCCTTATATGCCCAA

AAATTTAATACAACAGTGAGCGCGGCATATAAAACACTGAAGGAAGCCGAAGATACCTTA

TTTGAAAGACGTTTTTCTTACACCACAATGCGAAATGGCAAGATTGAGGTGGTTCGGTCA

CGTTGGGTATCACGAGTTTCATACGTTAAGGATGATGCATTATTAACGATCACCCTAGCT

CCTGATGTGATTCCTCTAGTAACCAAGCTAGAAGGAACCTTTACCAAATATGCCATCGAC

AATTTACGCGATGTGACCAGTAAATATGGCATCCGTCTGTATGAGTTGGTCGCTAGTTGG

AAAAATTCGGATATACGTAAAACTCCTGTTTATGACTTTGAGGACTTCCGTGCCAAGATG

GGCCTTCTTCCTCATGAGTATAGAGACAAGAAAAATCCCGAAAGTACGGATATGACCAAC

TTCAATAAACGTGTATTGAAGCCGGCAATAGATCAGATTAATAGTTTTACTGACCTGTTT

ATTACTGAAAAGAAAATCAAGACAGGACGTAATATCACGGGCATTTATTTTGAAGTAAGT

TTAAAAACCGATAACTTCATTGAAGGCGAAGCGAAAGAAATCCATGACAGCAAGCCTTCT

TCCGATTCAGCTAAAAAGACAGGCACTCCTTTAGAAAACATTAGGCTTCCAAAAGTATCG

ACCCAAGAGTTTTTGGGTAGTGATCTCAGCGAAGAGGACCTTAACAAAGAGAACCCATTA

AAAGAATTTATCGTTGAATCTGGCGTTTATAAGTCTGCTATTGAAAAGCCAGTAGAAGAA

AAGGATGAATTTGAATTAAATGGCATAAAACGACTGTATGAGGCACTATTAAAGCTGGAT

GAGGGCGTAACCAAGGAATACGTCCGCGAATATGCCCAGATTAAAGGCGTAACTCTACAA

CACGCATTAATTGAACTTTATAACTCTAAAAGACCGGCTTAA

>GR25_NC_017848

ATGATAACTAGCAATATCTTTGCTACTGATAATAAATCTATATTAATCAGTAGGTTGTAT

GAAAATCTACCACGAAAACCATATTGTACCAATGATTTCTTTGGGTTACGGATTCGTGAA

AAAAAATCTGCAATATCACACTCGCACATACAATTTAATCACCCAAGCTTTAAACGCTAC

ATCGTAATTGATGCTGATTATGCTGGGGCTGCTACAGCTTGGCGCTATGAATTCGCAGAA

AATATACCTGTTCCAAATCTAATAGTCACAAATCCTGAAAACAGTCACTGCCATTTTTAT

TATGAATTAAGTGCTCCAGTAAGTTTTACAGACTCATCGAGTAAAAAAGCACAAGAATTT

TATAATGCTGTTAGTAAAAAGCTTACTGAAGTACTAAGAGGGGATACAAATTACACAGGA

CTGATAGCAAAAAACCCTGCTCATGAAAAGTGGATTGTTGAAGCCCCTAGAATTGAAACA

TACAGCCTACATGAGCTTGTAGAGCATTTAGAGCTACATCCTCACGAATATCGGTCAATA

CCAGGTCAAAATAGCAAGCAAGAGCAAGTACAATGTATAAATGGCCGCAATGACCATCTT

TTTCATACAGTAAGAGTTAAAGCCTATGTTGATGTTAGAGACTTCAGATCAAAAACCTAT

CCACAATGGGAAGAACATGTAAGACAATTGCTGGTAGATCATAATTTAGAGTTAAATAAC

CCATTACCTTATTCAGAAATTAAAGCAACGGCCAAGTCGATAGCAAAATATTGTTGGAAA

AAAGACGGCTATTGCTATCAAGAGTTTTGTGATCGCCAAATATCTAAAGCAAAGAAAGGT

GGTCAAGCTAAAGCAGATAAATATATAGAATTAAGAAAAAAAGCAGTGGCTTTGCTTAGA

AAGGGGAAAAATAAAAGATTAATAGCTCAACTTCTAAAAGTTTCCTACAGATCAGTTCTA

CGTTGGCTTTATAACGTCAAACTAGCGGCGGCCATAATGCATCTTAGAGACTTAAAGAAC

ATGTGTGACAATGCCCAAAATCAGATATTAGCCGCTTTTGTTGCCAGCTTAGCTGTCTTA

TTCCTTGATGAGTTTATTTATGACTTTACTCAAGATGATATTTTAACAATTAACTTAACT

TTTAATATTAAGATGCTTATTTAG

>GR26_CP015365

ATGGCCGACTTAGTCGTAAAATCAAATAAACTTGTTCAAGCCTTACAGACACTTACTTTA

AGTGAAACTCGCCTATTACAGCTCGCTATTGTCGATGCTCGTGAAACAGGCCAAGGATTA

TCAGCAGAAGAACCATTAGAACTAAATGCTAGTAGATATGCTACAGCCTTTAATGTTTCA

CCTGATGCTGCTTATTTAGCTTTAGTTGAGGCAGAAGATTCTTTATTTAAAAGACAATTT

ACTATTACCAATGAAGATGGAACTTTAACAAAAAGTCGTTGGATTCAAGATGCTAATTAT

CGAAAAGGTGAAGGTCGCATACTAGTAACATTAACTCGTGTTGTAATTGAACATGTCACA

AAAATAGATGGCTTTGAACAATATTTTACTAGTTACCATCTTAAAAAAACTTCCGATTTT

AAAAGCGTTTATGCTGTACGACTTTATGAGTTGTTAATGCAATGGAAGTCTGTAGGCAAA

ACACCTATATATGAGTTGAATAAATTTCGTAGCCAATTGGGTATAGGAGTTAATGAATAT

GATCGTATGGAGGCTTTTAAAAGACGTGTTTTAGATATTGCTATTAAACAAATTAATGAG

CTTTCTGATATTACCGTTAAATATGAGCAACATAAAAAAGGTCGTGCTATTTCAGGTTTT

TCATTCGCCTTCAAACAAAAGAAAACGAATCAACCAATAGCAGATAAGCGAGATCCAAAC

ACGCTAGATCTTTTTTCAAAAATGACGGATGCTCAACGTCATATGTTTGCTAATAAGCTT

TCAGAATTACCTGAAATGGGGAAATATTCTCAAGGTACAGAAAGCTACCAACAGTTTGCT

GTACGTATTGCTGAGATGCTACAAGACCCTGAAAAAATCAAAGAACTATCCCCATACCTA

AAAAAAGTGGGATACATGCCATCAAATAAAAAGGACACCGTAAATGGCTAA

>GR27_AYFH01000057

ATGGACGAAAATAAAAAAACGTATCCACCTTCTTGGGTTGTGATGCAAAATAATATTCAA

GAATGTTTTAAAAGTATGAATATTGATGAAAAGCGTATATTGATACTTGCTAGTCCAATT

GCTAGAACTACTCAAGCAACAGAAAAAGATCCTATTATGATTACCGCTGAAAAATTCGCT

GAAGAGTGTGGAATTAAAACGCATTCAGCATATACACAGTTAGAAGTAGCAAGTAGAAAT

CTGATTAAGCGTAGTTTTTCTTATAACAATGAAAGAGGGAAACGTGTTCTGTCTAATTGG

GTAATTGATTGCATTTATGAAGATGGAGGTATTGCAATTCGATTCCCTGAGATCGTTTTA

TTGATGCTTACAGAATTTGATAAATTAAATCCATATACAAAGTACAAAAAAGACATCGTA

CTTAGTCTAAAAAAAGATTACTCATTCGATTTTTATCACTTAGCTAAAAAACATCAGGCT

ATGGGAAAATTTGAAATGTCTTTAGAAAGAATAAGAACTGAATTTGGCTTACCTGAGTCT

TATCACGATCTAAGTAATCTAAAAAAACGTGTTATCAATCCATCACTAGATGAGATCACA

GCTAATACAGATATTGCCCTAACTTATGAAAACGTTAAAAAAGGGCGTTCTGTGGTTGGC

TTTAAGTTTACTGTCAGAGAGAAGCCCAAGCCTAGATTAATAGCATCAGAGCGAGAGCAA

GAAACACTAGATATATTCCGCAGTCTGTCTGATGGTCAAATCAATACCTACAGCTCTATT

TTGTCAAAAGTAGGTAGTATTAGTGATCTAGCTGGAGCAAAAGACTATCAAGCATTTGCA

ATTTGGATTGCTAATATTTTACGAGATCCAAAATCAGTCAGAGAGGAAACCGCAAAACGT

ATATTTAAAGCCTTGCGGACTGAAACAGATTTTAAAGGTTAA

>GR28_AFDB02000003

ATGACAAATACCAATAAGTTAGTTGTAAAGGATAATGCTCTTATTGATGCCTCTTTCAAT

TTATCCCTAATAGAGCAACGGATTATGCTTCTAGCAATAGTTGAAGCTAGAGAATCAAAC

AGTCTTTCCCCAGATACCCCTATAGAAGTATCTGTGAGCGACTATATTCATCAATTTAAA

GTGGATAGCAATAATGCTTATGCCCTACTTAAAGATGCTTCTAAAACCTTAAAGCGAAGA

GAGTTTAGCTATTTAGATAGATATAAGGGCATAGAAGCACTTTCAACAGCTAATTGGGTT

AATAAAGTGACCTATGTTGATAAGAGCGGTTTGATTGTTTTATATCTTAGTCATGAAGTA

ATTAGCTTAATTAGTAAATTAAGTGAACAATTCACAAAATACTATATCGAGCAAGTTTCT

GAATTTAAAAGCAAATATAGTATTCGATTATACGAACTTATTATTAAGTGGTTAAGCGTT

GCTAAGACTGAAAAATACAGTATCAATGATTTAAGATCAAAGCTTGGACTTGGTGTTGAA

GAGTATTCAACTATGACCAATTTCAAGTCTAATGTATTAGACAAAGCAATTAATGAAATC

AATAAACATACTGATATAATTGTAGATTATCAGCAATTTAAGAAAGGAAGAGTGATTACT

GATATCCAGTTCTTTATTAAGTCTAAAGCTCGACCATCTAAACAAACTAACACTACCAAG

CAATCTTTTTATCAAATGAATGATGCACAAATTAACTTATTTGGAAATCAGTTATCTCGT

CTACATGAACTGTCTCATTTAGCAAATCAAGGTGAAAGCTATGACGAGTTAGCGATTAAA

ATTAAAGATATGCTCAGAGATCCAATACAGCAAAAACAACTTCTTCCACATCTTAAAAAT

CTAGGTTTTAAGGCTTAA

>GR30_AYOI01000002

ATGTCCTCTATTGTTAAAAGCAATCCAAAAGTTAAGAAACACAATAACTTAACACAGGCT

CATTTTTTTAATGTATCAGTCATCGCTTATAGACTTATATTATTAGCCGGTACGGACAAG

TTCTTAGAAAACATGCTAAAGTCTGGCGAGAATACTTACATTCGCATTACAGCGCATGAT

TATCACAATTTATATGGCTCAAGTTCGGACATGTCGGGTTCGTATAAAGCAATTAAAGAT

GCGCCCGATGATTTACTTAATGCCAAGTTGAAATACAAAAGACTTAAAACAGAATCAGAC

CCCGGCCGTTGGGTAGGTGGTATTAACTGGGTACAAGACGCTCGTTATAACGACGAGTTA

AAATGTGTTGAAATTCTATTTTCTACAACAGTACTTCCTTTGCTGGCGAATGTTCGCAAA

AGTTTTACTTATTACAACCTACGACATATTGGTCGGTTGTCATCCATGCATTCGATTAGG

ATGTATGAACTAATGATGATGTGGCGCAAAAGCGGCAAAACGCCAGACTTGACAGTTAGC

TATATGAAAAACTTCTTAGGCGTGCCAGACAATGAATATTCCGACCCAAAAGAGTTAAAG

TTTTTCACAGCTCAAGTAATTAAAAAGTCCGTCAAAGAAGTCACAAGTAAAACTAATATT

GAAATGGACTTTGAAGTTGTAAGAGGGGAAAAGAGAGCAACTATCGGCTATTCTTTTAGT

CATAAATTGAAAGCGCTGCCTGAAGGTGAACAGCCTGAGCAAGAAGAACTTGAGGACGAT

AACGAAGGCGGCGGAGATCCAAGCAAATTGCTACCGAACAATGACGACGACCCAGAGTTG

CCATTTTAA

>GR31_CP012956

ATGACAGAATCTGAACCATTGCAAAAAGAATCGAATTATATTCCTTACTGTATAGGCAAC

ATTCGCCATAATGGTGTTGTCAGTACCAGTAACCGTTTAATTCGCCCAATCGAGTTATCA

GCCAATGAATACAAAGCGCTTCTATATGCAATGGCAGTTGCGAATTATAGTGAGAAAAAT

AGGGTAAATGGTGAAATCACCGAACAAACCTATATTTATTTGTATAAGGATGATTTGGCA

GATTTACTAGGATTGAATAAACGTAATTCAATTAATGTTGCAATAGACCGCATCTATAAA

GAACTGTCATCCCGGGTGGCTCATTTTATTATTGAGGAGCCTGCTGATGATGGTAAGAGA

AAAACTAAAAAAGTACATTCTGTCGTTCCCATTATCCGTGAGTTAAGATGGGAAGATGAC

TCTAAAAATGCGATTCAGATTCGTTTTACCAATGAGGTATTGCCGTATTTCACTCAACTG

GCGGGGGGGAATTTTACCACCTATCAGCTAAAACATCTTTTTGCCTTGGATTCTGTTGCC

AGTATGAGTCTTTATACGTATTTCATTAAAAACGAATTCAAGTACGCCAATCAAAAGAGT

TATGAAGTGCCATTACTTTTGGAAAACTTGAAAGCAGTGATTGATATTAATGAAACCAAA

TATGATCGCTGGGTAGATTTTAGACGCTATGTATTAGACAAAATAGTGGCAGAAATTAAT

GAAAATACAGACCTACAATTAGAATATGAAACCGTTAAGAAAGGCCGTCCAATTGTGGGT

GTGAATTTTAAACTGCATCATCGGATTGCTGATAAAGCACCGGATGAAATTGCAGTAATT

GAAAAAATCTATCTTGATGTTCCATTTGAAGACAATGCATTCGTAAAAGAATTGGGGGCG

AAATTCGATACGAATATACGTTCTTGGTATATTTTTAATAATCATGAAAATTATCAGCAA

TTCAAGAAGTGGTTTAAAAAAGTAGGATGTCTCACTGACTCTCAAGCAAATATCGTTATT

AATGACACTTTATTCCAAATGGATTTTGCTGAAATTGGTATGGGTCTGAATGACTTTAAA

CGAAATATGAAGCATAAACTTAAAAATAATCCGGAATTCGTTCAAAGTATTCGTGAAAGA

CTGAATGATATTTTTGGGAAAGAGCTTATTTAG

>GR32_CP013925

ATGTTCGGAATGACAAATAATAACTTAGCACTTGCAAGCAATGATTCATTAACCATTGTT

CTCAAACGTTTTTATAATAATCTTCCCGATAAGCCGTATCACTCAAACGGATTTGATGTT

GAGGGGCTGAAAATTAATCGGAAAATTGAAGCAATCAAGAAAAAATACATCCAGTTTAAT

CATCCAAAATGGAAAAAATACATTTTAATCGACATAGATCGCCCTGGTGCGGTCACAGAT

TGGCTCTATGAATCTCCGCATCTACCGGCACCAAACCTGATAATAGAAAATAGAAAAAAT

GGACATGCTCATTTCGTTTATGAATTAATAGATGCTGTGAGTTTTACAGAACGCAGCTCA

TTAAAAGCAAAGAACTACTACAATGCGGTAGAAAAGGCATTAACAAGCGAATTAGGCGGT

GATGAGCGATATAATGGGGTTGTAGGAAAAAACCCGTATTCTGAGGAATGGCGTACTTCA

ACATACAGAACCGAAGCTTATCACTTAAAAGACCTGGCATCTAAACTTGAGCTAACAACA

ATGGGTTTAACGCCCATAGAAATGCCACAAAAAGCTCAGAATGATGAATGTGCCATTAAT

GGCCGGAATGATGAAGTGTTTCATTCAGTACGTCACCTAGCCTACAAGGATATAAGGGAT

TTCAAGAATAATGCTGACCTGTTATTTAATCATTGGTTTGATCATGTACTGAAACTGGTT

CAGGAAAAAAACTCATTTTTTATAAATCCGATGGACTATAAAGAATGCACACATATTGCT

AAGTCCATAAGTGAGTATTGTTGGCGCAATCATGAAGAGTGCTATAAACAATTTGTGGAA

AGACAACGTAATAAAGGTTCAAAGGGTGGAACCAGCAGATCCGCAAAATACGAAGAAGCA

AGACGCATGACAAAACAGTTGTTTCGACAAGGTGTTTCCCTCAAGCAAATTGCTGAAAAG

CTCAATATCTCGTATAGAACGGCCGTTAGGTACACCAAAGGGCTATTGCGGATTAAATTA

TTAAGTTTCAATGATATAAATAATTTGCGTAAATCCGCCCTGGCGGACAAAAAAGCGCGA

AGCGAAGCGAAGTGCATAAAGTCCGAGCGAAGCGAGAGCATTAATAATGCTTTTAATTGG

TGTGACAGTAGCCAAAATCAGGTATTAGCCGCTAGGCACACCCACCCAGCCCCTTTCGGA

GTATTTTTTAAAAAGCTCTTAAGCTTCACTTTTAAAAATTTAAAATTTGAACGGTTAAAG

GGGGGAGCAATATTTTATTATTATGGGAAAATACCTTAA

>GR33_CP012005

GTGAAGAAGCCTAAGCATGACCTGACCCACGTCCGACATGATCCCGCGCACTGTTTGGCA

CCTGGCCTGTTCCGCAGCCTCAAGCGTGGCGATCGCAAACGCTGCAAGCTGGACGTGACC

TACACCTTTGGCGAGGACGAATCCATGCGTTTCGTCGGATTCGAACCTCTCGGGGCCGAT

GATATGCGTCTTTTGCAAGGCATCGTGGCCCTTGGCGGCCCGAACGGCATCTTGCTAACC

CCGGAACCGACCAGTGAGACGGGGCGACAGCTACGGCTATTCCTTGAACCCCGTTTCGAA

GCCATTGAGCAAGACGGCTTGGTGGTTCGTGAGAGCCTGACCAAACTGCTCTCAGAAACG

GGCATGACGGATAGCGGCGACAACATCAAGGCGCTCAAAGCCAGCCTGCTGCGCATGTCG

AACGTCACCATCCTTGTGACGAAGGGACGGCGGCAAGCCGCGTTCCACCTGATGAGTCAT

GCTTTTGACGAGACGGACGGCAGGCTATGGGTTGCCCTGAATCCGCGTATTGCCGAAGCG

ATCCTGGGGCATCGTCCATATGCCCGTATCGACATGGCGGAAGTGCGGGTGCTACAGACT

GATCCGGCACGGCTGATGCACCAACGGCTATGCGGCTGGATCGACCCCGGCAAATCCGGG

CGCGTGGAACTGGACACGCTTTGCGGCTATGTCTGGCCAGATGAAGCCAATGCCGAAGCT

ATGAAAAAACGCCGTCAGACTGCCCGGAAGGCACTGGCCGAACTTGCCGCCGTGGGTTGG

GTAGTGAACGAATACGCCAAGGGAAAATGGGAGATCAAGAGGCCTGGCCCCACGGCAACT

GCACCCGTTTACCGTCGTAACGTTCCCTTGTTACCGTCGTAA

>GR34_CP047975

ATGAGTAACATAGTATATAAAGATAATAATTTGGTTGAAGCATCGTATTCACTAAATCTA

TCTGAGCAAAGGCTAATATTAATTGCGATCATTGCTGCTCGAGAAATTGAGAAAGAACTT

ACTTCTGATACTATACTTACGATTCATGCTTCTGAATACATGAAACAGTTTAATTTAGGA

CGTCAAGCATCTTATGAAGCGTTACAATCTGCATGTGATAATCTTTTTGAACGGCATTTG

AATTATAAAGCAGTAGATCCTATAACTGGAAAAATAGGAATATACAAAAGTAGGTGGGTT

TCAAAAGTTGGTTATGTTAAGGAAGAGGGATGCGTCCAACTAATTTTTGCACCAGATATT

ATCCCCTTATTTGTAAAACTTGAAGAAAAGTTCACAAGATATGAACTTAAACAAATCTCA

CCACTTACAAGCATTTATGCTATTCGCCTGTATGAGTTACTCATACGTTGGAGATCAACT

GGTAAGTTATATATTTCTATAGATGAGCTTAGATCAAAACTCGGATTGATAGAAGATGAA

TATAAAAAGATGGGAGACTTTAAAAAAAGGGTTCTTACCGTAGCTTTAAATCAAATTAAC

AAATTTACGGATATTACTGTTTCTTATATACAAAAAAAGGAGGGAAGAAATATTTCAGAA

TTACATTTCATGTTTGAAGAAAAAGAACAAAATAAAACGAGTACAAGTGCTCCTTAGAGC

CAACTTATAAATTGACAGCTAAGCAATGTATATTCTTTGCTAAAAAGCTATGTGATATTA

CTAACTATCCAAAATTTGGTAATGATTTCGCACATCGTGGAGAGACTCTAGAAGACTTTC

AGGAAAGGATATCAAGTGATCTTCTTGATAGCGATAATGTTAGAAAGTATTTTTCTTATT

TGTTAGAAGTTGGTTATGCTCCTAAATACAAGAAGTAA

>GR35_CP050420

ATGAAGAATGGATTAGTAGTGAAAGATAATGCCTTGATGAATGCTAGTTATAATTTAGAA

GTAACAGAGCAACGTCTAATACTTTTAGCAATTATCAATGCACGTGAAACACAGCAAGGA

ATTACGTCAGATAGCAAGTTAGAGATACATGCTAATGACTATGCCAATCAGTTTAATGTG

AAGAAGGAAACCGCTTATGAAGCATTAAAAAATGCGGTTAATAATTTGTTTGAACGCCAG

TTCTCCTTCAAAGAAACTACAAAAAAAGGGGTTGGTGTAGTCCGATCTCGTTGGGTAAGT

AGAATCAAATATATTGATGATTCAGGTCTATTAGAAATCACTTTTGCCCCTGATGTTGTT

CCATTAATCACAAGGCTTGAACAACATTTCACTAGCTACCAGTTAAAACAGGTTTCACAG

CTCACCAGTAAATACGCTATCCGCTTGTATGAAGTTTTAATCGCATGGCGTGAAGTTGGC

AAAGTGCCTAAGATTGATCTCGCCGAATTTAGAGAAAGGCTAGGTATTGCAGCTAATGAA

TATAAAGCAATGAATCACTTCAAAAGCCGTGTACTAGAACCATCTATCAAACAAATTAAC

GAGCATACGGACATTACAGTAAGTTATGATCAACATAAAACTGGACGTACTATTACTGGC

TTTTCCTTCAAATTTAAGCAAAAGAATCCTCTAAAAATTACTAAGCAAACAGAGATAAAA

AGAGACCCTAATACCCCTGACTTTTTCATTGAGATGACAGATGCTCAAAGATATTTATTT

GCTCAAAAGCTATCTGTACTACCTGAAGTTGGGAGCGAATATGCAAAGATTGGGGAATCA

TCTGAAGACTTTGTTAAACGTCTTGCAGATATGCTTTTAGAACCAAAAAAAATCAAAATG

TTTTATCCATTGCTGGAACAGCTCGGTTTTAATTCTAAAAAACAACAACCGATACAAAAA

CAGGTCGATGAACCTAAGTTTTTAGATTTATCTGATATGCAGATCAATCGAATTTCTTAT

GGCATGTCCAGGCATAAAAAATTTGCTCACTTGAAACAAGAAGACGAATCGCCTGATGAC

TTCATGCAAAAACTTCGCATCATGTTAAAAGACCCAAAACAGCAAATTCAATTTTATGAC

TACCTAAAAGCTAATCCTTAA

>GR36_CM016516

ATGGATCAAGCACTTATAACACAAGACAGTTCAGTTATTAAACAGGCGAATAAGCTTATT

GAATCTGTTTATAGGATGGATGCAAATGAGCAAAAAATTATCTTGCTTGCTGCGAAATTC

GTATATGACATGGAAAAGAAAAAAGAAGAATTTACGACAAATACTGAAATTGTTATTACG

GCAGCAGAATACGCCAATGAATATGGCATTACTCGTCAGACTGCTTTTGAAGTTATAAGT

AAAGCAAAAAACACGCTTTATGAACGTTCTTTTGAGTATGATTATAAAAACCCTGAAACG

GGCGAAGTAAAACCGATGAGTAGCCGTTGGATTCATTCTAAAGGCGAAATGAAAGCAAAG

AGCGAAATTAGTATGTTCTTTGCACCTGCTGTTATTCCACTTATCTACCTAGTGCAACAA

GAGTTTACGCTATTAGATATTAAAGAAATTGGCCGACTAAAAAGTAAATATGCTATCCGG

CTGTATCAGATCCTCATGAAATGGCGAAATGCTGATTTTCAACCGAAGTTTGAATATCAA

GACCTTCGGGCCAAGTTGGGTGTTGAAGATGGCGATTACACACTTATGGCTGATTTTAAA

AAGGGTGTAATTAACGTTGCTGTCAAGCAAATAAATCAGGGTACTGGCTTTGTTGGGTTA

AAATATTCAACCGTAAAAAAAGGTAATACAATTACCCATTTCACCTTCTCTTATGACAAA

TATGACAATAAAACAATTAATGTCACTCCAATTAACTCTCAAAGCGCAACTAGGCCTAAA

AAACCTAAAAAGGCAAATCAGCAGCTTACAGAGCAAAAAGCCCCAAATAGCGCAAATGAG

AGTGATTTAGGCGATGATTTTAGCTTAACTCCACCAGAGCCTAAGGCGCTTAAAAAAGTG

AAGCGCCACTTTGAAGGAGGCATGACCGAACCACAGGCTTACATGTTCGCTGGAAAAATC

GTTAATAAAATTAAGGAAGGGGATACACGCTTCATGTATCTTTCAAAATTGGCTCAAGAG

GGTGAGCTTGATTCTAGTTTTTCAAAAAGGATTGCAGAGGACTTTTTAATTGGGAATTTA

GAGCCATATCAAGAGGCTTTAGGTTATCTTGGATACAAGCATCACAAGTAA

>GR37_CP039030

ATGAAGAATGACTTAGTCGTAAAAGATAATGCTTTAATTAATGCCAGCTATAATCTCGAA

GTAACAGAACAACGACTGATCCTATTATCAATTATAAGAGCAAGAGAAACAGGCCAAGGA

ATTTCATCAGACAGTAAGTTAGAAATCCATGCTTCTGATTATGCTTCTCGCTTTGATGTG

ACTAAAGAAGCTGCTTATAATGCACTCAAAAATGCGGTTAATAATCTTTTTGAAAGGAAG

TTCTCTTTCAAGGAGATCCATAAAGATACAAACAAAGAAATTGTAGTCAAATCACGTTGG

GTTAGTAGAATTGCATATGTTGATGACCTTGCTATATTAGAGGTCACATTTGCACCTGAT

GTTGTTCCTCTTATTACAAGATTGGAAAAGCACTTTACAAGTTATCAACTCAAACAAGTT

GCCCAACTCACTAGTAAGTATGCTATTCGCTTATATGAGTTTCTAATTGCTTGGAGAAGT

ACTGGAAAAACGCCAATCATTTCTTTATCTGAATTCAGGGAAAAATTAGGACTTGATATT

AATGAATATCAAAAAATGATTAATTTTAAAAACCGAGTCTTAGAACCAGCAATAAAACAA

ATTAATGAATTGACAGATATATACGTGAAATATGAGCAGTACAAAACTGGAAGATCCATT

TCAGGCTTATCATTCACTTTTAAGCAAAAGAAAGCTGAATCTTTATCACAAAAAAAAGAT

CTTAATACATTAGATTTATTCACAAAAATGACTGATGCTCAGCGCCATATGTTTGCTAGT

AAACTTTCACAGTTACCTGAAATGAGTAAGTATTCTCAAGGTACAGAAAGTTATCAGCAA

TTTTCTGTACGTATTGCAGAGATGCTGCAAGATCCTGAGCAAATTAAAGAACTATACCCG

CACTTAAAAAAAGTGGGATACATACCATCCAACAACAAGGACACCGTAAATGGCTAA

>GR38_CP012007

ATGAAAAAACTGTCAGTTTCAGAGTTAGCTAAGCTTTATGGATATTCAAGACAAGCCATA

TATGCACATATAAACAAAGGAAATTTATCTAAAGGATCTGATGGATTAATTGACTTTTCA

GAGGCCCTAAGAGTTTTTGGGGAACCACAAAAAAAAGAGGATACAGTCAATCAAAGTCAA

TCAATTAACAGTCAAAACTTGACAGAAGTTGACTTACTAAAACGTCAAGTTGACATACTG

GAAAAACAATTAAATCAGGCAATACAGAGAGAAAATCAATCTTTAGAACGTGAATCGTTT

TATCAAGAACAGATTGAAGCTATGCAGCGCTTACTAGAAGCACCAAAAGCCAATATGACT

ACCTTTACCGATCAGAGCTTTAAACAGGATATAGCAACGGATCCTCGGTCGGAACTAGCA

ACGAACTATGACGAATTGACTACTCCTCAGCAAGACAATAAGCGTATTCCTATCCCGGAG

CATGTTGAACCAGAACAGAAGAAGAGAGGCTTCCTAAGCCGCTTTTTCCTTCCATATGGT

TAG

>GR39_CP034097

ATGCCTACCAGTGAATTAATAGTAAAAGATAACGCTTTAATTAATGCGAGCTACAATTTG

GATACCGTAGAACAACGGTTAATTTTACTGGCTATTTTACAGGCAAGAGAAACAAGAACA

GGGATTGATGCCAACACTAGGCTACGGATCCATGCCAGTGATTATATGAGCAGATTTAAT

GTTAACAAACATGCCGCCTACAAAGCTCTAAAAACAGCAGTTACTAACCTTTTTGGTAGA

CAGTTTAGCTATCAAACAATTGATGAAAAAACAGGAAAATCAAAGAAAGTAATTTCTCGA

TGGGTTCAAAATATTTCTTATATTGATGATGCTGCAACCTTAGAGGTTACTTTTACAATG

GATGTAGTACCTTTAATTACACGTTTAGAAAAACAATTTACAAGTTATCAGTTAAAACAA

GTTACCCAATTAACAGGAAAATATGCCATTCGACTCTATGAGTTGCTTATAGCATGGCGT

GAAGTTGGTAAGACTCCTATATTTGAAGTTTCTGACTTTCGTTCAAAGCTTGGCTTAACT

TCAGATGACTACCCAAGACTAGATACATTTAAACGACGTGTACTTGAATCAGCAGTTAAA

CAGATAAATGAACATACAGATATCATTGTTAAAGTTGAACAACATAAAGAAGGTCGCTCT

ATTTCTGGGTTCTCTTTTAGCTTCAAACAAAAACGAAATCCGAACAAAGCAATAGAACTT

AAGAAAGATCCCAATACATTAGATATCTTTTCCAGAATAACAGATGCTCAACGCCACCTA

TTTGCTAATAAGCTTTCAAAATTACCTGAAATGGCGAAATACTCTCAGGGCACTGAAAGC

TATCAACAGTTTGCAATAAGAATTGCTGAAATGCTTCAAGATCCTGCTAAGTTTCAAGAA

TTACATCCTTATCTTGAAAAAGTTGGCTATCGTTAA

>GR40_CP034094

ATGAGTGATCTAATAGTTAAAGATAATGCTTTAATCAGTGCTAGTTATAATTTGGATGTC

ATTGAGCAGAGAATTATTTTACTTTCGATTATTAAAGCTCGGGAAACTGGCACAGGAATA

GATGCCAATACATCTTTAGAAATTCATGCAAGTGATTATGTAAAGCATTTCAAAGTAGAA

AGACAAACAGCTTATGAAGCCTTAAAAACAGCTGTAAATAATTTATTCAATAGACAGTTT

AGTTATACGCAACCATTTCAGAATACAGAAAGAGTGGAATATGTAAAATCTAGATGGGTT

AGCCGAATATCGTATGTTGATGAGTCTGCGATATTAAATATTACATTTGCTCCAGACATT

GTCCCTTTGATAACTAAATTAGAAAAGCATTTTACAAGTTATCAATTAAAACGTGTTGCA

CAACTTACTAGCAAGTATGCGATACGCTTATATGAATTACTGATCCAATGGCGTGAAGTT

GGAAAGACTCCAATATTAGAGATTAATGATTTTCGCTTTAAATTAGGTATCGAAAAGCAT

GAATATAAACAGATGGGGCATTTCAAGAGTAGAGTACTTGATCCTGCTATACAGCAGATT

AATGAGTTCACAGATATTAAAACTGAATATGTGCAGCATAAGTCTGGTCGAGTAATAACT

GGTTTCGAATTTAAATTTGCCTTAAAAGATTCTTCCTCTGGAGAAGAAAAGACGAACAAT

AAAAATATTCTCAATAAGGAAGTGGTAAGTATGTCTGATCGACAGATTTCACTTTTTGCA

AATAAGCTAGCTTATGATGAGGAATTTGCTAGTCGTATAGCAGAAATAGGGGAAAGTTAT

GAAGATCTAGAAAAGAGGTTAGTAAAAATGCTAACTAAAAAAGAAAATTTGGTTAAATGG

GCTAAGGATCTTCAGCGTATAGGTTTCAAAGCTAATTAG

>GR41_CP051870

ATGCAGAAAATTATTGGCTCACAAGGCTTAGAGGACTATACACAATCTAATACAAGGGCA

AAATTGACCGATTTAGTTGTGACCCGCAATGATTTTCCTACTGCCAGATATAGCATTGAT

CTCAATCTTGAGAAGCTTATGTACTGTGCAATGATCATCGTGAGAAAGAATGAGCTTAAA

AATAAGACCTCAATTACTCATGACGACTTCATTTATGTGAGCAGCGAAAACTTTGGAGAA

TTGACCTCCCCAATGGCTAGAAAGGAAGTGCTTACTGCAACAGATAAACGTGAAATTCAG

CGAAATGCTGAAACTGCTTTAAAACGTATTTATACAAAATTTGATAACCCAACTATGTTG

GTTAAAGATGGGGAATCTGACGAACCTGCTAAAGTTCCGATGATGACTTATTGCCATTAT

GACAAAGCAACTAAATGTATTAAGGTTAGATTCGCAAAAGAATTTTTTGAGTATTTCTAT

GATCTAGTTAAGAAAGTAGATGAAAAAACTAAGTCATTTAGTAGCCATGAACTGAAGCAT

ATTATCTTATTTAATTCGAGCTATTCCCTTCGTTTGTATCGGATCTTAATGAGCTATATG

TGGCGTACATCAGAGGTCACTATTGATCTGGAAGAATTAAGATGGATGCTTGAATGTGAG

GATAAATATAAAGAGTTGGCCAATTTTAAAAACCGTGTTTTGAATGTGGCTCAGGATGAA

ATTAACGAACTTAGCAATATTAATGTCAGTTTTGAGAACGTAAAAAACGGAAAGGAAGTA

GTTGCTATTAAGTTTATTTTTAGCATGAAAACTGAATATAAAGAGCAAGGACACATCAAA

TTTATAGATAAAATGAAAAAGGGCTATCTGGCTGCTGCAATTCCATTTAGTGATGATGGA

TCACACTTTAAAGCACCTGATCGTATTAAGCATTTCAAACCACCAGTAAAGGTATCTCCA

AAACAAATCAGCACCTTAGTAAATTGCAAAGAATTTTTAAATGATTACGGATATTTCTTA

GGTAATCTAGACGAGGATACTTCTAAGGTAATCATGAGAACTCTATTAACTGAAAAGTTA

GATAAGCTTAATGCTCATAAGCCGATAGATATGGATTATTACTTCTGGTTACAGGCAAAA

CGAGGGATTATCACCAATAGCAATAATGATAACAAGAACGATCAGGACACAGACAATCAG

GACACGGACGATCAGGATTAA

>GR42_CM003909

ATGGCTAAGTTATCACTAAGTGAAGTATCTAAAAAATTTCATGTGGATAGATCAACCATT

TACAGAGCTGTACGTAATGGACGTTTATCACGCTCCAGTGATGGACAATTCGATCTAGCA

GAGGTCATACGATGCTTTGGAGAACCTGAGCAAACATCTCAAAAAATTGAATCATCTAAG

CAAGAAGGTGATGAATCTACAAAAAAACTTATTGCCCATTTAGAAAATGAAGTCAAAAAA

TACCAAGAACGTGAAGAACGGTTAATGCAACAAATTGACCGTATGCAAACACTCATTGAG

CTGAAAAGTGTTGCACCTGCCACAGCAGCACCACACCAAGATGCTACGGCATGCGACACC

AAGATGCCACAGCATGCGACAACACAACAAGACACTGATAACAAAAAGAATAATGAATTA

AATATTGCAGAAAATGTAGCAGTGCCACAGCAAGAAACTACGGCATACCACACCCAAACG

CTACAGCATGCCACGTTGCAAAGTGTGGCAGTGCCACAACACAAAAAACGTGGTTTATTT

GGCCGTGTGCTGAATGCCGTCTTTGATAATGACTGA

>GR43_CP010400

ATGAAAAGCCTTACAGTTCTTGAGCTTTCTAAGCTTTACAATATCAATAGACAAACGATT

TACAACAACATAAAGAAAGGAATTTTAAGTAAAAATTCTGATAATAAAATTGATCTAGCC

GAGGCTATCCGTGTTTTTGGTGAACCTTCAAAAAAACAGGATGTAAAAGAATCTGTAAAA

ATAGACAGTCCAATATCGACAGAAGTTTTACTACTTAGACAACAGATAAACATGCTGCAA

AATCAGCTTGATGATGCAAAAGAGCGTGAATCCTTTTATCAAAATCAAATTGAGACAATG

CAACGTCTACTGGAAGCACCTAAACCTGAGATCCAAGAGCCTAGTGAACCCGAACTCCCT

CAATCAGCTCCCATTGTAGAAATGAAGGAGCAGGTGATAGTTGTTCCACCTAAAGATGAC

GGATTGACTACTCCAGAGAATAAGCGCATTCCTGTTCCAGAGCATGTTGAACCAGAGCCT

AAAAAGCGTGGCTTCTGGAGTCGTTTTTTTCTTCCATATGGTTAA

>GR44_CM009044

ATGATTGATTTTATAGAAATGCGCTTATTCGTCTTAGACGAATTTGTTATCTCGGATAGG

GATGGCAAGCACTTTCTATTGTCTTGTGATTTGTTACAACTTGGTGTCACTGTAGGTTCA

AGAGATGTTTACTTGGATGAGCAGGGCAATATGCAAGTAGGTGCGTTGTATCACCCTTAT

GATGATTTACCCACTTCATTTACTAATGTTGCTTTTAAATTAGTTCATGAAGGTAAAATT

AAACCGCATGTCATGATCAAATGTAGTCCTGCAAAGATTATGCAGGGTCATAACATTTTT

GGCTCGGATAATTTGGAATTAGGTGTTTTTGAAATGCTTGGTTTCTTAGCGGAATCTCAT

CCTAAGCTTTATAAAATATTAGATATTCCGAATGCTCAGATTGTCAATTTAGATGTGACT

TATTCAGCACGTTTAAGAAATGATGATCAAGTATGCAAGGTTTTAGACTTTCTTCGTAAA

GTTTCCAGTGGCTCACTTCGTAAATCTAAATTGGTTTATGGTTCCACAGTTTATTGGGGT

TCACCTAATTCTAAACGCTTGTGCCGTAAGGCTTATTGCAAGTCAATTGAATTTCAATTG

CAGCTTGCAAAGCTAAAACGTCAAGCTTCTAAAGGTGAAGTTTTTGCGTTGCGTGTAATT

AAAGCAATGGAAGACCCTCGTGTAATTGAATTTATGCAGGGTTTACTACGTTTAGAAACT

CGTTTTAAACCTTTGTGGTTAACGGAACATAATATTCCACTCAATGTATTTGATCTTATT

AAATATCAATCTGAACACCCTAATTTTTTAACTGATCTTTGGCAACTAGCAAATAAACCA

CTTTTTGAAGCATTGGAGGGTCATACGATGAAGGCTCTTGATCACGATACTGTATTTGGA

AAAATCTGCGCTAAATTCGACACTTATACAAAGTCAGGTCGTTTATCACAAACTAAGTCT

CGTAATATTTTTAATTTCTTTTGTGCATTGGAACTTCATGGTTCTGATGAACTTAAGAAG

AAATATAGTAAGTCACAATATTATCAATATATATCAGATTTAATGAGTTGCGGTTTTTCA

AAGGCTTATTTGCAAAACCTTGATTCAGAATCAAAAAATAACGTTATTCCATTCGTTCAG

CTCGTCAAAATCGATTTTCAGAATCAAGTACCAGATTGGTATCAAGAACCTGAATCGCGC

TTTGCTAAGGTAGGTTAA

>GR45_CM004454

ATGGCAAAATTATCACTTACTGAGGTCTCAAAACGCTTTAAGGTGAGCCGTTCAACTGTT

TACAGAGCGATCAAAGAAGGAAAGCTATCTCGAAGTGCTGATAATCAGTTTGATCTGGCT

GAAGTGATCCGGTGCTTTGGTGAGCCGGTAATTAAGGCATCTCCTGAAGAACAGTCTAAA

GCTCCAGTAAAAGACGATGCTGATTTACGTCAGTTAGTCGACTTTATGAAAAGAGAGATT

GAATCTTATAAGGAACGTGAACAGCGTTATTTGGATCAAATTGACCGGTTCCAGCTTTTA

TTAGGACACAAAGAGTCTTCTGAAAAAGTGTCTCATGACACACCTGTGACACGTTCCAAC

GATACACCATGCGACACTTTCAATGACACACCAGAACCTATTGATACACAAGCAATGATA

CAAAATGAGACGTCTCATGATACACCTTTGACACGTCTCAATGATGCACCACGAGACACT

TTTGAGACGTCTCATGACACACTAAAAAAACAACCTAAAAAGCGTGGCCTACTTGGTCGC

GTTATTAATGCGTTTTTCAATGATAATTAA

>GR46_CP051873

ATGTTAGACAAAATCGTGATGCACATACCAGTTGATGCTTCGCTAGTCGATATCGATAGC

GAAGGTCGGTACTGTGTATTTGGTTTTGATCTGCTAGACCTTGGTTTAAGAGTTGGCTCT

TACGATGTATTCAAAGACGAAGATGGGAACGTTAAACATCAGGTTCTAAATCATGCATAT

TCTAAACTTCCGACTTCTTATACGAAGATGGCTTTTAAGTTCTTTCATGAGGGCCGTACT

TATCCTTATGTTGAACTTAAAGCTAGTCCTGCGAAGATTTTGCAAGGGCACAATGTGTAT

GGATCGGATTGGATAGAGCAGGGTGCGCTTGAAATGCTTGGTTATCTGGCTGAGTCGCAT

CCAACACTTTACGGTATGTTGGCCATTGGTGAAACGGAAGTTAAACAGTTAGATGCTACT

TATTCAGCACGTTTACGTGATGATAACCAAGTCGCTGATGCTATTGACTTTTTACGTAAT

GTAACTACTCAACATATCCGTAAATCAACCAAGCAAGTTACTTATAAGAACACTAATTAT

TACGGTTCTGAACGTTGTAAACGTTTTGCCCGTAAAGTTTACGGTAAATCTGCTGAATTT

CAGTCTCAACTTGAAGAGCAAATTAAGCTTGCTAAGGCAAATGATAAATGTGCTCAACGT

GTTGTAGCTGTTATGTCAGATCCAGATTTACAGCAGTGGGTTAAAGGTCTCTTGCGATTT

GAAACAGGCATTAAAGCCTACGTTATGAAAGAACTCGGTATTCCGACTAATTTATTTGAA

CTTATTCGTTATCAACGCTCTAACCCTAATTTTTTACGTGATATCTGGATTAAAGCAAAC

GCTCAGTTATTTAAAGCCCTAGAGGGTACAGCCATGAAAGCTACTGACCACGATACTGTA

TTTAAAAATCTCTGTAATGTTTTTGGCACTGTGACACCTAAAGGTCGTCAAAGTTTTACT

AAGGCTCGTAATGTCTTCAATTTTTACTGCGCGTTAGAAATGAACGGTTATGAGGTTATG

AAGACACGTTATGGTGAGCGTCAGTACTACCAATATATGGCTGATTTAATAGCTGCGGGT

TATTCAAAAGCATTCTTGCAGAATCTACATGTTGAGTCTAAATCCAACATTATTCCATTT

CTTAAACTTGTCGAAATTAATTTCGAAAATCAAGTACCTGAGAACTTTAAAGAACCAATT

TCTACTTTTAATCAGCGTGAATTACGCATCGCATAA

>GR47_CP008709

TTGTACGCTAATACTCAAAATACGCAAGCAACTGCGGAGCGTGGCGTTCCGCGAACGCAC

ATCGCTCCGTCAGTTGATGGCGGCTCCCTTGGTATATATACGGATAACTCCGCCAACCGC

ACTACACAAGGCTTTCAGCCCATACGAGAAAAGTTTAAATTACTGCATTTTGCTCGAAAA

TTACTCCCTAAAGAACGTACTGCTCACTGTTTCTATAATCGGATTACAAAAGAAGAGGGT

GTATCTGTTTTTTTGAATAAATTACGGAATAAAGCCAATTATGGGAATGTGATGCGCTGT

GCTAATCCCTGGGCTTGTCCTGTTTGTTCTGCAATTATTTCAGAAGGTCGAAAAGATGAA

GTAAAAACGGCTATGGACTGGTGGAAAGCTCAAGGCGGTGATGTATTACTTCTTACTCTA

ACAGCTCCCCACTATTCGACTACTGATATTAAATCTTTAAAGCCTGCAATGCTTAAAGCT

CGAAAATATATGCTCAAGGGTGTTCGGGCGACTAAAGACCTTTTCAAACACTATGCCATT

GAACATTACATTTCTGTATTTGAAGTGACACACGGAAAAAATGGATTCCATCCACACTTC

CATATTCTGTTATTCACTAAGTATCACGTACACAATCCACGTGGTTCTGTGATGCGTATG

CAATTCTTTGAACAATGGAAAAAGGCATGTGAAAAAGCAGGATTAGACCAACCAAGCTAT

GAACACGGTTTGGATCTCCGCAATGGTCAAAAGGCAGCAAGCTATGTTTCAAAATGGGGT

TTGGAACATGAAATGACAAAAGGACATATTAAAAAAGGTAAAACAGATTCTAAAACGCCT

TTCGATTTACTCCGTGATTATGCAGAGGGTGACGAAAATGCAGGAAAATTATTTAGGATT

TACTTTGATGCTTTCAAAGGTACACGCCAATTAAATTGGTCAAAAGGTTTGAAAAAACTC

TCCAGCAAAGGGCAAGAAGAAAAAACAGATCAAGAATTAGTTGATGAAACGGACAATGTT

GCGGAGCTGATGTTCAAATTATCTATAGAACTATGGAATCCAATACGGAAACACGGCAGA

CAAGGCGAATTGCTGATTAAGGTGCAAGAAGATCACACACTTAAAAAGGCAATGGAATAT

GTCAAAGAGTGCTTAGGCTATGATGGACAACTGAGGGAATAA

>GR48_CP034096

ATGAGAACAGAATTAGTCGTGAAAGACAATGCCCTAATCAATGCTAGTTATAACCTTGAC

CTAGTCGAACAGCGGTTGATTCTTTTAGCAATTGTTGAAGCGAGAGAATCTGGACGAGGC

ATTGATGCGAACAAACCTCTCACAGTTCATGCATCAAGCTATATCAGTCAATTCAATGTA

GCTAGACAGACAGCCTACCAAGCGTTAAAAGATGCCTGTAAAGACCTTTTTGCACGTCAA

TTTAGCTACCAAGAAAAAAGAGAGAAAGGGGTTGCAAATGTTACTAGTCGATGGGTTTCC

CAGATCGCATACATTGACGATACAGCCAGTGTTGAGCTGATCTTTTCACCTGCTATTATC

CCTTTGATTACACGTTTAGAAGAACAATTTACAAGCTATGAATTGGAACAAGTGAGTAAC

TTAACCAGTGCTTATGCTGTCCGACTATATGAGCTTCTAATTGCATGGCGCAGCACTGGT

AAAACGCCTGTGATCGAATTATCAGAGTTTCGGGCAAAAATGGGAGTTCTAGACGGTGAA

TACACTCGATCTGACAATTTTAAAAAGTGGGTTATAGAAAAACCGATTGAACAGATTAAC

GAGCATACAGACATCACGGTCAAGGTTGAACAACATAAAACAGGGCGTTCGATCACTGGT

TTTTCTTTTAGATTCAAGCAAAAACAACAACCAAAAATTGAAAAACCGATTGATCCGAAG

CGAGATCCAAACACACCAGATTTTTTCGTAAATATGACCGATTCACAACGCTATTTGTTC

GGTCACAAGCTATCTACACTGCAAGAAATGAGCGAATATTCTCAAGGTACTGAATCGTAT

GAGGATTTTGCCAAACGCATAGCGGAGATGCTTTTAGACCCTGAAAAATTCAGGACGTTT

TATCCGCTACTGGTGCAAGTCGGTTTTAAATAA

>GR49_CM009050

ATGAAAAAAGGTAAACAGCAGATGACCAAAGTTGTGAAAGATAATGTTTTGATTAATGCA

AGCTATAGTCTTGATCTTGTTGAACAAAGAGTTGTGCTTCAAGGTATCGTTAAATCACGC

GAAACTGAAAAAGGCTTTACAGATTCAAATCCTGTCAGCATTCACGCAAGCGAATACGAA

AAGCAATTTGGTGTAACAAAAGATGCAGCTTATAAAGCTTTAAAAGATGCTGTGCTGTCA

TTGTTTGAACGTCAATTCACATTCACAGAATTAGAAAAAGGCAAACTAAAAGTCGTTAAA

TCAAGATGGGTATCTCAGATCGCTTATGTTGATGATCTTGCAGAAGTGCAGATTATCTTT

AGTCCCGCTGTTGCAAGCATGTGTTCACGTTTAGAGAGCCACTTTACAAGCTATGACTTA

GATCAAGTGTCTAAGCTTAATAGTAAATACGCTGTACGTCTGTATGAGCTTGTTATAGCT

TGGCGAGCTACAGGTAAAGTACCTGAGATTGAGCTATCAGAATTTAGGAATAGATTAGGC

GTATGCGATAATGAATACACAGCAATGAACAACTTTAAAAAATACGTGCTAGACCTTGCG

GTATCACAGATCAACGAACACACAGACATTAAGCTCGACTACGAGCAACACAAGAAAGGG

CGTACAATCTCAGGCTTTTCATTTCGTCTGAAATCAAAAGTTAAACAGCAGAAGATTGAA

CAGCAACGCGACCCAAACACAGCCGATCTATTCACCAAAATGACAGATGCTCAACGTCAT

TTATTCGGCAACAAACTAGCTCACGATGCACGTGTGCAAAGTGATTACAGCCACTTAATC

GGGACTGGAAGCTATGAAGACTTTGGCAGATTGCTTGCTGATATGCTTGCAGAAGAAAAA

CACTTCAAAACATTCTATCCGCTACTTGTTGAGTGCGGTTTTAAATAG

>GR50_CP042559

ATGAGTAAAAATATAGTCGTAAAAGCGAATATCTTGATTGAGGCAAGCTACAAACTTGAT

TTGGTAGAAATGCGCTTAATACTTTTAGCAATTATTGAAGCTAGAGAAAGTAAAACATTG

ATTGATGCTAACTCTCTGATAAAAATATCAGCTAAAAAGTATGCAGAAATGTTTGATATT

AGCCTCAAAAACTCTTATTCCATGCTACGAGGGGCTTGTAAAACCCTGTTCGACAGACAA

TTTTCATATGTGGATTGGGATTTTGATCTATTAAGTGAAGATACTTCTAAAATCTTTTTT

ACTTCTCGATGGGTATCTAAGGTAGGTTATAAGCCAGGTTTTGTTTACTTATCTTTTGCT

CCTGATGTCATACCATTAATAAGTCGCTTAGAAAAAGAATTTACGCGTTATGACCTTCTT

CAAATAAGTAAGTTTTCTAGTATTTATGCTGTTCGGATTTATGAAATATGTATGCAATGG

AAGATTGCTAAAAAGTTTTATATTTCTCTCTCTGATTTACGAAATACATTAGGTTTAACA

GAAATAGAACTTTCAAGAATTGATAACTTTCAAAAGAAAGTCCTTGAAGTAGCAAAAGAT

CAAATTAATGAGTCTTCGGACATAACTATTGATTATAAAACCAAAAAGTCTGGAACCAAG

ATAACTGGCTTTGAGTTCACAATAAGATCCAAAACATCACAGCCAATTAAAATGGAAATC

GCCTTAAATGATAAACAAATTCTTTTGTTCTCAAATAAGCTAGTTCATGAACCTTCATTT

GCTTCAAAGTACAGCAAAGTTGGTGAAAGTTATGATGACTTCGCTATAAGAATTTCTGAA

AATTTAACTATTCCTGATAAGGTTCAGGAATACTTACCCTATCTAGAAAAAGTCGGGTTC

AATACTGCTTAA

>GR51_CP050408

ATGAAAAAAGACCTAGTCGTTAAGGATAATGCGCTGATCAATGCCAGTTATAATTTAGAC

CTTGCAGAACAACGCCTAATCCTTTTAGCAATACTGGAGGCGAGGGAGTCTAATATCCCT

AGTGACCGTGATTTAACTATCCATGCAGAAAGCTACATTAACCATTTCAATGTCCATAGA

AACACCGCCTACAAAGTCTTAAAGGATGCTTGCAAAAACTTGTTTGAGCGTCGCTTTAGT

TACCAAAAATTAACGGCTAAAGGCAATCTTGAGAATGTTATGAGCCGATGGGTACAGCGT

GTGTCCTATGTTGAAAATGAAGCCCTTGTCCGTATCAGGTTTTCAGATGATGTTGTGCCA

CTGATCACCAACCTAGAGAAGCATTTCACCAGTTATGAACTGGAGCAGGTAAGTAGCCTA

ACGAGTGCCTATGCCATACGTTTATATGAACTACTAATTGCATGGCGTAGCACTGGGAAA

GTATCAATGCTTGAAACTAAGGAATTGAGGTCAAGACTTGGTGTATTGGACACAGAGCAT

CAACGGATGGAATCGTTTAAAAGACGAGTATTAGAGCCTGCTATTCAACAAATTAACGAC

CATACCGACATTAAGGCAGAATACGAGCAACATAAGCGAGGACGTTCAATCATTGGGTTT

TCATTCAGCTTTAAACAGAAATCCAAGCCTAAAACAATCAACCATGAACGAGATCCAAGC

ACGGTTGATATGTTCTGCAACTTGTCAGACAGTCAGATAAATACTTATAGCTCAGTATTG

TCTAAAGTCCACAGTATCAGCGACCTAGCAGGGAATAAGGACTATCAAGCATTTGCAATA

TGGATTGCCAATATTTTACGTGACCCGTCCAGTGTGCGAGAAGAAACGGCAAAACGCATA

TTCAAGGCATTGCGAACAGAAACAGATTTTAAAGGTTAA

>GR52_CP053216

ATGTCAAATGATCTAGTGGTTAAGGATAATGTTTTAATTAATGCAAGTTATAACCTAGAA

GTCACAGAACAACGACTAATTCTTTTATCAATCATTACGGCAAGAGAAACAGGACAAGGA

ATAACCCCTGAAAGTAGATTAAAGATTCATGCTAGTGACTATGCAGAGCGATTCAATGTT

AGTAAAGAAGCTTCATACGATGCCTTAAAAAATGCAGTGAATAATTTATTTGAAAGAAAA

TTTACTTTTAAAGAACAATTAGATAACGGCAAAGAAATTGTCGTTAAATCACGTTGGATT

TCGCAAATTTCTTATATACCTCACCTGGCAATCTTAGAGATTATTTTCTCTCCTGCGGTA

GTGCCATTAGTCACTAGACTGGAAAGACATTTTACAAGTTATCAGTTAAAGCAAGTTGCA

CAACTTACCAGTAAGTATGCAATTCGACTTTATGAATTATTGATTGCTTGGCGCAGTTTA

GGGAAAACGCCCATTCTTGAAATCAGTGATTTTCGTCAAAAAATAGGCGTTCAAGATACT

GAATATAAAACGATGAGCGACTTTAAGAAAAGAGTTTTAGAGCCTGCGATTGAACAGATC

AACAAGGAAACAGATATTACGGTTAAGTATGACCAACATAAAACAGGAAGGGTAGTATCG

GGCTTTGAGTTCAAGTTTAAGATTAAAGGGGAAGCAGCTAGTAAAAAGACTACTACCCAA

AAGAAAAAGGTTGAGGAACTATTCTGTAAGATCAGTGATAAACAGGTTGAAATCTATTCT

AATAAACTTTCAAAACAACACGAATTAAGCGATCTTGCTGGCAATAAAGATTATGCTGCA

TTCGCTATATGGATTGGAAATATTCTAAGAGATAAGGACAGCGTAAGACCTGAAACAGCA

AAAAGAATATTTACAGCCCTTCATACATTAACGGACTTTAATAAGTAA

>GR53_CP042564

ATGGATTTGGATGTTAAAAAACACTATCCCAAAGACTGGATAGTTTTACAAAATAGAGTG

GTTGAATGCTTTAGAGGTATGTCCCTTGATGAAAAGCGTTTATTTATCATGGCAACCCCT

TTGGCAAGAACCACAAAAATATCAAGCAATGATCCTATTTTTATATCTTCGAGTGACTTT

TCAAAGGAGTGTGGCATTGACTTATCAACGGCTTATACAGCTTTAGAATTGGCATCTGAA

CGCCTATTTACTCGGTTCTTTGGTTACACCAATGCTGAGGGTGACAGGGTAAAAATGCGT

TGGCTAAACAAGGTGATTTATAAGGCAGGGCAAGGCGGTTCAGAACTATATTTTACAGAT

GAAGTACTTCTGTTACTTAGAGAGTTTGACGCCCTAAACCCTTATACCAAGTATAAAAAA

GAGGTCGTTTTACGGCTTAAAAAAGACTACTCGCTAGACTTCTATCACTTGGCTAAGAAA

CACCAAACAATGGGCGGTTTTCAAATCAGCCTAGACGAATTATTTCAGCAGTTGGGCTTG

CCTGAATCCTATCAAGACCTAAGCAACCTAAAAAAACGTGTAATTAAGCCATCACTGGAC

GAGATCACAGCCAACACCGACATAGACCTAAGCTATGAGAATGTGAAACGTGGGCGTTCC

GTGGTTGGCTTTAAGTTCACTGTCAAAGAAAAACCAAAGCCCAAAGTTATAGAAACAGGA

CGAGATCCAAATACACCTGACCTATTCCACAAAATGACAGAATCTCAACTTGATACGTTT

AGCAGTAAACTTTCAGAACTCCCTGAAGTGCAAAAGATGGCACATGCAGGCGAAGATATG

AAACCATTTATTGCTCGTATTCGTTCGATGCTCAAAGACTCTGAAAAACAAAAAACGTTG

CTTCCCCATCTAGCAAAATTAGGATTTAAATCGAAATGA

>GR54_CM009039

GTGAATAACGCTCAATTAAGCGATTTTTTTGAAAATCTTGCTCATAAACCATACTGTGCA

GATGATTTATTGTATGGCTTACAAATAAGACCGAAAAAAACGGCAATTAACATGCAATAC

ATACAGGGTAATCAGCCTTGTATGCTTCATTACTTCTTTTTTGATATAGATCGTTCAGAT

GCTGTCATGGCATGGCATGATGAAAATTTGCCAATGCCCTATTGGACAGCGCAGACCTTA

AAAAATGGTCATGCTCATATTTGCTATAAATTAGAAATTCCACTGTGTACCAGTGAATTA

GCGAGTCAAAAAGCAATAGCCTATGCCTCAAAGGTTCAGGCAGGACTTGCCAATAAACTT

GGAGCAGACGTTGGCTACAGTCATCTCATTACGAAGAATCCATTTCATAAAGATTGGCGT

GTTACTTTTTGGAGTGAACAAGCTTATACGCTCGATTATTTAGCCGATTTTGTAGAATTA

CCAAAGAAATTAAGCAAAAAACAGGAAGTTTCAGGGCTTGGGCGTAACTGCACTATGTTC

GATACAGTGCGTAAATGGGCATATAAAGCGATTAGAGCACATAGAGGTGGTATTTATACT

ACTTGGCTAGATGAAGTAGTAAAACACTGTCTAAGCGTAAATGAGGCGTTTTTAGAGCCA

TTACCATACTCTGAGGTCAAAGCTACGGCAAAGAGCATTGCAACGTACTGCTGGAAGAAG

GATGCTTACTGTTATCAAGAGTTTATAGATCGTCAAAGTCGTAAAGGGGCTAAAGGAGGA

ACGGCAAGTAACTCGTCTAATGGAGGTAAAGCTAGGGCAATGAAGTACACTGAGATTAGA

GAAAAAGCGATATCACTTCGCAGAGATGGAAAAAGTATAAGAGAGATTGCTGATAGTCTT

GAAGTATCAAAGTCAGCAGTAGCTAATTGGGTTAAACAGTAA

>GR55_CP042563

ATGAATAATGCTCAATTAACGGATTTTTTTACTAATCTTGCTCATAAACCATACTGTGCA

GATGAGTTACTGTATGGCTTAAAAATAAGACCGAAAAAAACGGCAATCAACATGCAATAC

ATACAGGGCAATCAGCCTTGTATGCTTCATTACTTCTTTTTTGACATAGACCGTTCAGAT

GCAGTCATGGCATGGCATGAACAAAATTTACCAGTGCCTTATTGGACAGCACAAACACAG

AAAAACGGTCATGCTCATCTCTGTTATAAATTAGAAATTCCTTTGTGTACCAGTGAGTTT

GGCAGTCAAAAATCAATAGCCTATGCTTCAAAGGTACAAGCAGCGCTTGCCAACAGACTT

GGTGCAGACGTTGGCTATAGTCATCTCATTACGAAGAATCCATTTCATAAAGATTGGCGT

GTCACTTTTTGGAGTGAACAAGCGTATACGCTCGATTATTTAGCCGATTTTGTAGAGTTA

CCAAAGAAATTAAGCAAAAAACAGGAAGTTTTAGGGCTTGGTCGTAACTGCACATTATTT

GAGAATGCTCGTAAATGGGCATATAAGGCTGTTAGAGACTACTTTCACCATCATAGTAGT

CTAGAGTGGGACAAAGCTGTTTTAGCGCATCTAGAGGCATTGAATCGAGAATTTGAAGTA

CCTTTGCCATATAGTGAAGTAAAAGCTACAGCAAAGAGTATTGCTAAATATTGTTGGAAT

AAGTTTTCGTATGCTGGATTTAGTGAGTGGCAGTCTAAGAATGCCGAACGTGCTAATGCT

AAAGGGGCTTGTTCTTTGGGAGGAAAAGCACGCTCACAACAATTCAATAATTTAAGACAA

CAAGCCTTACATTTGCATATTGAGGGTGTTAACAATACTAAGATTGCTGAATATTTAAAT

GTAAGCCGTAAGACGATTACTCGTTGGCTTAATCAGGTAGTAATTCAATGA

>GR56_CP030107

ATGAAAGATTTGATTGTGAAAGATAACGCTTTAATTAACGCAAGCTATAATTTGGATTTG

GTTGAACAGCGACTTATTCTGTTATCAATTATCGAAGCTCGCGAAAGTGGTAAAGGAATC

AATGCAAATGACCCTTTAACAATTCACGCAGAAAGCTATATTAACCATTTTAATGTTCAC

AGAAACACAGCTTACCAAGCGTTAAAAGATGCTTGCAAAGACCTTTTTGCACGCCAATTT

AGTTACCAAGAACAACGTGGGAAAGGGATTGCAAATATAACCAGTCGTTGGGTTTCTCAA

ATCGCCTACATAGACAATACTGCATCTGTAGAGCTTATTTTCGCCCCTGCAATCATCCCT

TTAATAACAAGACTAGAAGAACAGTTCACAAGTTATGAGTTACAGCAAATTAAGGGGCTA

AGCACAGCTTATGCAATTCGTCTTTATGAATTGCTCATCGCTTGGAGGAGTACAGGGAAA

ACGCCAATTATTGAAACTCAAGAACTTCGCAGGAAGTTAGGTGTTCTTGATGGTGAATAT

AAAATGATTGCTGACTTTAAAAAGCGAGTATTTGAGCCATCTATCAAGCAAATAAACGAA

CATACAGACATAACCGTTAAGTCGGAACAGCACAAAACAGGTCGTTCAATTACTGGATTT

TCATTCCGATTCAAACAAAAGGCACAGCCAAAATTAGAACAAAAAACCGACCCTAAAAGA

GATCCAAACACACCTGATTTCTTTGTCGAAATGACCGATGCACAACGCCACTTATTTGGG

AACAAACTAGCCCATGATGCTCGTGTACAAAGCGAATACAGCCATTTAATTGGTACTGGA

AGCTATGAAGACTTTGCAAAATTACTGGCGGATATGCTTGCAGAAGAACAGCATTTTAAG

ATGTTTTACCCTTTGCTTGTAGAGCATGGTTACAAAGCATAA

>GR57_AFDB02000005

ATGAAATCACTATCTGTTATTGAGCTTTCAAACCTCTACGGAATAACTCGTCAAGCCATT

TATAAGCAAATTAATAAAGGTAATTTAAGTAAGAACAGTGACGGTAAAATTGATCTTGCC

GAAGCTATTAGAGTCTTTGGTGAACCATCCAGAAATGTAAACAGTTCACAGACAACTGAA

ACACGAAAGTTGTCAGAAGTTCACCTGCTAGAACAACAGGTTTACATGCTTCAAAAACAG

CTTGAACAGGCTCATGAGCGTGAGCAGTTCCAACGTGAGGAACTAAAAGCTAAAAATGAT

CAATTACACGTTAAAGATGAGCAAATTGAAGCGATACAACGCTTACTAGAAGCTCCGAAA

GCTTATATAACTAGTTCTATCGACCCGAAATTAGATATAGCAACAGATACTAGGTCAAAA

AGTGAGTTGAACTATGATGGATTGACTACTCAACCAAAAGAAGCTCCTGTAGAACCAAAG

ATTCAAACTCAGCCTAAACATGACGGATTGACTACTCCAGAGCTGCCAGAAAACAAGCGG

ATTCCTGTGCCGGATCACGTTGAACCGGAGCAGCCGAAAAGAGGCTTTTGGAGCCGTTTT

TTTAGACCCTATGATTAA

>GR58_CP051879

ATCCATGGCTCACAAGGCTTTCAGCGTTCTAACGATTTTCTACTTCAAGACCAATCTGCA

AAATTATTACCCAAAGAACGGGTCTGCAACTGCCTAAAAAAACGTATCGATAAAACTAAA

CAACGTGAAGTTAAATACAACGAAAATCGAAAAAAAGCTCATTATGCGAACGTTCAGCGT

TGTGGCTCTATATGGTCGTGTCCTGTTTGTGCCAAGCAAATTACCGAAAAACGTCGGGTA

GAGCTTAAAAAGGGCTTAGAAACGTGGAAAAACGTCCATAGAGGCTCAGTTATGCTACTG

ACACTTACCTTTAGCCATTCCCAGTCAGAATCCCTTAAATCGCTCTTAGAACGCCAAAGA

AAGGCATATAAAATTTTTCTTGAAACAACCAAAGTAAAAGAAATATTCAAACATTTTGGG

GTCAAATACAAAATTCGCAGTCTGGAAGCTACCTACGGCCAAAATGGTTGGCATCCGCAT

TTTCATGTACTTCTCTTAGGCTATTTTAAAATTGAAGATTTGATGTATCGAGATCTTCTT

GCGGAATTATGGATTAAGTCCTGTGTTCGAGCTGGTCTCAATGCTCCAAGCATGACCCAT

GGTTTAGATTTACGGGATGGAACCTATGCCGATCAGTATGTTTCAAAATGGGGAATTGAA

TCTGAATTAACGAAAGGTCATGTAAAGAAAGGTCGTAACGGAGGTTATACCCCGTTCGAC

CTGCTCCAGTTCTCTATGTATAACGAATCAGTTTTTGAAAAAGATTGCGGAAAATTATTC

CAAGAATTTGCCATTGCCATGAAAGGTTCACGCCAATTGGTATGGTCTCGAGGTCTTAAA

GCACTCTTAGAATTGGAAGAAAAAACTGATGAAGAATTGGCTGAAGAAACAGAAAAAGAC

GCTATTTCTTTACGGACCATTGATGATTTTATTTTTAGTTTACTTTGCCATTATCAAAAG

AGGTGGGATTTCCTTAGATGCCTAGAAAGAGACTATGAAAACGGCTGTTTTGGTACTGGA

GAAACCGAGCAATTACTTATAGATATTTTGGAAAAAGAACACATCAGATTAGGTATAGCG

AGTTAA

>GR59_CP090384

ATGACTGAATCTGACGAACAACAAGAACTAGAATACTTACCTTATTGTATTGGTAACATT

CGGCATAACGGGGTGGTCAGTACCAGTAACCGTTTAATTCGACCGATTGAACTGTCTACT

AATGAATATAAGGCTTTGCTGTATGCAATGGCAGTGGCTAATTATGGTGAAAAAAACAAC

CGAGATCGTGAAATCACCGAACAGACTTATATTTATCTGCATAAAGATGATCTTGGTGAA

TTACTGGGACTCAATAAGAAAAATTCTATTAATGTTGCTATCGATCGGATTTACAAAGAA

CTCTCATCTCGGGTTGCTCATTTTGTTATTGAAGAACCTGTAGATGATCTTAAGCGAAAG

GTAAAAAAAGTACATTCAGTAGTGCCAATAATCCGAGAATTACGCTGGGAAGATGATTTT

AAAAATGCTTTACAGATTCGTTTTACGAGTGAGGTCTTACCTTATTTTACTCGCCTTGCA

AATGGTAATTTCACCACTTACCAGCTAAAGGATCTGTTTGCTTTGGATTCTGTTACGAGT

ATGAGCCTGTATTCCTATATTGTGAGACAGGAATTTAAGTATGCTAACCTGGATACTTAT

GAAGTTGAACTATCGTTAGAAAGCTTGAAAGCACTGATAGACATTGGTGAAAAAAAATAT

GATCGTTGGGTCGATTTCAGACGTTATATTCTCGATAAAATCGTTGAGGAAATTAATACT

AAAACTAGTCTGAAATTAGAATATGACAGTATTAAAAAAGGACGGCCTGTAGTAGGCGTA

CGATTTAAAATTATAAATGGTCAAGAAGTGAATGCGATTTCACAATCAAATGAAAAAACT

AAAATTTATCTGGACGTAGATTTTAATGATAATAATTTGGTGAAAGAGCTAGGGGCAAAG

TTCGATATGACAGTGCGCTCTTGGTATATCTATGCTAACGATCCAAACTGTCATCAGTTA

GAAAGATGGTTTAAGCCAGAAGATTGTCTAACGGATTCGCAAGCCAACGTCATAGTAAAT

GACAATCTATTTCAAATGGAATTTGCTAAGCCAGGCTTGTCTATGACTGAGTTTAAAAAA

GAGATGAAAAATAAGCTGAAAAATGATCGAGACTTTGTTCAGACAAATCGTAATCGTCTA

AATGAAATTTTCGGGAAAGAAATAATTTAG

>GR60_CP084302_this_study

ATGTATGGTTATAAAATTGTAACCATGGAGCTTGGTTTGAAAAGTACAGAATTAATAAAA

CCTTCTTCTCGTCGTGTTCAATACACTGATTTAGTGGCTACACGTAATGATATGACAACG

GCAAATTACAGTTATGAAGCCAATGAAGAAAAGCTAGTTTACTGTGCTATGGTTGCTGTG

CGCAAAAATGAATTAAATAAAAATATGCGCTTTGATCCGGATGAACTTATTACAATTAGT

GCAGCTAATTTTGGAGAATTGATTTCGGAAAAACATTTAGATAAAGATGTTGTCACAGCC

AGTGAGTTATATGAAATTCATCGCTATGGTGAAAAGGCCTTACAGCGTGTTTACGATAGT

TATAAGCCTAAGGTGATGTTGATCAAGAAAAAAGATGATCCTACACCAATTAAAGTACCG

ATGATTATCTATTGTCATTATAGTAAAGAAACTAAATGCATGCAGATTCGTTTTGCTAGA

GAATTTTATAATTATTTTTATAATTTAATTAATCCAACAGGGATGACACATTCATTTAGT

ACACACCAAATTCGCTATGTTATGCGGATGCGTTCTAATTATGCTATGCGGATTTATCGA

ATTCTTAATTCTGAACTATGGAAGGCTGAGTCATTAGGTGTTCAGCAAATTCATGATATA

AGTTTGGAACGATTACGTTTTGCTCTAGATATTGAAGATAAATATAAACTGATCGATAAC

TTAAAAAGTCGTGTTTTGAATGTTGCAAAAACACAAATCAATAAGTTAAGTAATCTAGAA

GTTGATTATGAAACGATTAAAAATGGAAAATTTGTTGTGGGTATACGTTTTACTTACAAA

ATGAAAGATGAGCATCGTAATCTAATTTTTCAACGTATCATTGATCGTTTAAAAGAAAAA

CATCTTAAAAATGCAATTCCATATCGTGATGATGGATCGCACTTTAAAGATAAAGAACGT

ATTAAATATATTAAACCAGTCACTCGGTTGAGTTCGAAGCAAATCACAGTTTTAGTGAAT

TGTGATGTGTTTTTAAATGATTATGGTTATTTTTTAGGTAGTTTGGATACGATTACAGCT

AAAAAAATGATGCGCAGTTTATTAATAAATAAATTAGAGATATTGAATGATCATAAACCT

ATTGATTTAGACTACTATTTTTGGATACAAGCAAAACGTAATATGAATATTTTTAAAAAA

GATGCATCTGAAGATAATATGGCAGATCATGAAGATAAAAATATTGTTGATTCTGATATG

GATGAAATTTTAGATCCATCAGAAGATCAGTTACCGTTCTGGTAA

>GR61_CP048661_this_study

ATGTCTATTAATGGAATTTATTTCTTTAATCACAATATGGCAAATCTAATTTATAAAGAC

AATAACTTGATTGAAGCATCTTATGCATTGACTCTGTCTGAACAACGCTTAATTTTAGTT

GCAATTATTGCAGCTAGGGAAATTGAAAAAGAGCTTACATCGGATACATTGCTAACAATA

CATGCGTCTGAATACATGAAGCACTTTAATTTGGGTCGTCAAGCGGCCTATGAGGCTCTT

CAGGGAGCGTGTGATAACTTATTTGAACGTAGACTCACCTATAAGGCCATAGATCCTGTT

ACAGGTAAGCCAGCGGTCTACAAAAGCCGTTGGGTATCAAAAGTTGGCTATGTTAAAGAA

GCTGCATGTGCTCAACTAATTTTTGCTCCTGATATTTTACAGCTTTTTGTAAAACTTGAA

GAAAAGTTTACTCGTTATGAATTAAAGCAAATTTCTCAATTATCTAGTGTATACGCTATA

CGGCTGTATGAACTTTTGATTAGATGGCGTAGCAAAGGCAAGCTATATATTAGTATGGTG

GAATTGCGTGATAAGTTAGGTCTACTTGAAAATGAATATAAAACCATGGGAGATTTCAAA

AAGCGTGTATTGACGGTTGCCATAGATCAAATTAATAAACTTACTGATATCGACGTAAGT

TATGAGCAGAAAAAAGAAGGGAGAACAATTACACATATCGAATTTTCTTTTAATCAAAAA

GCTTCCTTAATCGTTAGTGATAAGGTACTGGAACCGACATATCAGCTGACACCAAAACAA

TCTATATTTTTCGCTCAAAAATTGTGTGACCTTATCAAATATCCCGAATTCGGGGGGAAA

TATGCCAATGTAGGCGAAGAGATCGAAGCGTTTAAAGAAAGGATTTCACTTGAACTCCTC

GACCCTGAAAAGGTAAAAAAGTATTATTCTGATTTGTTAAAAGTGGGCTATAAAGAAAAA

TATAAATCAGAAAAGTTATCATAA

>CP039146

ATGACAGAATCTGAACCATTGCAAGAAGAATCGAATTATATTCCTTACTGTATAGGCAAC

ATTCGCCATAATGGCGTTGTCAGTACCAGTAACCGTTTAATTCGCCCAATCGAATTATCA

GCCAATGAATATAAGGCACTTTTATATGCAATGGCGGTTGCGAATTATAGTGAGAAAAAT

AGGGTAGATGGTGAAATTACCGAACAAACTTATATTTATTTGTATAAGGATGATTTGGCA

GATTTACTAGGATTAAATAAACGTAATTCAATTAATGTTGCAATAGATCGGATTTATAAA

GAGCTTTCATCCCGGGTAGCTCATTTTATTATTGAGGAGCCAGCTGATAATGGTAAGAGA

AAGACTAAAAAAGTACATTCTGTCGTTCCCATTATCCGTGAGTTAAGATGGGAAGATGAT

TCTAAAAATGCGATTCAGATTCGTTTTACCAGTGAGGTGTTGCCGTATTTCACTCAACTG

GCTGGGGGTAATTTTACTACCTATCAGCTAAAACATCTTTTTGCCCTAGATTCTGTTGCC

AGTATGAGTCTTTATACTTATTTCATTAAAAACGAATTTAAGTACGTCAATCAAAAGAGT

TATGAAGTGCCATTACTTTTAGAAAACTTGAAAGCACTGATTGATATTAATGAAACCAAA

TATGATCGCTGGGTAGATTTTAGACGCTATGTATTAGACAAAATAGTGGCAGAAATTAAC

GAAAATACAGATCTACAGTTAGAGTATGAAACCGTTAAGAAAGGCCGTCCAATTGTAGGT

GTGAATTTTAAACTGCATCATCGGATTGCTGATAAGGCACTGGATGAATTTGCAGTGATT

GAAAAAATCTATCTTGATGTTCCATTTGAAGACAATGCATCCGTAAAAGAATTAGGGGCT

AAATTCGATATGAATGTACGTTCTTGGTATATTTTTAATAATGATGAAAATTATCAGCAG

TTCAAGAAGTGGTTTAAAAAAGTAGGATGCCTCACTGACTCTCAAGCAAATATTGTCATT

AATGACACTTTATTCCAAATGGATTTTGCTGAAATTGGTATGGGGTTGAATGACTTTAAA

CGAAATATGAAGCATAAACTTAAAAGTAATCCGGAATTCGTCCATAGTATTCGTGAAAGA

CTGAATGATATTTTTGGTAAAGAGTTGATCTAG

>CP044520

ATGACAGAATCTGAACCATTGCAAGAAGAATCGAATTATATTCCTTACTGTATAGGCAAC

ATTCGCCATAATGGCGTTGTCAGTACCAGTAACCGTTTAATTCGCCCAATCGAATTATCA

GCCAATGAATATAAGGCACTTTTATATGCAATGGCGGTTGCGAATTATAGTGAGAAAAAT

AGGGTAGATGGTGAAATTACCGAACAAACTTATATTTATTTGTATAAGGATGATTTGGCA

GATTTACTAGGATTAAATAAACGTAATTCAATTAATGTTGCAATAGATCGGATTTATAAA

GAGCTTTCATCCCGGGTAGCTCATTTTATTATTGAGGAGCCAGCTGATAATGGTAAGAGA

AAGACTAAAAAAGTACATTCTGTCGTTCCCATTATCCGTGAGTTAAGATGGGAAGATGAT

TCTAAAAATGCGATTCAGATTCGTTTTACCAGTGAGGTGTTGCCGTATTTCACTCAACTG

GCTGGGGGTAATTTTACTACCTATCAGCTAAAACATCTTTTTGCCCTAGATTCTGTTGCC

AGTATGAGTCTTTATACTTATTTCATTAAAAACGAATTTAAGTACGTCAATCAAAAGAGT

TATGAAGTGCCATTACTTTTAGAAAACTTGAAAGCACTGATTGATATTAATGAAACCAAA

TATGATCGCTGGGTAGATTTTAGACGCTATGTATTAGACAAAATAGTGGCAGAAATTAAC

GAAAATACAGATCTACAGTTAGAGTATGAAACCGTTAAGAAAGGCCGTCCAATTGTAGGT

GTGAATTTTAAACTGCATCATCGGATTGCTGATAAGGCACTGGATGAATTTGCAGTGATT

GAAAAAATCTATCTTGATGTTCCATTTGAAGACAATGCATCCGTAAAAGAATTAGGGGCT

AAATTCGATATGAATGTACGTTCTTGGTATATTTTTAATAATGATGAAAATTATCAGCAG

TTCAAGAAGTGGTTTAAAAAAGTAGGATGCCTCACTGACTCTCAAGCAAATATTGTCATT

AATGACACTTTATTCCAAATGGATTTTGCTGAAATTGGTATGGGGTTGAATGACTTTAAA

CGAAATATGAAGCATAAACTTAAAAGTAATCCGGAATTCGTCCATAGTATTCGTGAAAGA

CTGAATGATATTTTTGGTAAAGAGTTGATCTAG

>CP044457

ATGACAGAATCTGAACCATTGCAAGAAGAATCGAATTATATTCCTTACTGTATAGGCAAC

ATTCGCCATAATGGCGTTGTCAGTACCAGTAACCGTTTAATTCGCCCAATCGAATTATCA

GCCAATGAATATAAGGCACTTCTATATGCAATGGCGGTTGCGAATTATAGTGAGAAAAAT

AGGGTAGATGGTGAAATTACCGAACAAACTTATATTTATTTGTATAAGGATGATTTGGCA

GATTTACTAGGATTAAATAAACGTAATTCAATTAATGTTGCAATAGATCGGATCTACAAA

GAGCTTTCATCCCGGGTAGCTCATTTTATTATTGAGGAGCCTGCTGATAATGGTAAAAGA

AAGACTAAAAAAGTACATTCTGTCGTTCCCATTATCCGTGAGTTAAGATGGGAAGATGAT

TCTAAAAATGCGATTCAGATTCGTTTTACCAGTGAGGTGTTGCCGTATTTCACTCAACTG

GCTGGGGGTAATTTTACTACCTATCAGCTAAAACATCTTTTTGCCCTAGATTCTGTTGCC

AGTATGAGTCTCTATACTTATTTCATTAAAAATGAATTTAAGTACGCCAATCAAAAGAGT

TATGAAGTGCCATTACTTTTAGAAAACTTGAAAGCACTGATTGATATTAATGAAACCAAA

TATGATCGCTGGGTAGATTTTAGACGCTATGTATTAGACAAAATAGTGGCAGAAATTAAC

GAAAATACAGATCTACAATTAGAGTATGAAACCGTTAAGAAAGGCCGTCCAATTGTAGGT

GTGAATTTTAAACTGCATCATCGGATTGCTGATAAGGCACTGGATGAATTTGCAGTGATT

GAAAAAATCTATCTTGATGTTCCATTTGAAGACAATGCATCCGTAAAAGAATTAGGGGCT

AAATTCGATATGAATGTACGTTCTTGGTATATTTTTAATAATGATGAAAATTATCAGCAG

TTCAAGAAGTGGTTTAAAAAAGTAGGATGCCTCACTGACTCTCAAGCAAATATTGTCATT

AATGACACTTTATTCCAAATGGATTTTGCTGAAATTGGTATGGGGTTGAATGACTTTAAA

CGAAATATGAAGCATAAACTTAAAAGTAATCCGGAATTCGTCCATAGTATTCGTGAAAGA

CTGAATGATATTTTTGGTAAAGAGTTGATCTAG

>CP044446

ATGACAGAATCTGAACCATTGCAAGAAGAATCGAATTATATTCCTTACTGTATAGGCAAC

ATTCGCCATAATGGCGTTGTCAGTACCAGTAACCGTTTAATTCGCCCAATCGAATTATCA

GCCAATGAATATAAGGCACTTCTATATGCAATGGCGGTTGCGAATTATAGTGAGAAAAAT

AGGGTAGATGGTGAAATTACCGAACAAACTTATATTTATTTGTATAAGGATGATTTGGCA

GATTTACTAGGATTAAATAAACGTAATTCAATTAATGTTGCAATAGATCGGATCTACAAA

GAGCTTTCATCCCGGGTAGCTCATTTTATTATTGAGGAGCCTGCTGATAATGGTAAAAGA

AAGACTAAAAAAGTACATTCTGTCGTTCCCATTATCCGTGAGTTAAGATGGGAAGATGAT

TCTAAAAATGCGATTCAGATTCGTTTTACCAGTGAGGTGTTGCCGTATTTCACTCAACTG

GCTGGGGGTAATTTTACTACCTATCAGCTAAAACATCTTTTTGCCCTAGATTCTGTTGCC

AGTATGAGTCTCTATACTTATTTCATTAAAAATGAATTTAAGTACGCCAATCAAAAGAGT

TATGAAGTGCCATTACTTTTAGAAAACTTGAAAGCACTGATTGATATTAATGAAACCAAA

TATGATCGCTGGGTAGATTTTAGACGCTATGTATTAGACAAAATAGTGGCAGAAATTAAC

GAAAATACAGATCTACAATTAGAGTATGAAACCGTTAAGAAAGGCCGTCCAATTGTAGGT

GTGAATTTTAAACTGCATCATCGGATTGCTGATAAGGCACTGGATGAATTTGCAGTGATT

GAAAAAATCTATCTTGATGTTCCATTTGAAGACAATGCATCCGTAAAAGAATTAGGGGCT

AAATTCGATATGAATGTACGTTCTTGGTATATTTTTAATAATGATGAAAATTATCAGCAG

TTCAAGAAGTGGTTTAAAAAAGTAGGATGCCTCACTGACTCTCAAGCAAATATTGTCATT

AATGACACTTTATTCCAAATGGATTTTGCTGAAATTGGTATGGGGTTGAATGACTTTAAA

CGAAATATGAAGCATAAACTTAAAAGTAATCCGGAATTCGTCCATAGTATTCGTGAAAGA

CTGAATGATATTTTTGGTAAAGAGTTGATCTAG

>CP040912

ATGACAGAATCTGAACCATTGCAAGAAGAATCGAATTATATTCCTTACTGTATAGGCAAC

ATTCGCCATAATGGCGTTGTCAGTACCAGTAACCGTTTAATTCGCCCAATCGAATTATCA

GCCAATGAATATAAGGCACTTTTATATGCAATGGCGGTTGCGAATTATAGTGAGAAAAAT

AGGGTAGATGGTGAAATTACCGAACAAACTTATATTTATTTGTATAAGGATGATTTGGCA

GATTTACTAGGATTAAATAAACGTAATTCAATTAATGTTGCAATAGATCGGATTTATAAA

GAGCTTTCATCCCGGGTAGCTCATTTTATTATTGAGGAGCCAGCTGATAATGGTAAGAGA

AAGACTAAAAAAGTACATTCTGTCGTTCCCATTATCCGTGAGTTAAGATGGGAAGATGAT

TCTAAAAATGCGATTCAGATTCGTTTTACCAGTGAGGTGTTGCCGTATTTCACTCAACTG

GCTGGGGGTAATTTTACTACCTATCAGCTAAAACATCTTTTTGCCCTAGATTCTGTTGCC

AGTATGAGTCTTTATACTTATTTCATTAAAAACGAATTTAAGTACGTCAATCAAAAGAGT

TATGAAGTGCCATTACTTTTAGAAAACTTGAAAGCACTGATTGATATTAATGAAACCAAA

TATGATCGCTGGGTAGATTTTAGACGCTATGTATTAGACAAAATAGTGGCAGAAATTAAC

GAAAATACAGATCTACAGTTAGAGTATGAAACCGTTAAGAAAGGCCGTCCAATTGTAGGT

GTGAATTTTAAACTGCATCATCGGATTGCTGATAAGGCACTGGATGAATTTGCAGTGATT

GAAAAAATCTATCTTGATGTTCCATTTGAAGACAATGCATCCGTAAAAGAATTAGGGGCT

AAATTCGATATGAATGTACGTTCTTGGTATATTTTTAATAATGATGAAAATTATCAGCAG

TTCAAGAAGTGGTTTAAAAAAGTAGGATGCCTCACTGACTCTCAAGCAAATATTGTCATT

AATGACACTTTATTCCAAATGGATTTTGCTGAAATTGGTATGGGGTTGAATGACTTTAAA

CGAAATATGAAGCATAAACTTAAAAGTAATCCGGAATTCGTCCATAGTATTCGTGAAAGA

CTGAATGATATTTTTGGTAAAGAGTTGATCTAG

>CP044019

ATGACAGAATCTGAACCATTGCAAGAAGAATCGAATTATATACCTTACTGTATAGGCAAC

ATTCGCCATAATGGCGTTGTCAGTACCAGTAACCGTTTAATTCGCCCAATCGAATTATCA

GCCAATGAATATAAGGCACTTCTATATGCAATGGCGGTTGCGAATTATAGTGAGAAAAAT

AGGGTAGATGGTGAAATTACCGAACAAACTTATATTTATTTGTATAAGGATGATTTGGCA

GATTTACTAGGATTAAATAAACGTAATTCAATTAATGTTGCAATAGATCGGATCTATAAA

GAGCTTTCATCCCGGGTAGCTCATTTTATTATTGAGGAGCCAGCTGATAATGGTAAGAGA

AAGACTAAAAAAGTACATTCTGTCGTTCCCATTATCCGTGAGTTAAGATGGGAAGATGAT

TCTAAAAATGCGATTCAGATTCGTTTTACCAGTGAGGTGTTGCCGTATTTCACTCAACTG

GCTGGGGGTAATTTTACTACCTATCAGCTAAAACATCTTTTTGCCCTAGATTCTGTTGCC

AGTATGAGTCTTTATACTTATTTCATTAAAAACGAATTTAAGTACGCCAATCAAAAGAGT

TATGAAGTGCCATTACTTTTAGAAAACTTGAAAGCACTGATTGATATTAATGAAACCAAA

TATGATCGCTGGGTAGATTTTAGACGCTATGTATTAGACAAAATAGTGGCAGAAATTAAC

GAAAATACAGATTTACAGTTAGAGTATGAAACCGTTAAGAAAGGCCGTCCAATTGTAGGT

GTGAATTTTAAACTGCATCATCGGATTGCTGATAAGGCACTGGATGAATTTGCAGTGATT

GAAAAAATCTATCTTGATGTTCCATTTGAAGACAATGCATACGTAAAAGAATTAGGGGCT

AAATTCGATATGAATGTACGTTCTTGGTATATTTTTAATAATGATGAAAATTATCAGCAG

TTCAAGAAGTGGTTTAAAAAAGTAGGATGCCTCACTGACTCTCAAGCAAATATTGTCATT

AATGACACTTTATTCCAAATGGATTTTGCTGAAATTGGTATGGGGTTGAATGAATTTAAA

CGAAATATGAAGCATAAACTTAAAAGTAATCCGGAATTCGTCCATAGTATTCGTGAAAGA

CTGAATGATATTTTTGGTAAAGAGTTGATCTAG

>CP045132

ATGACAGAATCTGAACCATTGCAAGAAGAATCGAATTATATTCCTTACTGTATAGGCAAC

ATTCGCCATAATGGCGTTGTCAGTACCAGTAACCGTTTAATTCGCCCAATCGAATTATCA

GCCAATGAATATAAGGCACTTTTATATGCAATGGCGGTTGCGAATTATAGTGAGAAAAAT

AGGGTAGATGGTGAAATTACCGAACAAACTTATATTTATTTGTATAAGGATGATTTGGCA

GATTTACTAGGATTAAATAAACGTAATTCAATTAATGTTGCAATAGATCGGATTTATAAA

GAGCTTTCATCCCGGGTAGCTCATTTTATTATTGAGGAGCCAGCTGATAATGGTAAGAGA

AAGACTAAAAAAGTACATTCTGTCGTTCCCATTATCCGTGAGTTAAGATGGGAAGATGAT

TCTAAAAATGCGATTCAGATTCGTTTTACCAGTGAGGTGTTGCCGTATTTCACTCAACTG

GCTGGGGGTAATTTTACTACCTATCAGCTAAAACATCTTTTTGCCCTAGATTCTGTTGCC

AGTATGAGTCTTTATACTTATTTCATTAAAAACGAATTTAAGTACGTCAATCAAAAGAGT

TATGAAGTGCCATTACTTTTAGAAAACTTGAAAGCACTGATTGATATTAATGAAACCAAA

TATGATCGCTGGGTAGATTTTAGACGCTATGTATTAGACAAAATAGTGGCAGAAATTAAC

GAAAATACAGATCTACAGTTAGAGTATGAAACCGTTAAGAAAGGCCGTCCAATTGTAGGT

GTGAATTTTAAACTGCATCATCGGATTGCTGATAAGGCACTGGATGAATTTGCAGTGATT

GAAAAAATCTATCTTGATGTTCCATTTGAAGACAATGCATCCGTAAAAGAATTAGGGGCT

AAATTCGATATGAATGTACGTTCTTGGTATATTTTTAATAATGATGAAAATTATCAGCAG

TTCAAGAAGTGGTTTAAAAAAGTAGGATGCCTCACTGACTCTCAAGCAAATATTGTCATT

AATGACACTTTATTCCAAATGGATTTTGCTGAAATTGGTATGGGGTTGAATGACTTTAAA

CGAAATATGAAGCATAAACTTAAAAGTAATCCGGAATTCGTCCATAGTATTCGTGAAAGA

CTGAATGATATTTTTGGTAAAGAGTTGATCTAG

>CP048015

ATGACAGAATCAGAATCATTGCAAGAAGAATTGAATCATATTCCTTACTGTATAGGCAAT

ATTCGTCATAATGGCGTTGTTAGCACTAGTAACCGCTTAATTCGCCCAATTGAGTTATCA

GCCAATGAATATAAGGCGCTCCTATATGCAATGGCTGTTGCAAATTATAGTGAGAAAAAT

AGGGTAAATGGTGAAATCACAGAACAAACCTATATTTATCTGTATAAGGATGATTTGGCA

GATTTACTAGGATTGAATAAACGCAATTCGATTAATGTTGCAATAGACCGGATTTATAAA

GAACTTTCATCTAGGGTGGCTCATTTTATTATTGAGGAACCTACTGATGATGGTAAGAGA

AAGACTAAAAAAGTACATTCTGTCGTTCCTATTATCCGTGAGTTAAGATGGGAAGATGAT

TCTAAAAATGCCATTCAGATTCGTTTTACCAGTGAGGTGTTACCGTATTTCACGCAGTTG

GCTGGGGGGAATTTCACCACCTATCAATTGAAACATCTGTTTGCCTTGGATTCTGTTGCT

AGTATGAGTCTTTATACGTATTTCATTAAAAATGAATTCAAGTACGCAAGCCAAAAGAGT

TATGAAGTCCCAATGCTTTTAGAAAACTTGAAAGCTCTGATTGATATTAATGAAACTAAA

TATGATCGCTGGGTAGATTTTAGACGCTATGTATTAGATAAAATAGTTGCAGAAATTAAC

GAAAATACAGATCTACAACTAGAGTATGAAACCATAAAAAAAGGGCGCCCAATTGTGGGG

GTGAATTTTAAACTGCATCAGCGTATTACTGATAAAACACTAAATGAAACTGCTGTAATT

GAAAAAATTTATCTTGATGTTCCATTTGAAGAGAATGCATTAGTAAAAGAATTGGGGGCA

AAATTCGATACGAATGTACGTTCCTGGTACATTTTTAATAATGATGAAAAGTATTCGCAG

TTCAAGAAGTGGTTTAAAAAAATTGGTTGTCTCACTGACTCTCAAGCAAATATTGTTATT

AATGACACTTTATTCCAAATGGATTTTGCTGAAGTTGGTATGAGCTTGAATGACTTTAAA

CGTAATATGAAGCATAAACTAAAAAATAATCCGGAATTTGTACAAAGTATTCGTGATAGG

CTGAATGATATTTTTGGTAAAGAGCTTGTCTAA

>CP071767

ATGACAGAATCAGAATCATTGCAAGAAGAATTGAATCATATTCCTTACTGTATAGGCAAT

ATTCGTCATAATGGCGTTGTTAGCACTAGTAACCGCTTAATTCGCCCAATTGAGTTATCA

GCCAATGAATATAAGGCGCTCCTATATGCAATGGCTGTTGCAAATTATAGTGAGAAAAAT

AGGGTAAATGGTGAAATCACAGAACAAACCTATATTTATCTGTATAAGGATGATTTGGCA

GATTTACTAGGATTGAATAAACGCAATTCGATTAATGTTGCAATAGACCGGATTTATAAA

GAACTTTCATCTAGGGTGGCTCATTTTATTATTGAGGAACCTACTGATGATGGTAAGAGA

AAGACTAAAAAAGTACATTCTGTCGTTCCTATTATCCGTGAGTTAAGATGGGAAGATGAT

TCTAAAAATGCCATTCAGATTCGTTTTACCAGTGAGGTGTTACCGTATTTCACGCAGTTG

GCTGGGGGGAATTTCACCACCTATCAATTGAAACATCTGTTTGCCTTGGATTCTGTTGCT

AGTATGAGTCTTTATACGTATTTCATTAAAAATGAATTCAAGTACGCAAGCCAAAAGAGT

TATGAAGTCCCAATGCTTTTAGAAAACTTGAAAGCTCTGATTGATATTAATGAAACTAAA

TATGATCGCTGGGTAGATTTTAGACGCTATGTATTAGATAAAATAGTTGCAGAAATTAAC

GAAAATACAGATCTACAACTAGAGTATGAAACCATAAAAAAAGGGCGCCCAATTGTGGGG

GTGAATTTTAAACTGCATCAGCGTATTACTGATAAAACACTAAATGAAACTGCTGTAATT

GAAAAAATTTATCTTGATGTTCCATTTGAAGAGAATGCATTAGTAAAAGAATTGGGGGCA

AAATTCGATACGAATGTACGTTCCTGGTACATTTTTAATAATGATGAAAAGTATTCGCAG

TTCAAGAAGTGGTTTAAAAAAATTGGTTGTCTCACTGACTCTCAAGCAAATATTGTTATT

AATGACACTTTATTCCAAATGGATTTTGCTGAAGTTGGTATGAGCTTGAATGACTTTAAA

CGTAATATGAAGCATAAACTAAAAAATAATCCGGAATTTGTACAAAGTATTCGTGATAGG

CTGAATGATATTTTTGGTAAAGAGCTTGTCTAA

>CP071769

ATGACAGAATCAGAATCATTGCAAGAAGAATTGAATCATATTCCTTACTGTATAGGCAAT

ATTCGTCATAATGGCGTTGTTAGCACTAGTAACCGCTTAATTCGCCCAATTGAGTTATCA

GCCAATGAATATAAGGCGCTCCTATATGCAATGGCTGTTGCAAATTATAGTGAGAAAAAT

AGGGTAAATGGTGAAATCACAGAACAAACCTATATTTATCTGTATAAGGATGATTTGGCA

GATTTACTAGGATTGAATAAACGCAATTCGATTAATGTTGCAATAGACCGGATTTATAAA

GAACTTTCATCTAGGGTGGCTCATTTTATTATTGAGGAACCTACTGATGATGGTAAGAGA

AAGACTAAAAAAGTACATTCTGTCGTTCCTATTATCCGTGAGTTAAGATGGGAAGATGAT

TCTAAAAATGCCATTCAGATTCGTTTTACCAGTGAGGTGTTACCGTATTTCACGCAGTTG

GCTGGGGGGAATTTCACCACCTATCAATTGAAACATCTGTTTGCCTTGGATTCTGTTGCT

AGTATGAGTCTTTATACGTATTTCATTAAAAATGAATTCAAGTACGCAAGCCAAAAGAGT

TATGAAGTCCCAATGCTTTTAGAAAACTTGAAAGCTCTGATTGATATTAATGAAACTAAA

TATGATCGCTGGGTAGATTTTAGACGCTATGTATTAGATAAAATAGTTGCAGAAATTAAC

GAAAATACAGATCTACAACTAGAGTATGAAACCATAAAAAAAGGGCGCCCAATTGTGGGG

GTGAATTTTAAACTGCATCAGCGTATTACTGATAAAACACTAAATGAAACTGCTGTAATT

GAAAAAATTTATCTTGATGTTCCATTTGAAGAGAATGCATTAGTAAAAGAATTGGGGGCA

AAATTCGATACGAATGTACGTTCCTGGTACATTTTTAATAATGATGAAAAGTATTCGCAG

TTCAAGAAGTGGTTTAAAAAAATTGGTTGTCTCACTGACTCTCAAGCAAATATTGTTATT

AATGACACTTTATTCCAAATGGATTTTGCTGAAGTTGGTATGAGCTTGAATGACTTTAAA

CGTAATATGAAGCATAAACTAAAAAATAATCCGGAATTTGTACAAAGTATTCGTGATAGG

CTGAATGATATTTTTGGTAAAGAGCTTGTCTAA

>CP071772

ATGACAGAATCAGAATCATTGCAAGAAGAATTGAATCATATTCCTTACTGTATAGGCAAT

ATTCGTCATAATGGCGTTGTTAGCACTAGTAACCGCTTAATTCGCCCAATTGAGTTATCA

GCCAATGAATATAAGGCGCTCCTATATGCAATGGCTGTTGCAAATTATAGTGAGAAAAAT

AGGGTAAATGGTGAAATCACAGAACAAACCTATATTTATCTGTATAAGGATGATTTGGCA

GATTTACTAGGATTGAATAAACGCAATTCGATTAATGTTGCAATAGACCGGATTTATAAA

GAACTTTCATCTAGGGTGGCTCATTTTATTATTGAGGAACCTACTGATGATGGTAAGAGA

AAGACTAAAAAAGTACATTCTGTCGTTCCTATTATCCGTGAGTTAAGATGGGAAGATGAT

TCTAAAAATGCCATTCAGATTCGTTTTACCAGTGAGGTGTTACCGTATTTCACGCAGTTG

GCTGGGGGGAATTTCACCACCTATCAATTGAAACATCTGTTTGCCTTGGATTCTGTTGCT

AGTATGAGTCTTTATACGTATTTCATTAAAAATGAATTCAAGTACGCAAGCCAAAAGAGT

TATGAAGTCCCAATGCTTTTAGAAAACTTGAAAGCTCTGATTGATATTAATGAAACTAAA

TATGATCGCTGGGTAGATTTTAGACGCTATGTATTAGATAAAATAGTTGCAGAAATTAAC

GAAAATACAGATCTACAACTAGAGTATGAAACCATAAAAAAAGGGCGCCCAATTGTGGGG

GTGAATTTTAAACTGCATCAGCGTATTACTGATAAAACACTAAATGAAACTGCTGTAATT

GAAAAAATTTATCTTGATGTTCCATTTGAAGAGAATGCATTAGTAAAAGAATTGGGGGCA

AAATTCGATACGAATGTACGTTCCTGGTACATTTTTAATAATGATGAAAAGTATTCGCAG

TTCAAGAAGTGGTTTAAAAAAATTGGTTGTCTCACTGACTCTCAAGCAAATATTGTTATT

AATGACACTTTATTCCAAATGGATTTTGCTGAAGTTGGTATGAGCTTGAATGACTTTAAA

CGTAATATGAAGCATAAACTAAAAAATAATCCGGAATTTGTACAAAGTATTCGTGATAGG

CTGAATGATATTTTTGGTAAAGAGCTTGTCTAA

>CP046596

ATGACAGAATCTGAACCATTGCAAGAAGAATCGAATTATATTCCTTACTGTATAGGCAAC

ATTCGCCATAATGGCGTTGTCAGTACCAGTAACCGTTTAATTCGCCCAATCGAATTATCA

GCCAATGAATATAAGGCACTTCTATATGCAATGGCGGTTGCGAATTATAGTGAGAAAAAT

AGGGTAGATGGTGAAATTACCGAACAAACTTATATTTATTTGTATAAGGATGATTTGGCA

GATTTACTAGGATTAAATAAACGTAATTCAATTAATGTTGCAATAGATCGGATCTACAAA

GAGCTTTCATCCCGGGTAGCTCATTTTATTATTGAGGAGCCTGCTGATAATGGTAAAAGA

AAGACTAAAAAAGTACATTCTGTCGTTCCCATTATCCGTGAGTTAAGATGGGAAGATGAT

TCTAAAAATGCGATTCAGATTCGTTTTACCAGTGAGGTGTTGCCGTATTTCACTCAACTG

GCTGGGGGTAATTTTACTACCTATCAGCTAAAACATCTTTTTGCCCTAGATTCTGTTGCC

AGTATGAGTCTCTATACTTATTTCATTAAAAATGAATTTAAGTACGCCAATCAAAAGAGT

TATGAAGTGCCATTACTTTTAGAAAACTTGAAAGCACTGATTGATATTAATGAAACCAAA

TATGATCGCTGGGTAGATTTTAGACGCTATGTATTAGACAAAATAGTGGCAGAAATTAAC

GAAAATACAGATCTACAATTAGAGTATGAAACCGTTAAGAAAGGCCGTCCAATTGTAGGT

GTGAATTTTAAACTGCATCATCGGATTGCTGATAAGGCACTGGATGAATTTGCAGTGATT

GAAAAAATCTATCTTGATGTTCCATTTGAAGACAATGCATCCGTAAAAGAATTAGGGGCT

AAATTCGATATGAATGTACGTTCTTGGTATATTTTTAATAATGATGAAAATTATCAGCAG

TTCAAGAAGTGGTTTAAAAAAGTAGGATGCCTCACTGACTCTCAAGCAAATATTGTCATT

AATGACACTTTATTCCAAATGGATTTTGCTGAAATTGGTATGGGGTTGAATGACTTTAAA

CGAAATATGAAGCATAAACTTAAAAGTAATCCGGAATTCGTCCATAGTATTCGTGAAAGA

CTGAATGATATTTTTTGGTAA

>CP094546

ATGACAGAATCTGAACCATTGCAAGAAGAATCGAATTATATTCCTTACTGTATAGGCAAC

ATTCGCCATAATGGCGTTGTCAGTACCAGTAACCGTTTAATTCGCCCAATCGAATTATCA

GCCAATGAATATAAGGCACTTTTATATGCAATGGCGGTTGCGAATTATAGTGAGAAAAAT

AGGGTAGATGGTGAAATTACCGAACAAACTTATATTTATTTGTATAAGGATGATTTGGCA

GATTTACTAGGATTAAATAAACGTAATTCAATTAATGTTGCAATAGATCGGATTTATAAA

GAGCTTTCATCCCGGGTAGCTCATTTTATTATTGAGGAGCCAGCTGATAATGGTAAGAGA

AAGACTAAAAAAGTACATTCTGTCGTTCCCATTATCCGTGAGTTAAGATGGGAAGATGAT

TCTAAAAATGCGATTCAGATTCGTTTTACCAGTGAGGTGTTGCCGTATTTCACTCAACTG

GCTGGGGGTAATTTTACTACCTATCAGCTAAAACATCTTTTTGCCCTAGATTCTGTTGCC

AGTATGAGTCTTTATACTTATTTCATTAAAAACGAATTTAAGTACGCCAATCAAAAGAGT

TATGAAGTGCCATTACTTTTAGAAAACTTGAAAGCACTGATTGATATTAATGAAACCAAA

TATGATCGCTGGGTAGATTTTAGACGCTATGTATTAGACAAAATAGTGGCAGAAATTAAC

GAAAATACAGATCTACAGTTAGAGTATGAAACCGTTAAGAAAGGCCGTCCAATTGTAGGT

GTGAATTTTAAACTGCATCATCGGATTGCTGATAAGGCACTGGATGAATTTGCAGTGATT

GAAAAAATCTATCTTGATGTTCCATTTGAAGACAATGCATCCGTAAAAGAATTAGGGGCT

AAATTCGATATGAATGTACGTTCTTGGTATATTTTTAATAATGATGAAAATTATCAGCAG

TTCAAGAAGTGGTTTAAAAAAGTAGGATGCCTCACTGACTCTCAAGCAAATATTGTCATT

AATGACACTTTATTCCAAATGGATTTTGCTGAAATTGGTATGGGGTTGAATGACTTTAAA

CGAAATATGAAGCATAAACTTAAAAGTAATCCGGAATTCGTCCATAGTATTCGTGAAAGA

CTGAATGATATTTTTGGTAAAGAGTTGATCTAG

>CP044464

ATGACAGAATCTGAACCATTGCAAGAAGAATCGAATTATATTCCTTACTGTATAGGCAAC

ATTCGCCATAATGGCGTTGTCAGTACCAGTAACCGTTTAATTCGCCCAATCGAATTATCA

GCCAATGAATATAAGGCACTTCTATATGCAATGGCGGTTGCGAATTATAGTGAGAAAAAT

AGGGTAGATGGTGAAATTACCGAACAAACTTATATTTATTTGTATAAGGATGATTTGGCA

GATTTACTAGGATTAAATAAACGTAATTCAATTAATGTTGCAATAGATCGGATCTACAAA

GAGCTTTCATCCCGGGTAGCTCATTTTATTATTGAGGAGCCTGCTGATAATGGTAAAAGA

AAGACTAAAAAAGTACATTCTGTCGTTCCCATTATCCGTGAGTTAAGATGGGAAGATGAT

TCTAAAAATGCGATTCAGATTCGTTTTACCAGTGAGGTGTTGCCGTATTTCACTCAACTG

GCTGGGGGTAATTTTACTACCTATCAGCTAAAACATCTTTTTGCCCTAGATTCTGTTGCC

AGTATGAGTCTCTATACTTATTTCATTAAAAATGAATTTAAGTACGCCAATCAAAAGAGT

TATGAAGTGCCATTACTTTTAGAAAACTTGAAAGCACTGATTGATATTAATGAAACCAAA

TATGATCGCTGGGTAGATTTTAGACGCTATGTATTAGACAAAATAGTGGCAGAAATTAAC

GAAAATACAGATCTACAATTAGAGTATGAAACCGTTAAGAAAGGCCGTCCAATTGTAGGT

GTGAATTTTAAACTGCATCATCGGATTGCTGATAAGGCACTGGATGAATTTGCAGTGATT

GAAAAAATCTATCTTGATGTTCCATTTGAAGACAATGCATCCGTAAAAGAATTAGGGGCT

AAATTCGATATGAATGTACGTTCTTGGTATATTTTTAATAATGATGAAAATTATCAGCAG

TTCAAGAAGTGGTTTAAAAAAGTAGGATGCCTCACTGACTCTCAAGCAAATATTGTCATT

AATGACACTTTATTCCAAATGGATTTTGCTGAAATTGGTATGGGGTTGAATGACTTTAAA

CGAAATATGAAGCATAAACTTAAAAGTAATCCGGAATTCGTCCATAGTATTCGTGAAAGA

CTGAATGATATTTTTGGTAAAGAGTTGATCTAG

>CP044484

ATGACAGAATCTGAACCATTGCAAGAAGAATCGAATTATATTCCTTACTGTATAGGCAAC

ATTCGCCATAATGGCGTTGTCAGTACCAGTAACCGTTTAATTCGCCCAATCGAATTATCA

GCCAATGAATATAAGGCACTTCTATATGCAATGGCGGTTGCGAATTATAGTGAGAAAAAT

AGGGTAGATGGTGAAATTACCGAACAAACTTATATTTATTTGTATAAGGATGATTTGGCA

GATTTACTAGGATTAAATAAACGTAATTCAATTAATGTTGCAATAGATCGGATCTACAAA

GAGCTTTCATCCCGGGTAGCTCATTTTATTATTGAGGAGCCTGCTGATAATGGTAAAAGA

AAGACTAAAAAAGTACATTCTGTCGTTCCCATTATCCGTGAGTTAAGATGGGAAGATGAT

TCTAAAAATGCGATTCAGATTCGTTTTACCAGTGAGGTGTTGCCGTATTTCACTCAACTG

GCTGGGGGTAATTTTACTACCTATCAGCTAAAACATCTTTTTGCCCTAGATTCTGTTGCC

AGTATGAGTCTCTATACTTATTTCATTAAAAATGAATTTAAGTACGCCAATCAAAAGAGT

TATGAAGTGCCATTACTTTTAGAAAACTTGAAAGCACTGATTGATATTAATGAAACCAAA

TATGATCGCTGGGTAGATTTTAGACGCTATGTATTAGACAAAATAGTGGCAGAAATTAAC

GAAAATACAGATCTACAATTAGAGTATGAAACCGTTAAGAAAGGCCGTCCAATTGTAGGT

GTGAATTTTAAACTGCATCATCGGATTGCTGATAAGGCACTGGATGAATTTGCAGTGATT

GAAAAAATCTATCTTGATGTTCCATTTGAAGACAATGCATCCGTAAAAGAATTAGGGGCT

AAATTCGATATGAATGTACGTTCTTGGTATATTTTTAATAATGATGAAAATTATCAGCAG

TTCAAGAAGTGGTTTAAAAAAGTAGGATGCCTCACTGACTCTCAAGCAAATATTGTCATT

AATGACACTTTATTCCAAATGGATTTTGCTGAAATTGGTATGGGGTTGAATGACTTTAAA

CGAAATATGAAGCATAAACTTAAAAGTAATCCGGAATTCGTCCATAGTATTCGTGAAAGA

CTGAATGATATTTTTGGTAAAGAGTTGATCTAG

>CP044475

ATGACAGAATCTGAACCATTGCAAGAAGAATCGAATTATATTCCTTACTGTATAGGCAAC

ATTCGCCATAATGGCGTTGTCAGTACCAGTAACCGTTTAATTCGCCCAATCGAATTATCA

GCCAATGAATATAAGGCACTTCTATATGCAATGGCGGTTGCGAATTATAGTGAGAAAAAT

AGGGTAGATGGTGAAATTACCGAACAAACTTATATTTATTTGTATAAGGATGATTTGGCA

GATTTACTAGGATTAAATAAACGTAATTCAATTAATGTTGCAATAGATCGGATCTACAAA

GAGCTTTCATCCCGGGTAGCTCATTTTATTATTGAGGAGCCTGCTGATAATGGTAAAAGA

AAGACTAAAAAAGTACATTCTGTCGTTCCCATTATCCGTGAGTTAAGATGGGAAGATGAT

TCTAAAAATGCGATTCAGATTCGTTTTACCAGTGAGGTGTTGCCGTATTTCACTCAACTG

GCTGGGGGTAATTTTACTACCTATCAGCTAAAACATCTTTTTGCCCTAGATTCTGTTGCC

AGTATGAGTCTCTATACTTATTTCATTAAAAATGAATTTAAGTACGCCAATCAAAAGAGT

TATGAAGTGCCATTACTTTTAGAAAACTTGAAAGCACTGATTGATATTAATGAAACCAAA

TATGATCGCTGGGTAGATTTTAGACGCTATGTATTAGACAAAATAGTGGCAGAAATTAAC

GAAAATACAGATCTACAATTAGAGTATGAAACCGTTAAGAAAGGCCGTCCAATTGTAGGT

GTGAATTTTAAACTGCATCATCGGATTGCTGATAAGGCACTGGATGAATTTGCAGTGATT

GAAAAAATCTATCTTGATGTTCCATTTGAAGACAATGCATCCGTAAAAGAATTAGGGGCT

AAATTCGATATGAATGTACGTTCTTGGTATATTTTTAATAATGATGAAAATTATCAGCAG

TTCAAGAAGTGGTTTAAAAAAGTAGGATGCCTCACTGACTCTCAAGCAAATATTGTCATT

AATGACACTTTATTCCAAATGGATTTTGCTGAAATTGGTATGGGGTTGAATGACTTTAAA

CGAAATATGAAGCATAAACTTAAAAGTAATCCGGAATTCGTCCATAGTATTCGTGAAAGA

CTGAATGATATTTTTGGTAAAGAGTTGATCTAG

>CP094542

ATGACAGAATCTGAACCATTGCAAGAAGAATCGAATTATATTCCTTACTGTATAGGCAAC

ATTCGCCATAATGGCGTTGTCAGTACCAGTAACCGTTTAATTCGCCCAATCGAATTATCA

GCCAATGAATATAAGGCACTTTTATATGCAATGGCGGTTGCGAATTATAGTGAGAAAAAT

AGGGTAGATGGTGAAATTACCGAACAAACTTATATTTATTTGTATAAGGATGATTTGGCA

GATTTACTAGGATTAAATAAACGTAATTCAATTAATGTTGCAATAGATCGGATTTATAAA

GAGCTTTCATCCCGGGTAGCTCATTTTATTATTGAGGAGCCAGCTGATAATGGTAAGAGA

AAGACTAAAAAAGTACATTCTGTCGTTCCCATTATCCGTGAGTTAAGATGGGAAGATGAT

TCTAAAAATGCGATTCAGATTCGTTTTACCAGTGAGGTGTTGCCGTATTTCACTCAACTG

GCTGGGGGTAATTTTACTACCTATCAGCTAAAACATCTTTTTGCCCTAGATTCTGTTGCC

AGTATGAGTCTTTATACTTATTTCATTAAAAACGAATTTAAGTACGCCAATCAAAAGAGT

TATGAAGTGCCATTACTTTTAGAAAACTTGAAAGCACTGATTGATATTAATGAAACCAAA

TATGATCGCTGGGTAGATTTTAGACGCTATGTATTAGACAAAATAGTGGCAGAAATTAAC

GAAAATACAGATCTACAGTTAGAGTATGAAACCGTTAAGAAAGGCCGTCCAATTGTAGGT

GTGAATTTTAAACTGCATCATCGGATTGCTGATAAGGCACTGGATGAATTTGCAGTGATT

GAAAAAATCTATCTTGATGTTCCATTTGAAGACAATGCATCCGTAAAAGAATTAGGGGCT

AAATTCGATATGAATGTACGTTCTTGGTATATTTTTAATAATGATGAAAATTATCAGCAG

TTCAAGAAGTGGTTTAAAAAAGTAGGATGCCTCACTGACTCTCAAGCAAATATTGTCATT

AATGACACTTTATTCCAAATGGATTTTGCTGAAATTGGTATGGGGTTGAATGACTTTAAA

CGAAATATGAAGCATAAACTTAAAAGTAATCCGGAATTCGTCCATAGTATTCGTGAAAGA

CTGAATGATATTTTTGGTAAAGAGTTGATCTAG

>CP044451

ATGACAGAATCTGAACCATTGCAAGAAGAATCGAATTATATTCCTTACTGTATAGGCAAC

ATTCGCCATAATGGCGTTGTCAGTACCAGTAACCGTTTAATTCGCCCAATCGAATTATCA

GCCAATGAATATAAGGCACTTCTATATGCAATGGCGGTTGCGAATTATAGTGAGAAAAAT

AGGGTAGATGGTGAAATTACCGAACAAACTTATATTTATTTGTATAAGGATGATTTGGCA

GATTTACTAGGATTAAATAAACGTAATTCAATTAATGTTGCAATAGATCGGATCTACAAA

GAGCTTTCATCCCGGGTAGCTCATTTTATTATTGAGGAGCCTGCTGATAATGGTAAAAGA

AAGACTAAAAAAGTACATTCTGTCGTTCCCATTATCCGTGAGTTAAGATGGGAAGATGAT

TCTAAAAATGCGATTCAGATTCGTTTTACCAGTGAGGTGTTGCCGTATTTCACTCAACTG

GCTGGGGGTAATTTTACTACCTATCAGCTAAAACATCTTTTTGCCCTAGATTCTGTTGCC

AGTATGAGTCTCTATACTTATTTCATTAAAAATGAATTTAAGTACGCCAATCAAAAGAGT

TATGAAGTGCCATTACTTTTAGAAAACTTGAAAGCACTGATTGATATTAATGAAACCAAA

TATGATCGCTGGGTAGATTTTAGACGCTATGTATTAGACAAAATAGTGGCAGAAATTAAC

GAAAATACAGATCTACAATTAGAGTATGAAACCGTTAAGAAAGGCCGTCCAATTGTAGGT

GTGAATTTTAAACTGCATCATCGGATTGCTGATAAGGCACTGGATGAATTTGCAGTGATT

GAAAAAATCTATCTTGATGTTCCATTTGAAGACAATGCATCCGTAAAAGAATTAGGGGCT

AAATTCGATATGAATGTACGTTCTTGGTATATTTTTAATAATGATGAAAATTATCAGCAG

TTCAAGAAGTGGTTTAAAAAAGTAGGATGCCTCACTGACTCTCAAGCAAATATTGTCATT

AATGACACTTTATTCCAAATGGATTTTGCTGAAATTGGTATGGGGTTGAATGACTTTAAA

CGAAATATGAAGCATAAACTTAAAAGTAATCCGGAATTCGTCCATAGTATTCGTGAAAGA

CTGAATGATATTTTTGGTAAAGAGTTGATCTAG

>MT107270

ATGACAGAATCTGAACCATTGCAAGAAGAATCGAATTATATTCCTTACTGTATAGGCAAC

ATTCGCCATAATGGCGTTGTCAGTACCAGTAACCGTTTAATTCGCCCAATCGAATTATCA

GCCAATGAATATAAGGCACTTTTATATGCAATGGCGGTTGCGAATTATAGTGAGAAAAAT

AGGGTAGATGGTGAAATTACCGAACAAACTTATATTTATTTGTATAAGGATGATTTGGCA

GATTTACTAGGATTAAATAAACGTAATTCAATTAATGTTGCAATAGATCGGATTTATAAA

GAGCTTTCATCCCGGGTAGCTCATTTTATTATTGAGGAGCCAGCTGATAATGGTAAGAGA

AAGACTAAAAAAGTACATTCTGTCGTTCCCATTATCCGTGAGTTAAGATGGGAAGATGAT

TCTAAAAATGCGATTCAGATTCGTTTTACCAGTGAGGTGTTGCCGTATTTCACTCAACTG

GCTGGGGGTAATTTTACTACCTATCAGCTAAAACATCTTTTTGCCCTAGATTCTGTTGCC

AGTATGAGTCTTTATACTTATTTCATTAAAAACGAATTTAAGTACGCCAATCAAAAGAGT

TATGAAGTGCCATTACTTTTAGAAAACTTGAAAGCACTGATTGATATTAATGAAACCAAA

TATGATCGCTGGGTAGATTTTAGACGCTATGTATTAGACAAAATAGTGGCAGAAATTAAC

GAAAATACAGATCTACAGTTAGAGTATGAAACCGTTAAGAAAGGCCGTCCAATTGTAGGT

GTGAATTTTAAACTGCATCATCGGATTGCTGATAAGGCACTGGATGAATTTGCAGTGATT

GAAAAAATCTATCTTGATGTTCCATTTGAAGACAATGCATCCGTAAAAGAATTAGGGGCT

AAATTCGATATGAATGTACGTTCTTGGTATATTTTTAATAATGATGAAAATTATCAGCAG

TTCAAGAAGTGGTTTAAAAAAGTAGGATGCCTCACTGACTCTCAAGCAAATATTGTCATT

AATGACACTTATTCCAAATGGATTTTGCTGAAATTGGTATGGGGTTGA

>CP094556

ATGACAGAATCTGAACCATTGCAAGAAGAATCGAATTATATTCCTTACTGTATAGGCAAC

ATTCGCCATAATGGCGTTGTCAGTACCAGTAACCGTTTAATTCGCCCAATCGAATTATCA

GCCAATGAATATAAGGCACTTTTATATGCAATGGCGGTTGCGAATTATAGTGAGAAAAAT

AGGGTAGATGGTGAAATTACCGAACAAACTTATATTTATTTGTATAAGGATGATTTGGCA

GATTTACTAGGATTAAATAAACGTAATTCAATTAATGTTGCAATAGATCGGATTTATAAA

GAGCTTTCATCCCGGGTAGCTCATTTTATTATTGAGGAGCCAGCTGATAATGGTAAGAGA

AAGACTAAAAAAGTACATTCTGTCGTTCCCATTATCCGTGAGTTAAGATGGGAAGATGAT

TCTAAAAATGCGATTCAGATTCGTTTTACCAGTGAGGTGTTGCCGTATTTCACTCAACTG

GCTGGGGGTAATTTTACTACCTATCAGCTAAAACATCTTTTTGCCCTAGATTCTGTTGCC

AGTATGAGTCTTTATACTTATTTCATTAAAAACGAATTTAAGTACGCCAATCAAAAGAGT

TATGAAGTGCCATTACTTTTAGAAAACTTGAAAGCACTGATTGATATTAATGAAACCAAA

TATGATCGCTGGGTAGATTTTAGACGCTATGTATTAGACAAAATAGTGGCAGAAATTAAC

GAAAATACAGATCTACAGTTAGAGTATGAAACCGTTAAGAAAGGCCGTCCAATTGTAGGT

GTGAATTTTAAACTGCATCATCGGATTGCTGATAAGGCACTGGATGAATTTGCAGTGATT

GAAAAAATCTATCTTGATGTTCCATTTGAAGACAATGCATCCGTAAAAGAATTAGGGGCT

AAATTCGATATGAATGTACGTTCTTGGTATATTTTTAATAATGATGAAAATTATCAGCAG

TTCAAGAAGTGGTTTAAAAAAGTAGGATGCCTCACTGACTCTCAAGCAAATATTGTCATT

AATGACACTTTATTCCAAATGGATTTTGCTGAAATTGGTATGGGGTTGAATGACTTTAAA

CGAAATATGAAGCATAAACTTAAAAGTAATCCGGAATTCGTCCATAGTATTCGTGAAAGA

CTGAATGATATTTTTGGTAAAGAGTTGATCTAG

>CP041290

ATGCAGAAAATTATTGGCTCACAAGGCTTAGAGGACTATACACAATCTAATACAAGGGCA

AAATTGACCGATTTAGTTGTGACCCGCAATGATTTTCCTACTGCCAGATATAGCATTGAT

CTCAATCTTGAGAAGCTTATGTACTGTGCAATGATCATCGTGAGAAAGAATGAGCTTAAA

AATAAGACCTTAATTACTCATGACGACTTCATTTATGTGAGCAGCGAAAACTTTGGAGAA

TTGACCTCCCCAATGGCTAGAAAGGAAGTGCTTACTGCAACAGATAAACGTGAAATTCAG

CGAAATGCTGAAACTGCTTTAAAACGTATTTATACAAAATTTGATAACCCAACTATGTTG

GTTAAAGATGGGGAATCTGACGAACCTGCTAAAGTTCCGATGATGACTTATTGCCATTAT

GACAAAGCAACTAAATGTATTAAGGTTAGATTCGCAAAAGAATTTTTTGAGTATTTCTAT

GATCTAGTTAAGAAAGTAGATGAAAAAACTAAGTCATTTAGTAGCCATGAACTGAAGCAT

ATTATCTTATTTAATTCGAGCTATTCCCTTCGTTTGTATCGGATCTTAATGAGCTATATG

TGGCGTACATCAGAGGTCACTATTGATCTGGAAGAATTAAGATGGATGCTTGAATGTGAG

GATAAATATAAAGAGTTGGCCAATTTTAAAAACCGTGTTTTGAATGTGGCTCAGGATGAA

ATTAACGAACTTAGCAATATTAATGTCAGTTTTGAGAACGTAAAAAACGGAAAGGAAGTA

GTTGCTATTAAGTTTATTTTTAGCTTGAAAACTGAATATAAAGAGCAAGGACACATCAAA

TTTATAGATAAAATGAAAAAGGGCTATCTGGCTGCTGCAATTCCATTTAGTGATGATGGA

TCACACTTTAAAGCACCTGATCGTATTAAGCATTTCAAACCACCAGTAAAGGTATCTCCA

AAACAAATCAGCACCTTAGTAAATTGCAAAGAATTTTTAAATGATTACGGATATTTCTTA

GGTAATCTAGACGAGGATACTTCTAAGGTAATCATGAGAACTCTATTAACTGAAAAGTTA

GATAAGCTTAATGCTCATAAGCCGATAGATATGGATTATTACTTCTGGTTACAGGCAAAA

CGAGGGATTATCACCAATAGCAATAATGATAAGAAGAACGATCAGGACACAGACAATCAG

GACACGGACAATCAGGATTAA

>CP041297

ATGACAGACACTGACCACACAGAAGAACAAGAATATCTACCCTATTGCATTGGCAATATC

CGACAGAATGGGGTAGTGAGTACCAGTAATCGTTTAATTCGACCTATTGAACTTTCTACC

AATGAATATAAGGCTCTGCTATATGCAATGGCTGTGGCTAATTATGGTGAAAAAAACAAT

CAGGATCGTGAAATTACCGAGCAGACTTATATTTACTTACATAAAGACGATCTTGGTGAA

TTGTTAGGTCTCAACAAGAAAAACTCTATTAATGTGGCGATTGACCGGATTTATAAGGAA

CTCTCATCACGTGTCGCTCACTTTGTAATTGAGGAGCCGGCTGATGATCCTAAGAGGAAG

GTGAAAAAAGTACATTCGGTAGTGCCGATTATTCGCGAATTACGCTGGGAAGATGATTTA

AAAAATGCTTTACAGATTCGTTTCACCAGTGAGGTTTTGCCTTATTTTACCCGTCTCGCA

AATGGCAATTTTACTACTTACCAATTAAAGGATCTGTTTGCCTTGGATTCTGTTACGAGT

ATGAGTTTATATTCGTACATTGTGAAACAAGAGTTTAAATACGCCAATCAAGACACTTAT

GAAGTAGAATTGTCACTAGAAGGTTTGAAGGCGCTGATAGATATTGGTGAAACAAAATAT

GATCGCTGGGTTGATTTTAGGCGTTATATTCTCGATAGAATTGTAGCGGAAATTAATAGC

AAAACGAGTCTGAAATTAGAGTATGACACCATTAAAAAAGGGCGGCCAATCGTTGGTGTT

CGATTCAAAATATTGAATGACAAAAAAGAAAATGCTATTTCACAATCAAAGGAAAAAACT

AAAATTTATCTGGATGTAGATTTTAATGACAATAATTTGGTTAAAGAACTAGGGGCGAAG

TTTGATATGACAGTGCGGTCTTGGTATATCTATGCTAATGATCCAAATAGTCAGAAATTG

AAGAAGTGGTTCAAACCAGAAGGTTGTTTAACGGATTCTCAGGCTAATGTCATAGTAAAC

GATAATTTATTTCAAATGGAGTTCGCTAAGCCTGGCTTGTCTATGGCAGAGTTTAAAAAA

GAAATGAAACATAAGCTGAAGAATGATCGTGATTTCGTTCAGGCTAATCGTACTCGTTTA

AATGAAATTTTCGGGAAAGAAATTATTTAG

>MK134375

TATTTAGCATTAATTGAAGCAGAAGATTCTCTTTTTAAAAGACAATTTACGATTACCAAT

GAAGATGGAACTTTAACAAAAAGTCGCTGGATTCAAGATGCTAATTATCGGAAAGGTGAA

GGAAGAATTTTAGTTACCTTAACTCGTGTGGTAATTGAACATGTCACTAAAATAGATGGG

TTTGAACAGTATTTTACTAGTTATCATTTGAAAAAAACTGCTGACTTCAAAAGCGTTTAT

GCAGTACGTCTTTATGAGCTCTTAATGCAATGGAAGTCTGTAGGGAAAACGCCTGTTTAT

GAATTAAATAAATTTCGTAGCCAACTTGGTATAGGTGTTAACGAATACACTCGAATGGAA

GCATTTAAGAGACGTGTTTTAGATATTGCAGTAGATCAAATCAACGAATTTTCAGATATA

ACTGTTAAATATGAACAACATAAAAAAGGACGTTCAATTTCCGGTTTTTCTTTCAGTTTT

AAGCCAAAGAAAGCAACTATTCGATCTATAGAAACTAATAGAGACCCTAATACAACTGAT

CTGTTCTCGAGAATGACAGATAAGCAACGCCACTTATTCGCTACTAAACTTTCCGAACTA

CCAGAAATGGGTAAGTATTCTCAGGGTACAGAAAGCTATCCACAATTTGCTATACGCATT

GCAGAAATGCTCCAAGATTATCAAAAATTCCAAGAATTATTTCCTTATCTTCAAAAAGTT

GGCTATCAAGCTGCCTAA

>CP084298

ATGACAGAATCAGAATCATTGCAAGAAGAATTGAATCATATTCCTTACTGTATAGGCAAT

ATTCGTCATAATGGCGTTGTTAGCACTAGTAACCGCTTAATTCGCCCAATTGAGTTATCA

GCCAATGAATATAAGGCGCTCCTATATGCAATGGCTGTTGCAAATTATAGTGAGAAAAAT

AGGGTAAATGGTGAAATCACAGAACAAACCTATATTTATCTGTATAAGGATGATTTGGCA

GATTTACTAGGATTGAATAAACGCAATTCGATTAATGTTGCAATAGACCGGATTTATAAA

GAACTTTCATCTAGGGTGGCTCATTTTATTATTGAGGAACCTACTGATGATGGTAAGAGA

AAGACTAAAAAAGTACATTCTGTCGTTCCTATTATCCGTGAGTTAAGATGGGAAGATGAT

TCTAAAAATGCCATTCAGATTCGTTTTACCAGTGAGGTGTTACCGTATTTCACGCAGTTG

GCTGGGGGGAATTTCACCACCTATCAATTGAAACATCTGTTTGCCTTGGATTCTGTTGCT

AGTATGAGTCTTTATACGTATTTCATTAAAAATGAATTCAAGTACGCAAGCCAAAAGAGT

TATGAAGTCCCAATGCTTTTAGAAAACTTGAAAGCTCTGATTGATATTAATGAAACTAAA

TATGATCGCTGGGTAGATTTTAGACGCTATGTATTAGATAAAATAGTTGCAGAAATTAAC

GAAAATACAGATCTACAACTAGAGTATGAAACCATAAAAAAAGGGCGCCCAATTGTGGGG

GTGAATTTTAAACTGCATCAGCGTATTACTGATAAAACACTAAATGAAACTGCTGTAATT

GAAAAAATTTATCTTGATGTTCCATTTGAAGAGAATGCATTAGTAAAAGAATTGGGGGCA

AAATTCGATACGAATGTACGTTCCTGGTACATTTTTAATAATGATGAAAAGTATTCGCAG

TTCAAGAAGTGGTTTAAAAAAATTGGTTGTCTCACTGACTCTCAAGCAAATATTGTTATT

AATGACACTTTATTCCAAATGGATTTTGCTGAAGTTGGTATGAGCTTGAATGACTTTAAA

CGTAATATGAAGCATAAACTAAAAAATAATCCGGAATTTGTACAAAGTATTCGTGATAGG

CTGAATGATATTTTTGGTAAAGAGCTTGTCTAA

>CP090068

ATGTCAGAACTAAGCCATAACAAAGAAGAGCTGGATTCAATCCCGTACTGCATAGGTAAT

ATTCGTCACAATGGGGTTGTAAGTACTAGCAATCGTTTGATACGACCTATTGAATTGTCA

GCGAATGAATATAAAGCACTTTTATATGCGATGGCTGTTGCCAATTACGGTGAGAAGAAT

CATATCGATAAAGAGATTAGTGAACAAACATACATCTATCTGTATAAAGATGATTTAGCT

GAATTACTGGGATTAAGTAAGCGAAATTCAATTAATGTAGCCATTGATCGAATTTACAAA

GAACTTTCATCCCGTGTGGCGCACTTTGTAATTGAAGAACCTGTAGATGATATAAAAAAA

AAGACTAAAAGAGTTCACTCGGTTGTCCCTATTATCCGTGAGTTAAGATGGGAAGATGAT

TCAAAGAATGCACTTCAAATCAGATTTACCAGTGAAGTATTACCTTACTTTACTCAATTA

GCTGGTGGTAATTTTACGACATACCAACTAAAACATTTATTTGCTCTAGATTCAGTTGCA

AGTATGAGTCTTTATACCTATTTCATAAAAAACGAATTCAAATTTAAGACTCAAGAAATC

TATGAAGTTCCATTATTATTAGAGAATCTCAAAGCTCTAATTGATATAAATGAAACTAAA

TATGACCGTTGGGTCGATTTTCGACGTTATGTATTAGATAAAATTGTATCTGAAATTAAT

GAAAACACCGATCTACAGTTAGAATATGAAACCATTAAAAAAGGACGCCCTATTGTAGGC

GTAAAATTTAAACTACATCATCGTGGTTTTGAAAAGCCTGTAATTGAAAATAAAAAGGCC

GAAAAATTAAAGATCTACTTAGATGTTCCATTTGAAGATAATGCTGAGGTAAAAGATCTA

GGTGCAAAATTCGATACCACTGTACGTTCATGGTATATTTTTAATGATGATGCTAATTAT

TTACAGCTTGAAAAGTGGTTTAAGACTGTAGGTTGCCTCACAGACTCTCAAGCAAACGTT

GTTATCAATGATTCATTATTTCAAATGGATTTTGCTGAAGTAGGAATGAGCTTAAGTGAC

TTTAAGCGAAAAATGAAAAATAAATTGAAAACTGATAGTAAATTTGTGGAAGACATCAAG

GATCGTCTAAATGAAATTTTCGGAAAGGATATTATTTAG

>CP090315

ATGACAGAATCTGAACCATTGCAAGAAGAATCGAATTATATTCCTTACTGTATAGGCAAC

ATTCGCCATAATGGCGTTGTCAGTACCAGTAACCGTTTAATTCGCCCAATCGAATTATCA

GCCAATGAATATAAGGCACTTTTATATGCAATGGCGGTTGCGAATTATAGTGAGAAAAAT

AGGGTAGATGGTGAAATTACCGAACAAACTTATATTTATTTGTATAAGGATGATTTGGCA

GATTTACTAGGATTAAATAAACGTAATTCAATTAATGTTGCAATAGATCGGATTTATAAA

GAGCTTTCATCCCGGGTAGCTCATTTTATTATTGAGGAGCCAGCTGATAATGGTAAGAGA

AAGACTAAAAAAGTACATTCTGTCGTTCCCATTATCCGTGAGTTAAGATGGGAAGATGAT

TCTAAAAATGCGATTCAGATTCGTTTTACCAGTGAGGTGTTGCCGTATTTCACTCAACTG

GCTGGGGGTAATTTTACTACCTATCAGCTAAAACATCTTTTTGCCCTAGATTCTGTTGCC

AGTATGAGTCTTTATACTTATTTCATTAAAAACGAATTTAAGTACGCCAATCAAAAGAGT

TATGAAGTGCCATTACTTTTAGAAAACTTGAAAGCACTGATTGATATTAATGAAACCAAA

TATGATCGCTGGGTAGATTTTAGACGCTATGTATTAGACAAAATAGTGGCAGAAATTAAC

GAAAATACAGATCTACAGTTAGAGTATGAAACCGTTAAGAAAGGCCGTCCAATTGTAGGT

GTGAATTTTAAACTGCATCATCGGATTGCTGATAAGGCACTGGATGAATTTGCAGTGATT

GAAAAAATCTATCTTGATGTTCCATTTGAAGACAATGCATCCGTAAAAGAATTAGGGGCT

AAATTCGATATGAATGTACGTTCTTGGTATATTTTTAATAATGATGAAAATTATCAGCAG

TTCAAGAAGTGGTTTAAAAAAGTAGGATGCCTCACTGACTCTCAAGCAAATATTGTCATT

AATGACACTTTATTCCAAATGGATTTTGCTGAAATTGGTATGGGGTTGAATGACTTTAAA

CGAAATATGAAGCATAAACTTAAAAGTAATCCGGAATTCGTCCATAGTATTCGTGAAAGA

CTGAATGATATTTTTGGTAAAGAGTTGATCTAG
