# Supplementary material for: Classification and molecular characteristics of tet(X)-carrying plasmids in Acinetobacter species
Source: Front Microbiol. 2022 Aug 23;13:974432. doi: 10.3389/fmicb.2022.974432 (PMC9445619; doi:10.3389/fmicb.2022.974432)
Supplement: Supplementary file 3 [file Data_Sheet_2.DOCX]

>GR1_CU468231

MKKICVLMKKELVVKDNALINASYNLDLSEQRLILLAILEARQSNTPNDKDLTIHAESYI

NHFNVHRNTAYKVLKDACKSLFDRRFSYQKLTQKGNIENVISRWVQRISYVENEALVRIK

FSDDVVPLITNLEKHFTSYELEQVSSLTSVYAIRLYELLIAWRSTGKVILVELEELRLKL

GIESHEYKRMGQFKEKVLHLAIDQINKYTDIKAEYEQHKRGRSIIGFSFKFKQKQQPQKA

DSKRAPNTPDFFVKMTDAQRHLFANKMSEMPEMSKYSQGTESYQQFAIRIADMLLEPEKF

RELYPILEKAGFKG

>GR2_NC_010605

MRDLVVKDNALINASYNLDLVEQRLILLAIVEARESGKGINANDPLEVHADSYINQFGVH

RNTAYQALKDACKDLFARQFSYQEKKANGNIRNVMSRWVSQIAYNDNEATVDLIFAPAVV

PFITRLEEQFTKYELQQVSSLSSAYAIRLYELLIQWRSTGKTPTIELQEFRKKLGVLDNE

YLRMAHLKERVLELSIKQINEHTDITVKYEQHKRGRSISGFSFTFKQKKKDSPSIERDPN

TLELFSKMTDAQRHMFANKLSELPEMGRYSQGTESYQQFAVRIAEMLQDPAQFKELYPYL

KKVGYMPSNKKDTVNG

>GR3_1_GU978997

MKTELIVKDNALINASYNLDLVEQRLILLAIVEARESGKGINANDPLTVHAESYINQFGV

HRNTAYQALKDACDDLFARQFSYQSLSEKGNIINHKSRWVSEVAYIDNEAVVRLIFAPAI

VPLITRLEEQFTKYEIQQISNLTSAYAVRLYEILIAWRSTGKTPLITLSDFRQKIGVLDT

EYKRMYDFKKYVLDIALKQVNEHTDITVKVEQHKTGRSITGFSFSFKQKKSVTKSAKSIG

VSEDITITLTDAQRYSFASKLSELPEMGKLSQGTESYEQFAVRIADMLKQPEKLKELTPL

LRKVGFQ

>GR3_2_GU978996

MKTELIVKDNALINASYNLDLVEQRLILLAILEARESGKGINANDPLTVHAESYINQFGV

HRNTAYQALKDACDDLFVRQFSYQSLSEKGNVINHKSRWVSEVAYIDNEAVVRLIFAPAI

VPLITRLEEQFTKYEIQQISNLTSAYAVRLYEILIAWRSTGKTPLITLYDFRQKIGVLDT

EYKRMYDFKKYVLDIALKQVNEHTDITVKVEQHKTGRSITGFSFSFKQKKSATQSVGSKR

DPNTLDPFSTMTDKQRHLFASKLSELPEMSKYSQGTESYQQFAVRIAGMLQDTERFREIN

SFAQKK

>GR4_GU978998

MRDLVVKDNALINASYNLDLVEQRLILLAIVEARDSGRGINANDPLEVHAESYVNQFNVA

RQTAYQALKDACKDLFVRQFSYQEINKRGNVENVLSRWVSEIRYIDDEATVKLIFAPAIV

PLITRLEEQFTKYELQQISNLSSAYAVRLYELLIAWRSTGQTPIIELAEFRKKIGVLDDE

YTRMGNFKDRVLNLAIAQINEHTDIKVQCQQHKKGRNISGFSFTFKQKKVVIANNKKQTT

LEIFSKFTDAQRHFFANKLSELPEMNKYSQGTESYSQFAVRISEMLKDLQKFEELLPYLE

KVGFNAK

>GR5_GU978999

MRDLVVKDNALINASYNLDLVEQRLILLAIVEARESGKGINANNPLEVHAESYINQFNVA

RQTAYQALKDASKDLFARQFSYQEMNKRGNIENVLSRWVSEIRYIDAEATVKLIFAPAIV

PLITKLEEQFTKYELQQVSNLSSAYAVRLYELLIAWRSTGQTPVIELEEFRKKIGVLDDE

YTRMGNFKDRVLHLAIDQVNEFTDITVKYEQHKKGRSIYGFSFSFKQKKNVNKPNLEARD

QNTLDIFTKLTDAQRHLFANKLSELPEMSKYSQGTESYPQFAVRIAEMLLDAEKFKELYP

YLVKVGFQTK

>GR6_CP002524

MGILMTVNSVNLNSKKDFIINRLYENLPKKPYCTSDFFGLKIRDKKQAIRHSHIQINHPN

FKRYIVIDADYPGAATAWRYDFDDNIPVPNLIVVNPENTHCHFYYELEAPVSFTESSSKR

AQEFYNSVSKKLTEVLKGDSKYVGLIAKNPAHEKWIVEVPRLEKYSLHELVEHLELKPHE

YRNINSEKTGIEKFVINGRNDHLFNEIRHQAYIDIRSYRSKTFVEWFDHVKSLLINANKN

FSVPLPYSEVCATAKSIAKYCWKKDSYCFQEFCERQHIKAKKGGRAKSDKYVEMRRTAAR

LLRSGKTKTYISELLQVSYRSVLRWLQGIKVQAAIMHLSELKKLCDNAQNQILACFIASL

IVIILDEHIYDFNESEELKITIKFKLKVPIQ

>GR7_CU468233

MKNSLVVKDNALINASYNLELTEQRLIMLAIINARESGQGITADSKLEIHASDYAKLFNV

SIDASYKALKEAVNNLFNRQFSYTAEYKRTGKTGVVRSRWVSRIFYVDDLALLEITFAPD

VVPLVTRLEEHFTSYQAKQVAHLTSKYATRLYELLIAWREVGKVPQIEISTFRNRLGLLE

NEYTAMSDFKKRVLEPSIKQINEHTDITVTYEQHKKGRLISGFSFKLKQKQQPKIEVKRD

PNTPDFFVKMTDAQRHLFANKMSEMPDMSKYSQGTESYQQFAIRIADMLLEPEKFRELYP

CLEKAGFQPA

>GR8_1/GR23_GU979000

MSELIVKDNALIQASYTLDTVEQRLILLAIAEARETGHGINENSLLQVHASSYINTFNVE

KHTAYTVLRDASKSLFDRYVTYHDINPKTDKDRSFHCRWVDKIGYEPQSGIVFLRFTQDI

VPLITRLEENFTKYELQQVSRLTSSYAIRLYELLIQWGSRGKTPTFDLHVFRNRLGVEDG

QYKTMCNFKQFVLDFALKQINQFTDIIAKYEQHKSGRKITGFSFTFKFKNNKNVKEKLVE

KTEFYKLTESQLDLFAKKLAHLPELGHLADEGMSYEEFYSKLKSILKDPEQQKKLVPYFE

KAGLNPK

>GR8_2/GR23_AY541809

MSELIVKDNALIQASYTLDTVEQRLILLAIAEARETGHGITENSLLEVHASSYINTFNVE

KHTAYTVLREASKSLFDRYVTYHDINPKTGKDRSFHCRWVDKIGYESQSGIIFLRFTQDI

VPLITRLEENFTKYELQQVSRLSSSYAIRLYELLIQWRSAGKTPLFDLSIFRQQLGVKPH

QYKTMSNFKTYVLDFALKQVNELTDITAKYEQHKKGRSISGFSFTFKQKKMSNLPIKNKR

DPDTIDIFSKMTDAQRHLFSHKLSELPEMGKYSHGTESYPQFAVRIAEMLQNPEKFKELY

PYLQKVGFKAA

>GR9_CU468233

MANDLVIKNNALIDASYTLSLVEQRLIGLALVKANNQHQEITSDTVLTIHAGEYAQQFNV

DGSVAYRALKEASERLFLRYFSYTLYGLDFGKEYTLKRPKKLKDGDIPTIMKSRWVQKVG

YTESEGLLHFQLTSDVVRLVANSKEYFTSYYLSQTTEFTSTYATRLFELLMKWKNVGHIP

FIEIEQLRGQLGVEPKQYKIISNFKLRVLDVAVEQVNQYSDYKIEYEQHKQGRTITGFSF

KFQPKATKTKKIESRRDPNTPDFFIKMTDAQRHLFANKMSEMPEMIKYSQGTESYQQFTI

RIADMLLQPEKFRELYPILEKAGFKG

>GR10_NC_010605

MKTELVVKDNALINASYNLELAEQRLILLSIVKARETGRGITSDSRLEVHASDYMKQFNV

EKSAAYEVLKSASESLFNRYFSYKEQRHDGTEFVVKSRWVSRVAYAPNVAILEVTFAPDV

VPLITRLEQHFTSYQLKQVSQLTSKYAIRLYEMLIAWRNVGKCSFELINLRDSLGIASDE

YKQMGHFKSRVLDASIAQINEYTDIKVTYEQQKNGRTISGFTFKLKPKQVQQEITILDTK

ASLIPSDLTPNQRVTFASKLSKLQELGGKAEPGEEVEAFAKRIELWLEDEKKLKMLTPFL

YQVGFKKAKPKKVSQ

>GR11_NC_010401

MNKENSYDKSYPVTTMAIQNKVTECFKSMSVDEKRILIMASPIARNVDASEQDQILISAQ

QFADDCGIKVNSAYKQIENASKKLVDRSFSYVNDRGKKVYSNWVIDATYEDAGISLRFTS

IVLVMLKILDKYNPYTRYKKDVVLKLKKDYSIDFYHLAKKNQAKNGFELTLDEMFTEFGL

PESYRDLRNLKRRVLKSSLDEINEFTDVTVDYSPVKKGRSVVGFKFTVKEKSKPKLIAPE

RDPKTIDMFCNLSDAQINKYSAILSKLSELSDLSNFQDYPSFALWISGILRDPKSVREET

AKRIFKALHSKTDFKP

>GR12/GR29_CU468232

MNKNHVVKSNQVIEASYQLSAVEQRIVLAAISRIPKNQPITDDELYPVSINELRQLGVHE

KTAYRDLKEGINRLYERSINLSIDDKSIKMRWVQEVQFLDSQSVIGIRFSKPILPFISNL

SREFTKYALSDIAGINSGYGIRIYELLVQYRQIGKREISVENLRTMLELGKKYPLFADFK

KRVIDTAIDQINECSPLNVTYEQKKTGRKVTSIIFSFKEKTKSISHQNTDVPKEFYKLTD

AQINMFGNQLSRLHELSHLAQQGESYDDLAITIKDMLRDPKQQKQFLPYLKNLGFKL

>GR13_NC_010404

MSNHNEPEELEHLPYCIGNIRHNGVVSTSNRLIRPIELSSNEYKALLYAMAVANYGEKNN

QDREITEQTYIYLHKDDLGELLGLDKKNSINVAIDRIYKELSSRVAHFVIEEPVDDNKRK

VKKVHSVVPIIRELRWEDDAKNALQIRFTSEVLPYFTRLANGNFTTYQLKDLFALDSVTS

MSLYSYFIKNEFKYANQDSYEVELSLENIKALIDIGEKKYDRWVDFRRYVLDKIIEEINE

RTSLQLEYDTIKKGRPIVGVRFKILNRHATEVVVSNTNDKTQIYLDVNFDDNALVKELGA

KFDMTVRSWYIYANDPNYKQFSKWFKTEGCLTESQANVIVNDIMFQMDFAVAGSSMSEFK

KEMKYKLKNNPNFVKENRKRLNEIFGKDVI

>GR14_NC_010403

MQNLDKKKPLLSDSLATGDNKGFASPKGDQHRDRITRFGILKHRSKQQENYLFSLAKIKE

NYHADVKNDESIRAMKTAQKLNGCGNFLLFKNFYTINQIKLAKFQACSEHLLCPFCAGIR

ASKAIQKYSERVDQVLSENPRLKPVMITFTVKNGVDLGERFTHLIKSFRTLIERRRDYIK

KGRGFNEFCKINGAMYSYENTYNEKTNEWHPHIHMFALLDDWIDQDELSQYWQSITGDSM

VVDIRRAKKQKDLGYSGAAAEVCKYALKFGDLSVEKTWEAFKVLKGKRLSGAFGSLWGVK

IPESLIDDLPDDSDLPYLEMIYKFVFSKKSYYDLQLTRHVEPTGKDDADELRGEEGRNLL

VSMDGRGASDAGRARTGALAPQHGRKKQHWQIPPVTRVRVRKRIRRWDGYLCVLHL

>GR15_CU468233

MEIKMMSPSKKELVVKSNQVIEASYQLSSTEQRIVLAAISKISRAEDITDDEIYRVTIDD

LKKLGVHEKTAYRDLKDGVNRLYDRSINLAINDESIKMRWIQSIRFLESKSVVGIRFSKE

ILPFISNLSREFTKYSLSDIAGMSSAYAIRIYELLSQYRSIGKREIPIESLRSMLELGKR

YPLSADLKRWVIDTAVDQINEHSPLNVSYQQIKTGRKVTHIQFTFKEKSKNIEHKSEQND

FYKLTDSQINMFGNQLSRLHEVSHLAQQGESYDDLAIKIKDMLRDRIQQKQLIPHLKNLG

FKA

>GR16_L77992

MSMNIFYGENPSSASLKSLENSQSLGIKTKSHVMLTPQGFQRVHDYLLQDQSRKLLPKER

VSKCRRLRIDKTKTRTVMYNEHREKAHYGNVQICGSIWSCPVCAKQITQKRRNELGKGIE

SWKTVHNGSVYLLTLTFSHSPDQSLKSNLEGLKRAMKRFYETTRVQAIFKKLSVFHKIKG

LEVTYGQNGWHPHHHVLLLAEHHDLRFKDYTSELTELWIKACIKSGLNAPSMRHGLDLRN

GSYADQYVSKWGLEDELSKGHVKKGRNGGFTPFDLLNFSIEDNEIYGKKPSKLFQEFAIS

MKGARQLVWSRGLKKLLGIEEKSDEELAVETDKASITLNRVEDLVFELLCRYQLRHQYLE

AIKHDYETGSFGSGLADQLIEQVVNYEIKQMQQVFS

>GR17_CP000522

MAKLSLSEVSKRFNVSRSTLYRAIKEGRISRNADGYFDVAEVIRCFGEPSKKHEQNQEID

KPKDDTDLRQLVDFMRKEIDSYKDREKRYLDQIDRFQLLLGHKESEEKMSHDTSVRQTND

TPCDNNHETHKDTVNQEFYSTESPHDTPLTHLNEAPQNISKTHHETKKKRGLFGRVLNAV

FDND

>GR18_CU468232

MLFSYLITRNTKMSKLLVVKANNIIEASYQLSLNEQRLILAAIACIPKGEEVTDNTGYCV

TRESFIELGVNPKTASREIREACDRLFNRVITITTEAGTFKTRWVQDIMKYNSDWALANP

EFIQEVAGSDPYAEDYILAAIRFSKSVLPFISNLSSNFTQYFLQDIAGVSSGYSVRFYEL

MMQFKSTGYRKIRLDDLRNMLDLNNKYPLTADLKRWVIDTAIDELNEKSPITIKYKLLKT

GRKFTHIELKFKQKLSPKKIESQRDQKTIDMFSNLSDSQIKTYSSVLSKVHSISDLADNK

DYSAFAIWIANVLRDPTSVREETAKRIFKTLRTETDFKG

>GR19_GQ861437

MLIVKDNALINASYNLELVEQRLILLAIIEARQNGKGINTNDHLIVHASTYIEHFNVEKH

SAYMSLKEACKNLFARQFSYEEINPNGSSTQYTSRWVSKIGYTKKEGTVHIIFAPDVVPL

ITRLEKHFTSYELEQVAQLQSKYATRLYEILIAWRSTGKVPEISLSEFRAKLGVSDSEYK

IISNFKLRVLDVAVSQINKYTDITVTYEQHKKGRTIIGFSFRFKQKQLAKKIESKRDLNT

PDFFIKMTDAQRHLFANKMSEMPEMSSYSQGTES

>GR20_NC_012813

MRELVVKDNALINASYNLDLVEQRLILLAIVEARESGKGINANDPLEVHAEGYINQFGVH

RNTAYQALKDACNDLFARQFSYQKINERGNIENYRSRWVSEIGYVDNEAVVKLIFAPAIV

PLITRLEEHFTKYELQQVSNLSSAYAVRLYELLIAWRSTGSTPIIEVSDFRQRIGVLDTE

YKRMERFKTSVLELAIKQINEHTDITVKYEQHKRGRSISGFSFTFKQKKKDNPPIERDPN

TLDLFTKMTDAQRHLFANKLSELPEMGRYSQGTESYPQFAIRIAEMLQDPDRIKELYPYL

KKVGYMPSNKKDTVNG

>GR21_KY984046

MGELVVKSNDLINASYNLGVVEQRLLLLCIIAARKKDRVLSPSDIFYIHASEYIEQFDVD

RSVAYRALAEGIKGIYDSEIKLTSKNSRKKINIRWCWKAEYDEDHATVGVAFTDDVIPLI

SALEQRFTSYDIDQIAKLTSKYAIRLYELVIAWRSINKTPVFELEDFRNKLGLGVSEYKT

MSNFNSNVLNIAIQQINKFTDIKIKVHKHKKGVRIVGFSFELTQRKMKNQNSTKDTFYRL

TDSQINMFGNQLSRLHEVAHLAVEGESYEILAAKIKEMLRDPIQQKQFLPHLQNLGFKA

>GR22_KY984047

MKNDLVVKDNALINASYNLDTTEQRLILLAIVQAREVSKDVDANSTLEVHAHHYMKQFNV

DKHAAYEGLKNAASNLFERKFSYKGIHEGTQQEKIVKSRWVSKIAYVDSAGIVELTFAPD

VIPLITQLEKSFTAYELKQISSLTSKYAIRLYELLIQWRSVGKTPMFDIDDFRFKLGLAE

GEYAKMANFKVRVLDIALNQINELTDITASYEQHKVGRTISGFSFSFKPKQHVDAITHNK

PKKLTDKQIQFFANKLAHHDPFASKKAAVGESYADLEKRLLIELQDVEFVRKYASVLKEL

GLEV

>GR24_AFCZ02000003

MAELIRNSDVYKANALINASYALDTAEQRIILLAILVSRNKNADLTAETIIEIPASLYAQ

KFNTTVSAAYKTLKEAEDTLFERRFSYTTMRNGKIEVVRSRWVSRVSYVKDDALLTITLA

PDVIPLVTKLEGTFTKYAIDNLRDVTSKYGIRLYELVASWKNSDIRKTPVYDFEDFRAKM

GLLPHEYRDKKNPESTDMTNFNKRVLKPAIDQINSFTDLFITEKKIKTGRNITGIYFEVS

LKTDNFIEGEAKEIHDSKPSSDSAKKTGTPLENIRLPKVSTQEFLGSDLSEEDLNKENPL

KEFIVESGVYKSAIEKPVEEKDEFELNGIKRLYEALLKLDEGVTKEYVREYAQIKGVTLQ

HALIELYNSKRPA

>GR25_NC_017848

MITSNIFATDNKSILISRLYENLPRKPYCTNDFFGLRIREKKSAISHSHIQFNHPSFKRY

IVIDADYAGAATAWRYEFAENIPVPNLIVTNPENSHCHFYYELSAPVSFTDSSSKKAQEF

YNAVSKKLTEVLRGDTNYTGLIAKNPAHEKWIVEAPRIETYSLHELVEHLELHPHEYRSI

PGQNSKQEQVQCINGRNDHLFHTVRVKAYVDVRDFRSKTYPQWEEHVRQLLVDHNLELNN

PLPYSEIKATAKSIAKYCWKKDGYCYQEFCDRQISKAKKGGQAKADKYIELRKKAVALLR

KGKNKRLIAQLLKVSYRSVLRWLYNVKLAAAIMHLRDLKNMCDNAQNQILAAFVASLAVL

FLDEFIYDFTQDDILTINLTFNIKMLI

>GR26_CP015365

MADLVVKSNKLVQALQTLTLSETRLLQLAIVDARETGQGLSAEEPLELNASRYATAFNVS

PDAAYLALVEAEDSLFKRQFTITNEDGTLTKSRWIQDANYRKGEGRILVTLTRVVIEHVT

KIDGFEQYFTSYHLKKTSDFKSVYAVRLYELLMQWKSVGKTPIYELNKFRSQLGIGVNEY

DRMEAFKRRVLDIAIKQINELSDITVKYEQHKKGRAISGFSFAFKQKKTNQPIADKRDPN

TLDLFSKMTDAQRHMFANKLSELPEMGKYSQGTESYQQFAVRIAEMLQDPEKIKELSPYL

KKVGYMPSNKKDTVNG

>GR27_AYFH01000057

MDENKKTYPPSWVVMQNNIQECFKSMNIDEKRILILASPIARTTQATEKDPIMITAEKFA

EECGIKTHSAYTQLEVASRNLIKRSFSYNNERGKRVLSNWVIDCIYEDGGIAIRFPEIVL

LMLTEFDKLNPYTKYKKDIVLSLKKDYSFDFYHLAKKHQAMGKFEMSLERIRTEFGLPES

YHDLSNLKKRVINPSLDEITANTDIALTYENVKKGRSVVGFKFTVREKPKPRLIASEREQ

ETLDIFRSLSDGQINTYSSILSKVGSISDLAGAKDYQAFAIWIANILRDPKSVREETAKR

IFKALRTETDFKG

>GR28_AFDB02000003

MTNTNKLVVKDNALIDASFNLSLIEQRIMLLAIVEARESNSLSPDTPIEVSVSDYIHQFK

VDSNNAYALLKDASKTLKRREFSYLDRYKGIEALSTANWVNKVTYVDKSGLIVLYLSHEV

ISLISKLSEQFTKYYIEQVSEFKSKYSIRLYELIIKWLSVAKTEKYSINDLRSKLGLGVE

EYSTMTNFKSNVLDKAINEINKHTDIIVDYQQFKKGRVITDIQFFIKSKARPSKQTNTTK

QSFYQMNDAQINLFGNQLSRLHELSHLANQGESYDELAIKIKDMLRDPIQQKQLLPHLKN

LGFKA

>GR30_AYOI01000002

MSSIVKSNPKVKKHNNLTQAHFFNVSVIAYRLILLAGTDKFLENMLKSGENTYIRITAHD

YHNLYGSSSDMSGSYKAIKDAPDDLLNAKLKYKRLKTESDPGRWVGGINWVQDARYNDEL

KCVEILFSTTVLPLLANVRKSFTYYNLRHIGRLSSMHSIRMYELMMMWRKSGKTPDLTVS

YMKNFLGVPDNEYSDPKELKFFTAQVIKKSVKEVTSKTNIEMDFEVVRGEKRATIGYSFS

HKLKALPEGEQPEQEELEDDNEGGGDPSKLLPNNDDDPELPF

>GR31_CP012956

MTESEPLQKESNYIPYCIGNIRHNGVVSTSNRLIRPIELSANEYKALLYAMAVANYSEKN

RVNGEITEQTYIYLYKDDLADLLGLNKRNSINVAIDRIYKELSSRVAHFIIEEPADDGKR

KTKKVHSVVPIIRELRWEDDSKNAIQIRFTNEVLPYFTQLAGGNFTTYQLKHLFALDSVA

SMSLYTYFIKNEFKYANQKSYEVPLLLENLKAVIDINETKYDRWVDFRRYVLDKIVAEIN

ENTDLQLEYETVKKGRPIVGVNFKLHHRIADKAPDEIAVIEKIYLDVPFEDNAFVKELGA

KFDTNIRSWYIFNNHENYQQFKKWFKKVGCLTDSQANIVINDTLFQMDFAEIGMGLNDFK

RNMKHKLKNNPEFVQSIRERLNDIFGKELI

>GR32_CP013925

MFGMTNNNLALASNDSLTIVLKRFYNNLPDKPYHSNGFDVEGLKINRKIEAIKKKYIQFN

HPKWKKYILIDIDRPGAVTDWLYESPHLPAPNLIIENRKNGHAHFVYELIDAVSFTERSS

LKAKNYYNAVEKALTSELGGDERYNGVVGKNPYSEEWRTSTYRTEAYHLKDLASKLELTT

MGLTPIEMPQKAQNDECAINGRNDEVFHSVRHLAYKDIRDFKNNADLLFNHWFDHVLKLV

QEKNSFFINPMDYKECTHIAKSISEYCWRNHEECYKQFVERQRNKGSKGGTSRSAKYEEA

RRMTKQLFRQGVSLKQIAEKLNISYRTAVRYTKGLLRIKLLSFNDINNLRKSALADKKAR

SEAKCIKSERSESINNAFNWCDSSQNQVLAARHTHPAPFGVFFKKLLSFTFKNLKFERLK

GGAIFYYYGKIP

>GR33_CP012005

MKKPKHDLTHVRHDPAHCLAPGLFRSLKRGDRKRCKLDVTYTFGEDESMRFVGFEPLGAD

DMRLLQGIVALGGPNGILLTPEPTSETGRQLRLFLEPRFEAIEQDGLVVRESLTKLLSET

GMTDSGDNIKALKASLLRMSNVTILVTKGRRQAAFHLMSHAFDETDGRLWVALNPRIAEA

ILGHRPYARIDMAEVRVLQTDPARLMHQRLCGWIDPGKSGRVELDTLCGYVWPDEANAEA

MKKRRQTARKALAELAAVGWVVNEYAKGKWEIKRPGPTATAPVYRRNVPLLPS

>GR34_CP047975

MSNIVYKDNNLVEASYSLNLSEQRLILIAIIAAREIEKELTSDTILTIHASEYMKQFNLG

RQASYEALQSACDNLFERHLNYKAVDPITGKIGIYKSRWVSKVGYVKEEGCVQLIFAPDI

IPLFVKLEEKFTRYELKQISPLTSIYAIRLYELLIRWRSTGKLYISIDELRSKLGLIEDE

YKKMGDFKKRVLTVALNQINKFTDITVSYIQKKEGRNISELHFMFEEKEQNKTSTSAPLE

PTYKLTAKQCIFFAKKLCDITNYPKFGNDFAHRGETLEDFQERISSDLLDSDNVRKYFSY

LLEVGYAPKYKK

>GR35_CP050420

MKNGLVVKDNALMNASYNLEVTEQRLILLAIINARETQQGITSDSKLEIHANDYANQFNV

KKETAYEALKNAVNNLFERQFSFKETTKKGVGVVRSRWVSRIKYIDDSGLLEITFAPDVV

PLITRLEQHFTSYQLKQVSQLTSKYAIRLYEVLIAWREVGKVPKIDLAEFRERLGIAANE

YKAMNHFKSRVLEPSIKQINEHTDITVSYDQHKTGRTITGFSFKFKQKNPLKITKQTEIK

RDPNTPDFFIEMTDAQRYLFAQKLSVLPEVGSEYAKIGESSEDFVKRLADMLLEPKKIKM

FYPLLEQLGFNSKKQQPIQKQVDEPKFLDLSDMQINRISYGMSRHKKFAHLKQEDESPDD

FMQKLRIMLKDPKQQIQFYDYLKANP

>GR36_CM016516

MDQALITQDSSVIKQANKLIESVYRMDANEQKIILLAAKFVYDMEKKKEEFTTNTEIVIT

AAEYANEYGITRQTAFEVISKAKNTLYERSFEYDYKNPETGEVKPMSSRWIHSKGEMKAK

SEISMFFAPAVIPLIYLVQQEFTLLDIKEIGRLKSKYAIRLYQILMKWRNADFQPKFEYQ

DLRAKLGVEDGDYTLMADFKKGVINVAVKQINQGTGFVGLKYSTVKKGNTITHFTFSYDK

YDNKTINVTPINSQSATRPKKPKKANQQLTEQKAPNSANESDLGDDFSLTPPEPKALKKV

KRHFEGGMTEPQAYMFAGKIVNKIKEGDTRFMYLSKLAQEGELDSSFSKRIAEDFLIGNL

EPYQEALGYLGYKHHK

>GR37_CP039030

MKNDLVVKDNALINASYNLEVTEQRLILLSIIRARETGQGISSDSKLEIHASDYASRFDV

TKEAAYNALKNAVNNLFERKFSFKEIHKDTNKEIVVKSRWVSRIAYVDDLAILEVTFAPD

VVPLITRLEKHFTSYQLKQVAQLTSKYAIRLYEFLIAWRSTGKTPIISLSEFREKLGLDI

NEYQKMINFKNRVLEPAIKQINELTDIYVKYEQYKTGRSISGLSFTFKQKKAESLSQKKD

LNTLDLFTKMTDAQRHMFASKLSQLPEMSKYSQGTESYQQFSVRIAEMLQDPEQIKELYP

HLKKVGYIPSNNKDTVNG

>GR38_CP012007

MKKLSVSELAKLYGYSRQAIYAHINKGNLSKGSDGLIDFSEALRVFGEPQKKEDTVNQSQ

SINSQNLTEVDLLKRQVDILEKQLNQAIQRENQSLERESFYQEQIEAMQRLLEAPKANMT

TFTDQSFKQDIATDPRSELATNYDELTTPQQDNKRIPIPEHVEPEQKKRGFLSRFFLPYG

>GR39_CP034097

MPTSELIVKDNALINASYNLDTVEQRLILLAILQARETRTGIDANTRLRIHASDYMSRFN

VNKHAAYKALKTAVTNLFGRQFSYQTIDETGKSKKVISRWVQNISYIDDAATLEVTFTMD

VVPLITRLEKQFTSYQLKQVTQLTGKYAIRLYELLIAWREVGKTPIFEVSDFRSKLGLTS

DDYPRLDTFKRRVLESAVKQINEHTDIIVKVEQHKEGRSISGFSFSFKQKRNPNKAIELK

KDPNTLDIFSRITDAQRHLFANKLSKLPEMAKYSQGTESYQQFAIRIAEMLQDPAKFQEL

HPYLEKVGYR

>GR40_CP034094

MSDLIVKDNALISASYNLDVIEQRIILLSIIKARETGTGIDANTSLEIHASDYVKHFKVE

RQTAYEALKTAVNNLFNRQFSYTQPFQNTERVEYVKSRWVSRISYVDESAILNITFAPDI

VPLITKLEKHFTSYQLKRVAQLTSKYAIRLYELLIQWREVGKTPILEINDFRFKLGIEKH

EYKQMGHFKSRVLDPAIQQINEFTDIKTEYVQHKSGRVITGFEFKFALKDSGEEKTNNKN

ILNKEVVSMSDRQISLFANKLAYDEEFASRIAEIGESYEDLEKRLVKMLTKKENLVKWAK

DLQRIGFKAN

>GR41_CP051870

MQKIIGSQGLEDYTQSNTRAKLTDLVVTRNDFPTARYSIDLNLEKLMYCAMIIVRKNELK

NKTSITHDDFIYVSSENFGELTSPMARKEVLTATDKREIQRNAETALKRIYTKFDNPTML

VKDGESDEPAKVPMMTYCHYDKATKCIKVRFAKEFFEYFYDLVKKVDEKTKSFSSHELKH

IILFNSSYSLRLYRILMSYMWRTSEVTIDLEELRWMLECEDKYKELANFKNRVLNVAQDE

INELSNINVSFENVKNGKEVVAIKFIFSMKTEYKEQGHIKFIDKMKKGYLAAAIPFSDDG

SHFKAPDRIKHFKPPVKVSPKQISTLVNCKEFLNDYGYFLGNLDEDTSKVIMRTLLTEKL

DKLNAHKPIDMDYYFWLQAKRGIITNSNNDNKNDQDTDNQDTDDQD

>GR42_CM003909

MAKLSLSEVSKKFHVDRSTIYRAVRNGRLSRSSDGQFDLAEVIRCFGEPEQTSQKIESSK

QEGDESTKKLIAHLENEVKKYQEREERLMQQIDRMQTLIELKSVAPATAAPHQDATACDT

KMPQHATTQQDTDNKKNNELNIAENVAVPQQETTAYHTQTLQHATLQSVAVPQHKKRGLF

GRVLNAVFDND

>GR43_CP010400

MKSLTVLELSKLYNINRQTIYNNIKKGILSKNSDNKIDLAEAIRVFGEPSKKQDVKESVK

IDSPISTEVLLLRQQINMLQNQLDDAKERESFYQNQIETMQRLLEAPKPEIQEPSEPELP

QSAPIVEMKEQVIVVPPKDDGLTTPENKRIPVPEHVEPEPKKRGFWSRFFLPYG

>GR44_CM009044

MIDFIEMRLFVLDEFVISDRDGKHFLLSCDLLQLGVTVGSRDVYLDEQGNMQVGALYHPY

DDLPTSFTNVAFKLVHEGKIKPHVMIKCSPAKIMQGHNIFGSDNLELGVFEMLGFLAESH

PKLYKILDIPNAQIVNLDVTYSARLRNDDQVCKVLDFLRKVSSGSLRKSKLVYGSTVYWG

SPNSKRLCRKAYCKSIEFQLQLAKLKRQASKGEVFALRVIKAMEDPRVIEFMQGLLRLET

RFKPLWLTEHNIPLNVFDLIKYQSEHPNFLTDLWQLANKPLFEALEGHTMKALDHDTVFG

KICAKFDTYTKSGRLSQTKSRNIFNFFCALELHGSDELKKKYSKSQYYQYISDLMSCGFS

KAYLQNLDSESKNNVIPFVQLVKIDFQNQVPDWYQEPESRFAKVG

>GR45_CM004454

MAKLSLTEVSKRFKVSRSTVYRAIKEGKLSRSADNQFDLAEVIRCFGEPVIKASPEEQSK

APVKDDADLRQLVDFMKREIESYKEREQRYLDQIDRFQLLLGHKESSEKVSHDTPVTRSN

DTPCDTFNDTPEPIDTQAMIQNETSHDTPLTRLNDAPRDTFETSHDTLKKQPKKRGLLGR

VINAFFNDN

>GR46_CP051873

MLDKIVMHIPVDASLVDIDSEGRYCVFGFDLLDLGLRVGSYDVFKDEDGNVKHQVLNHAY

SKLPTSYTKMAFKFFHEGRTYPYVELKASPAKILQGHNVYGSDWIEQGALEMLGYLAESH

PTLYGMLAIGETEVKQLDATYSARLRDDNQVADAIDFLRNVTTQHIRKSTKQVTYKNTNY

YGSERCKRFARKVYGKSAEFQSQLEEQIKLAKANDKCAQRVVAVMSDPDLQQWVKGLLRF

ETGIKAYVMKELGIPTNLFELIRYQRSNPNFLRDIWIKANAQLFKALEGTAMKATDHDTV

FKNLCNVFGTVTPKGRQSFTKARNVFNFYCALEMNGYEVMKTRYGERQYYQYMADLIAAG

YSKAFLQNLHVESKSNIIPFLKLVEINFENQVPENFKEPISTFNQRELRIA

>GR47_CP008709

MYANTQNTQATAERGVPRTHIAPSVDGGSLGIYTDNSANRTTQGFQPIREKFKLLHFARK

LLPKERTAHCFYNRITKEEGVSVFLNKLRNKANYGNVMRCANPWACPVCSAIISEGRKDE

VKTAMDWWKAQGGDVLLLTLTAPHYSTTDIKSLKPAMLKARKYMLKGVRATKDLFKHYAI

EHYISVFEVTHGKNGFHPHFHILLFTKYHVHNPRGSVMRMQFFEQWKKACEKAGLDQPSY

EHGLDLRNGQKAASYVSKWGLEHEMTKGHIKKGKTDSKTPFDLLRDYAEGDENAGKLFRI

YFDAFKGTRQLNWSKGLKKLSSKGQEEKTDQELVDETDNVAELMFKLSIELWNPIRKHGR

QGELLIKVQEDHTLKKAMEYVKECLGYDGQLRE

>GR48_CP034096

MRTELVVKDNALINASYNLDLVEQRLILLAIVEARESGRGIDANKPLTVHASSYISQFNV

ARQTAYQALKDACKDLFARQFSYQEKREKGVANVTSRWVSQIAYIDDTASVELIFSPAII

PLITRLEEQFTSYELEQVSNLTSAYAVRLYELLIAWRSTGKTPVIELSEFRAKMGVLDGE

YTRSDNFKKWVIEKPIEQINEHTDITVKVEQHKTGRSITGFSFRFKQKQQPKIEKPIDPK

RDPNTPDFFVNMTDSQRYLFGHKLSTLQEMSEYSQGTESYEDFAKRIAEMLLDPEKFRTF

YPLLVQVGFK

>GR49_CM009050

MKKGKQQMTKVVKDNVLINASYSLDLVEQRVVLQGIVKSRETEKGFTDSNPVSIHASEYE

KQFGVTKDAAYKALKDAVLSLFERQFTFTELEKGKLKVVKSRWVSQIAYVDDLAEVQIIF

SPAVASMCSRLESHFTSYDLDQVSKLNSKYAVRLYELVIAWRATGKVPEIELSEFRNRLG

VCDNEYTAMNNFKKYVLDLAVSQINEHTDIKLDYEQHKKGRTISGFSFRLKSKVKQQKIE

QQRDPNTADLFTKMTDAQRHLFGNKLAHDARVQSDYSHLIGTGSYEDFGRLLADMLAEEK

HFKTFYPLLVECGFK

>GR50_CP042559

MSKNIVVKANILIEASYKLDLVEMRLILLAIIEARESKTLIDANSLIKISAKKYAEMFDI

SLKNSYSMLRGACKTLFDRQFSYVDWDFDLLSEDTSKIFFTSRWVSKVGYKPGFVYLSFA

PDVIPLISRLEKEFTRYDLLQISKFSSIYAVRIYEICMQWKIAKKFYISLSDLRNTLGLT

EIELSRIDNFQKKVLEVAKDQINESSDITIDYKTKKSGTKITGFEFTIRSKTSQPIKMEI

ALNDKQILLFSNKLVHEPSFASKYSKVGESYDDFAIRISENLTIPDKVQEYLPYLEKVGF

NTA

>GR51_CP050408

MKKDLVVKDNALINASYNLDLAEQRLILLAILEARESNIPSDRDLTIHAESYINHFNVHR

NTAYKVLKDACKNLFERRFSYQKLTAKGNLENVMSRWVQRVSYVENEALVRIRFSDDVVP

LITNLEKHFTSYELEQVSSLTSAYAIRLYELLIAWRSTGKVSMLETKELRSRLGVLDTEH

QRMESFKRRVLEPAIQQINDHTDIKAEYEQHKRGRSIIGFSFSFKQKSKPKTINHERDPS

TVDMFCNLSDSQINTYSSVLSKVHSISDLAGNKDYQAFAIWIANILRDPSSVREETAKRI

FKALRTETDFKG

>GR52_CP053216

MSNDLVVKDNVLINASYNLEVTEQRLILLSIITARETGQGITPESRLKIHASDYAERFNV

SKEASYDALKNAVNNLFERKFTFKEQLDNGKEIVVKSRWISQISYIPHLAILEIIFSPAV

VPLVTRLERHFTSYQLKQVAQLTSKYAIRLYELLIAWRSLGKTPILEISDFRQKIGVQDT

EYKTMSDFKKRVLEPAIEQINKETDITVKYDQHKTGRVVSGFEFKFKIKGEAASKKTTTQ

KKKVEELFCKISDKQVEIYSNKLSKQHELSDLAGNKDYAAFAIWIGNILRDKDSVRPETA

KRIFTALHTLTDFNK

>GR53_CP042564

MDLDVKKHYPKDWIVLQNRVVECFRGMSLDEKRLFIMATPLARTTKISSNDPIFISSSDF

SKECGIDLSTAYTALELASERLFTRFFGYTNAEGDRVKMRWLNKVIYKAGQGGSELYFTD

EVLLLLREFDALNPYTKYKKEVVLRLKKDYSLDFYHLAKKHQTMGGFQISLDELFQQLGL

PESYQDLSNLKKRVIKPSLDEITANTDIDLSYENVKRGRSVVGFKFTVKEKPKPKVIETG

RDPNTPDLFHKMTESQLDTFSSKLSELPEVQKMAHAGEDMKPFIARIRSMLKDSEKQKTL

LPHLAKLGFKSK

>GR54_CM009039

MNNAQLSDFFENLAHKPYCADDLLYGLQIRPKKTAINMQYIQGNQPCMLHYFFFDIDRSD

AVMAWHDENLPMPYWTAQTLKNGHAHICYKLEIPLCTSELASQKAIAYASKVQAGLANKL

GADVGYSHLITKNPFHKDWRVTFWSEQAYTLDYLADFVELPKKLSKKQEVSGLGRNCTMF

DTVRKWAYKAIRAHRGGIYTTWLDEVVKHCLSVNEAFLEPLPYSEVKATAKSIATYCWKK

DAYCYQEFIDRQSRKGAKGGTASNSSNGGKARAMKYTEIREKAISLRRDGKSIREIADSL

EVSKSAVANWVKQ

>GR55_CP042563

MNNAQLTDFFTNLAHKPYCADELLYGLKIRPKKTAINMQYIQGNQPCMLHYFFFDIDRSD

AVMAWHEQNLPVPYWTAQTQKNGHAHLCYKLEIPLCTSEFGSQKSIAYASKVQAALANRL

GADVGYSHLITKNPFHKDWRVTFWSEQAYTLDYLADFVELPKKLSKKQEVLGLGRNCTLF

ENARKWAYKAVRDYFHHHSSLEWDKAVLAHLEALNREFEVPLPYSEVKATAKSIAKYCWN

KFSYAGFSEWQSKNAERANAKGACSLGGKARSQQFNNLRQQALHLHIEGVNNTKIAEYLN

VSRKTITRWLNQVVIQ

>GR56_CP030107

MKDLIVKDNALINASYNLDLVEQRLILLSIIEARESGKGINANDPLTIHAESYINHFNVH

RNTAYQALKDACKDLFARQFSYQEQRGKGIANITSRWVSQIAYIDNTASVELIFAPAIIP

LITRLEEQFTSYELQQIKGLSTAYAIRLYELLIAWRSTGKTPIIETQELRRKLGVLDGEY

KMIADFKKRVFEPSIKQINEHTDITVKSEQHKTGRSITGFSFRFKQKAQPKLEQKTDPKR

DPNTPDFFVEMTDAQRHLFGNKLAHDARVQSEYSHLIGTGSYEDFAKLLADMLAEEQHFK

MFYPLLVEHGYKA

>GR57_AFDB02000005

MKSLSVIELSNLYGITRQAIYKQINKGNLSKNSDGKIDLAEAIRVFGEPSRNVNSSQTTE

TRKLSEVHLLEQQVYMLQKQLEQAHEREQFQREELKAKNDQLHVKDEQIEAIQRLLEAPK

AYITSSIDPKLDIATDTRSKSELNYDGLTTQPKEAPVEPKIQTQPKHDGLTTPELPENKR

IPVPDHVEPEQPKRGFWSRFFRPYD

>GR58_CP051879

MHGSQGFQRSNDFLLQDQSAKLLPKERVCNCLKKRIDKTKQREVKYNENRKKAHYANVQR

CGSIWSCPVCAKQITEKRRVELKKGLETWKNVHRGSVMLLTLTFSHSQSESLKSLLERQR

KAYKIFLETTKVKEIFKHFGVKYKIRSLEATYGQNGWHPHFHVLLLGYFKIEDLMYRDLL

AELWIKSCVRAGLNAPSMTHGLDLRDGTYADQYVSKWGIESELTKGHVKKGRNGGYTPFD

LLQFSMYNESVFEKDCGKLFQEFAIAMKGSRQLVWSRGLKALLELEEKTDEELAEETEKD

AISLRTIDDFIFSLLCHYQKRWDFLRCLERDYENGCFGTGETEQLLIDILEKEHIRLGIA

S

>GR59_CP090384

MTESDEQQELEYLPYCIGNIRHNGVVSTSNRLIRPIELSTNEYKALLYAMAVANYGEKNN

RDREITEQTYIYLHKDDLGELLGLNKKNSINVAIDRIYKELSSRVAHFVIEEPVDDLKRK

VKKVHSVVPIIRELRWEDDFKNALQIRFTSEVLPYFTRLANGNFTTYQLKDLFALDSVTS

MSLYSYIVRQEFKYANLDTYEVELSLESLKALIDIGEKKYDRWVDFRRYILDKIVEEINT

KTSLKLEYDSIKKGRPVVGVRFKIINGQEVNAISQSNEKTKIYLDVDFNDNNLVKELGAK

FDMTVRSWYIYANDPNCHQLERWFKPEDCLTDSQANVIVNDNLFQMEFAKPGLSMTEFKK

EMKNKLKNDRDFVQTNRNRLNEIFGKEII

>GR60_CP084302_this_study

MYGYKIVTMELGLKSTELIKPSSRRVQYTDLVATRNDMTTANYSYEANEEKLVYCAMVAV

RKNELNKNMRFDPDELITISAANFGELISEKHLDKDVVTASELYEIHRYGEKALQRVYDS

YKPKVMLIKKKDDPTPIKVPMIIYCHYSKETKCMQIRFAREFYNYFYNLINPTGMTHSFS

THQIRYVMRMRSNYAMRIYRILNSELWKAESLGVQQIHDISLERLRFALDIEDKYKLIDN

LKSRVLNVAKTQINKLSNLEVDYETIKNGKFVVGIRFTYKMKDEHRNLIFQRIIDRLKEK

HLKNAIPYRDDGSHFKDKERIKYIKPVTRLSSKQITVLVNCDVFLNDYGYFLGSLDTITA

KKMMRSLLINKLEILNDHKPIDLDYYFWIQAKRNMNIFKKDASEDNMADHEDKNIVDSDM

DEILDPSEDQLPFW

>GR61_CP048661_this_study

MSINGIYFFNHNMANLIYKDNNLIEASYALTLSEQRLILVAIIAAREIEKELTSDTLLTI

HASEYMKHFNLGRQAAYEALQGACDNLFERRLTYKAIDPVTGKPAVYKSRWVSKVGYVKE

AACAQLIFAPDILQLFVKLEEKFTRYELKQISQLSSVYAIRLYELLIRWRSKGKLYISMV

ELRDKLGLLENEYKTMGDFKKRVLTVAIDQINKLTDIDVSYEQKKEGRTITHIEFSFNQK

ASLIVSDKVLEPTYQLTPKQSIFFAQKLCDLIKYPEFGGKYANVGEEIEAFKERISLELL

DPEKVKKYYSDLLKVGYKEKYKSEKLS

>CP039146

MTESEPLQEESNYIPYCIGNIRHNGVVSTSNRLIRPIELSANEYKALLYAMAVANYSEKN

RVDGEITEQTYIYLYKDDLADLLGLNKRNSINVAIDRIYKELSSRVAHFIIEEPADNGKR

KTKKVHSVVPIIRELRWEDDSKNAIQIRFTSEVLPYFTQLAGGNFTTYQLKHLFALDSVA

SMSLYTYFIKNEFKYVNQKSYEVPLLLENLKALIDINETKYDRWVDFRRYVLDKIVAEIN

ENTDLQLEYETVKKGRPIVGVNFKLHHRIADKALDEFAVIEKIYLDVPFEDNASVKELGA

KFDMNVRSWYIFNNDENYQQFKKWFKKVGCLTDSQANIVINDTLFQMDFAEIGMGLNDFK

RNMKHKLKSNPEFVHSIRERLNDIFGKELI

>CP044520

MTESEPLQEESNYIPYCIGNIRHNGVVSTSNRLIRPIELSANEYKALLYAMAVANYSEKN

RVDGEITEQTYIYLYKDDLADLLGLNKRNSINVAIDRIYKELSSRVAHFIIEEPADNGKR

KTKKVHSVVPIIRELRWEDDSKNAIQIRFTSEVLPYFTQLAGGNFTTYQLKHLFALDSVA

SMSLYTYFIKNEFKYVNQKSYEVPLLLENLKALIDINETKYDRWVDFRRYVLDKIVAEIN

ENTDLQLEYETVKKGRPIVGVNFKLHHRIADKALDEFAVIEKIYLDVPFEDNASVKELGA

KFDMNVRSWYIFNNDENYQQFKKWFKKVGCLTDSQANIVINDTLFQMDFAEIGMGLNDFK

RNMKHKLKSNPEFVHSIRERLNDIFGKELI

>CP044457

MTESEPLQEESNYIPYCIGNIRHNGVVSTSNRLIRPIELSANEYKALLYAMAVANYSEKN

RVDGEITEQTYIYLYKDDLADLLGLNKRNSINVAIDRIYKELSSRVAHFIIEEPADNGKR

KTKKVHSVVPIIRELRWEDDSKNAIQIRFTSEVLPYFTQLAGGNFTTYQLKHLFALDSVA

SMSLYTYFIKNEFKYANQKSYEVPLLLENLKALIDINETKYDRWVDFRRYVLDKIVAEIN

ENTDLQLEYETVKKGRPIVGVNFKLHHRIADKALDEFAVIEKIYLDVPFEDNASVKELGA

KFDMNVRSWYIFNNDENYQQFKKWFKKVGCLTDSQANIVINDTLFQMDFAEIGMGLNDFK

RNMKHKLKSNPEFVHSIRERLNDIFGKELI

>CP044446

MTESEPLQEESNYIPYCIGNIRHNGVVSTSNRLIRPIELSANEYKALLYAMAVANYSEKN

RVDGEITEQTYIYLYKDDLADLLGLNKRNSINVAIDRIYKELSSRVAHFIIEEPADNGKR

KTKKVHSVVPIIRELRWEDDSKNAIQIRFTSEVLPYFTQLAGGNFTTYQLKHLFALDSVA

SMSLYTYFIKNEFKYANQKSYEVPLLLENLKALIDINETKYDRWVDFRRYVLDKIVAEIN

ENTDLQLEYETVKKGRPIVGVNFKLHHRIADKALDEFAVIEKIYLDVPFEDNASVKELGA

KFDMNVRSWYIFNNDENYQQFKKWFKKVGCLTDSQANIVINDTLFQMDFAEIGMGLNDFK

RNMKHKLKSNPEFVHSIRERLNDIFGKELI

>CP040912

MTESEPLQEESNYIPYCIGNIRHNGVVSTSNRLIRPIELSANEYKALLYAMAVANYSEKN

RVDGEITEQTYIYLYKDDLADLLGLNKRNSINVAIDRIYKELSSRVAHFIIEEPADNGKR

KTKKVHSVVPIIRELRWEDDSKNAIQIRFTSEVLPYFTQLAGGNFTTYQLKHLFALDSVA

SMSLYTYFIKNEFKYVNQKSYEVPLLLENLKALIDINETKYDRWVDFRRYVLDKIVAEIN

ENTDLQLEYETVKKGRPIVGVNFKLHHRIADKALDEFAVIEKIYLDVPFEDNASVKELGA

KFDMNVRSWYIFNNDENYQQFKKWFKKVGCLTDSQANIVINDTLFQMDFAEIGMGLNDFK

RNMKHKLKSNPEFVHSIRERLNDIFGKELI

>CP044019

MTESEPLQEESNYIPYCIGNIRHNGVVSTSNRLIRPIELSANEYKALLYAMAVANYSEKN

RVDGEITEQTYIYLYKDDLADLLGLNKRNSINVAIDRIYKELSSRVAHFIIEEPADNGKR

KTKKVHSVVPIIRELRWEDDSKNAIQIRFTSEVLPYFTQLAGGNFTTYQLKHLFALDSVA

SMSLYTYFIKNEFKYANQKSYEVPLLLENLKALIDINETKYDRWVDFRRYVLDKIVAEIN

ENTDLQLEYETVKKGRPIVGVNFKLHHRIADKALDEFAVIEKIYLDVPFEDNAYVKELGA

KFDMNVRSWYIFNNDENYQQFKKWFKKVGCLTDSQANIVINDTLFQMDFAEIGMGLNEFK

RNMKHKLKSNPEFVHSIRERLNDIFGKELI

>CP045132

MTESEPLQEESNYIPYCIGNIRHNGVVSTSNRLIRPIELSANEYKALLYAMAVANYSEKN

RVDGEITEQTYIYLYKDDLADLLGLNKRNSINVAIDRIYKELSSRVAHFIIEEPADNGKR

KTKKVHSVVPIIRELRWEDDSKNAIQIRFTSEVLPYFTQLAGGNFTTYQLKHLFALDSVA

SMSLYTYFIKNEFKYVNQKSYEVPLLLENLKALIDINETKYDRWVDFRRYVLDKIVAEIN

ENTDLQLEYETVKKGRPIVGVNFKLHHRIADKALDEFAVIEKIYLDVPFEDNASVKELGA

KFDMNVRSWYIFNNDENYQQFKKWFKKVGCLTDSQANIVINDTLFQMDFAEIGMGLNDFK

RNMKHKLKSNPEFVHSIRERLNDIFGKELI

>CP048015

MTESESLQEELNHIPYCIGNIRHNGVVSTSNRLIRPIELSANEYKALLYAMAVANYSEKN

RVNGEITEQTYIYLYKDDLADLLGLNKRNSINVAIDRIYKELSSRVAHFIIEEPTDDGKR

KTKKVHSVVPIIRELRWEDDSKNAIQIRFTSEVLPYFTQLAGGNFTTYQLKHLFALDSVA

SMSLYTYFIKNEFKYASQKSYEVPMLLENLKALIDINETKYDRWVDFRRYVLDKIVAEIN

ENTDLQLEYETIKKGRPIVGVNFKLHQRITDKTLNETAVIEKIYLDVPFEENALVKELGA

KFDTNVRSWYIFNNDEKYSQFKKWFKKIGCLTDSQANIVINDTLFQMDFAEVGMSLNDFK

RNMKHKLKNNPEFVQSIRDRLNDIFGKELV

>CP071767

MTESESLQEELNHIPYCIGNIRHNGVVSTSNRLIRPIELSANEYKALLYAMAVANYSEKN

RVNGEITEQTYIYLYKDDLADLLGLNKRNSINVAIDRIYKELSSRVAHFIIEEPTDDGKR

KTKKVHSVVPIIRELRWEDDSKNAIQIRFTSEVLPYFTQLAGGNFTTYQLKHLFALDSVA

SMSLYTYFIKNEFKYASQKSYEVPMLLENLKALIDINETKYDRWVDFRRYVLDKIVAEIN

ENTDLQLEYETIKKGRPIVGVNFKLHQRITDKTLNETAVIEKIYLDVPFEENALVKELGA

KFDTNVRSWYIFNNDEKYSQFKKWFKKIGCLTDSQANIVINDTLFQMDFAEVGMSLNDFK

RNMKHKLKNNPEFVQSIRDRLNDIFGKELV

>CP071769

MTESESLQEELNHIPYCIGNIRHNGVVSTSNRLIRPIELSANEYKALLYAMAVANYSEKN

RVNGEITEQTYIYLYKDDLADLLGLNKRNSINVAIDRIYKELSSRVAHFIIEEPTDDGKR

KTKKVHSVVPIIRELRWEDDSKNAIQIRFTSEVLPYFTQLAGGNFTTYQLKHLFALDSVA

SMSLYTYFIKNEFKYASQKSYEVPMLLENLKALIDINETKYDRWVDFRRYVLDKIVAEIN

ENTDLQLEYETIKKGRPIVGVNFKLHQRITDKTLNETAVIEKIYLDVPFEENALVKELGA

KFDTNVRSWYIFNNDEKYSQFKKWFKKIGCLTDSQANIVINDTLFQMDFAEVGMSLNDFK

RNMKHKLKNNPEFVQSIRDRLNDIFGKELV

>CP071772

MTESESLQEELNHIPYCIGNIRHNGVVSTSNRLIRPIELSANEYKALLYAMAVANYSEKN

RVNGEITEQTYIYLYKDDLADLLGLNKRNSINVAIDRIYKELSSRVAHFIIEEPTDDGKR

KTKKVHSVVPIIRELRWEDDSKNAIQIRFTSEVLPYFTQLAGGNFTTYQLKHLFALDSVA

SMSLYTYFIKNEFKYASQKSYEVPMLLENLKALIDINETKYDRWVDFRRYVLDKIVAEIN

ENTDLQLEYETIKKGRPIVGVNFKLHQRITDKTLNETAVIEKIYLDVPFEENALVKELGA

KFDTNVRSWYIFNNDEKYSQFKKWFKKIGCLTDSQANIVINDTLFQMDFAEVGMSLNDFK

RNMKHKLKNNPEFVQSIRDRLNDIFGKELV

>CP046596

MTESEPLQEESNYIPYCIGNIRHNGVVSTSNRLIRPIELSANEYKALLYAMAVANYSEKN

RVDGEITEQTYIYLYKDDLADLLGLNKRNSINVAIDRIYKELSSRVAHFIIEEPADNGKR

KTKKVHSVVPIIRELRWEDDSKNAIQIRFTSEVLPYFTQLAGGNFTTYQLKHLFALDSVA

SMSLYTYFIKNEFKYANQKSYEVPLLLENLKALIDINETKYDRWVDFRRYVLDKIVAEIN

ENTDLQLEYETVKKGRPIVGVNFKLHHRIADKALDEFAVIEKIYLDVPFEDNASVKELGA

KFDMNVRSWYIFNNDENYQQFKKWFKKVGCLTDSQANIVINDTLFQMDFAEIGMGLNDFK

RNMKHKLKSNPEFVHSIRERLNDIFW

>CP094546

MTESEPLQEESNYIPYCIGNIRHNGVVSTSNRLIRPIELSANEYKALLYAMAVANYSEKN

RVDGEITEQTYIYLYKDDLADLLGLNKRNSINVAIDRIYKELSSRVAHFIIEEPADNGKR

KTKKVHSVVPIIRELRWEDDSKNAIQIRFTSEVLPYFTQLAGGNFTTYQLKHLFALDSVA

SMSLYTYFIKNEFKYANQKSYEVPLLLENLKALIDINETKYDRWVDFRRYVLDKIVAEIN

ENTDLQLEYETVKKGRPIVGVNFKLHHRIADKALDEFAVIEKIYLDVPFEDNASVKELGA

KFDMNVRSWYIFNNDENYQQFKKWFKKVGCLTDSQANIVINDTLFQMDFAEIGMGLNDFK

RNMKHKLKSNPEFVHSIRERLNDIFGKELI

>CP044464

MTESEPLQEESNYIPYCIGNIRHNGVVSTSNRLIRPIELSANEYKALLYAMAVANYSEKN

RVDGEITEQTYIYLYKDDLADLLGLNKRNSINVAIDRIYKELSSRVAHFIIEEPADNGKR

KTKKVHSVVPIIRELRWEDDSKNAIQIRFTSEVLPYFTQLAGGNFTTYQLKHLFALDSVA

SMSLYTYFIKNEFKYANQKSYEVPLLLENLKALIDINETKYDRWVDFRRYVLDKIVAEIN

ENTDLQLEYETVKKGRPIVGVNFKLHHRIADKALDEFAVIEKIYLDVPFEDNASVKELGA

KFDMNVRSWYIFNNDENYQQFKKWFKKVGCLTDSQANIVINDTLFQMDFAEIGMGLNDFK

RNMKHKLKSNPEFVHSIRERLNDIFGKELI

>CP044484

MTESEPLQEESNYIPYCIGNIRHNGVVSTSNRLIRPIELSANEYKALLYAMAVANYSEKN

RVDGEITEQTYIYLYKDDLADLLGLNKRNSINVAIDRIYKELSSRVAHFIIEEPADNGKR

KTKKVHSVVPIIRELRWEDDSKNAIQIRFTSEVLPYFTQLAGGNFTTYQLKHLFALDSVA

SMSLYTYFIKNEFKYANQKSYEVPLLLENLKALIDINETKYDRWVDFRRYVLDKIVAEIN

ENTDLQLEYETVKKGRPIVGVNFKLHHRIADKALDEFAVIEKIYLDVPFEDNASVKELGA

KFDMNVRSWYIFNNDENYQQFKKWFKKVGCLTDSQANIVINDTLFQMDFAEIGMGLNDFK

RNMKHKLKSNPEFVHSIRERLNDIFGKELI

>CP044475

MTESEPLQEESNYIPYCIGNIRHNGVVSTSNRLIRPIELSANEYKALLYAMAVANYSEKN

RVDGEITEQTYIYLYKDDLADLLGLNKRNSINVAIDRIYKELSSRVAHFIIEEPADNGKR

KTKKVHSVVPIIRELRWEDDSKNAIQIRFTSEVLPYFTQLAGGNFTTYQLKHLFALDSVA

SMSLYTYFIKNEFKYANQKSYEVPLLLENLKALIDINETKYDRWVDFRRYVLDKIVAEIN

ENTDLQLEYETVKKGRPIVGVNFKLHHRIADKALDEFAVIEKIYLDVPFEDNASVKELGA

KFDMNVRSWYIFNNDENYQQFKKWFKKVGCLTDSQANIVINDTLFQMDFAEIGMGLNDFK

RNMKHKLKSNPEFVHSIRERLNDIFGKELI

>CP094542

MTESEPLQEESNYIPYCIGNIRHNGVVSTSNRLIRPIELSANEYKALLYAMAVANYSEKN

RVDGEITEQTYIYLYKDDLADLLGLNKRNSINVAIDRIYKELSSRVAHFIIEEPADNGKR

KTKKVHSVVPIIRELRWEDDSKNAIQIRFTSEVLPYFTQLAGGNFTTYQLKHLFALDSVA

SMSLYTYFIKNEFKYANQKSYEVPLLLENLKALIDINETKYDRWVDFRRYVLDKIVAEIN

ENTDLQLEYETVKKGRPIVGVNFKLHHRIADKALDEFAVIEKIYLDVPFEDNASVKELGA

KFDMNVRSWYIFNNDENYQQFKKWFKKVGCLTDSQANIVINDTLFQMDFAEIGMGLNDFK

RNMKHKLKSNPEFVHSIRERLNDIFGKELI

>CP044451

MTESEPLQEESNYIPYCIGNIRHNGVVSTSNRLIRPIELSANEYKALLYAMAVANYSEKN

RVDGEITEQTYIYLYKDDLADLLGLNKRNSINVAIDRIYKELSSRVAHFIIEEPADNGKR

KTKKVHSVVPIIRELRWEDDSKNAIQIRFTSEVLPYFTQLAGGNFTTYQLKHLFALDSVA

SMSLYTYFIKNEFKYANQKSYEVPLLLENLKALIDINETKYDRWVDFRRYVLDKIVAEIN

ENTDLQLEYETVKKGRPIVGVNFKLHHRIADKALDEFAVIEKIYLDVPFEDNASVKELGA

KFDMNVRSWYIFNNDENYQQFKKWFKKVGCLTDSQANIVINDTLFQMDFAEIGMGLNDFK

RNMKHKLKSNPEFVHSIRERLNDIFGKELI

>MT107270

MTESEPLQEESNYIPYCIGNIRHNGVVSTSNRLIRPIELSANEYKALLYAMAVANYSEKN

RVDGEITEQTYIYLYKDDLADLLGLNKRNSINVAIDRIYKELSSRVAHFIIEEPADNGKR

KTKKVHSVVPIIRELRWEDDSKNAIQIRFTSEVLPYFTQLAGGNFTTYQLKHLFALDSVA

SMSLYTYFIKNEFKYANQKSYEVPLLLENLKALIDINETKYDRWVDFRRYVLDKIVAEIN

ENTDLQLEYETVKKGRPIVGVNFKLHHRIADKALDEFAVIEKIYLDVPFEDNASVKELGA

KFDMNVRSWYIFNNDENYQQFKKWFKKVGCLTDSQANIVINDTYSKWILLKLVWG

>CP094556

MTESEPLQEESNYIPYCIGNIRHNGVVSTSNRLIRPIELSANEYKALLYAMAVANYSEKN

RVDGEITEQTYIYLYKDDLADLLGLNKRNSINVAIDRIYKELSSRVAHFIIEEPADNGKR

KTKKVHSVVPIIRELRWEDDSKNAIQIRFTSEVLPYFTQLAGGNFTTYQLKHLFALDSVA

SMSLYTYFIKNEFKYANQKSYEVPLLLENLKALIDINETKYDRWVDFRRYVLDKIVAEIN

ENTDLQLEYETVKKGRPIVGVNFKLHHRIADKALDEFAVIEKIYLDVPFEDNASVKELGA

KFDMNVRSWYIFNNDENYQQFKKWFKKVGCLTDSQANIVINDTLFQMDFAEIGMGLNDFK

RNMKHKLKSNPEFVHSIRERLNDIFGKELI

>CP041290

MQKIIGSQGLEDYTQSNTRAKLTDLVVTRNDFPTARYSIDLNLEKLMYCAMIIVRKNELK

NKTLITHDDFIYVSSENFGELTSPMARKEVLTATDKREIQRNAETALKRIYTKFDNPTML

VKDGESDEPAKVPMMTYCHYDKATKCIKVRFAKEFFEYFYDLVKKVDEKTKSFSSHELKH

IILFNSSYSLRLYRILMSYMWRTSEVTIDLEELRWMLECEDKYKELANFKNRVLNVAQDE

INELSNINVSFENVKNGKEVVAIKFIFSLKTEYKEQGHIKFIDKMKKGYLAAAIPFSDDG

SHFKAPDRIKHFKPPVKVSPKQISTLVNCKEFLNDYGYFLGNLDEDTSKVIMRTLLTEKL

DKLNAHKPIDMDYYFWLQAKRGIITNSNNDKKNDQDTDNQDTDNQD

>CP041297

MTDTDHTEEQEYLPYCIGNIRQNGVVSTSNRLIRPIELSTNEYKALLYAMAVANYGEKNN

QDREITEQTYIYLHKDDLGELLGLNKKNSINVAIDRIYKELSSRVAHFVIEEPADDPKRK

VKKVHSVVPIIRELRWEDDLKNALQIRFTSEVLPYFTRLANGNFTTYQLKDLFALDSVTS

MSLYSYIVKQEFKYANQDTYEVELSLEGLKALIDIGETKYDRWVDFRRYILDRIVAEINS

KTSLKLEYDTIKKGRPIVGVRFKILNDKKENAISQSKEKTKIYLDVDFNDNNLVKELGAK

FDMTVRSWYIYANDPNSQKLKKWFKPEGCLTDSQANVIVNDNLFQMEFAKPGLSMAEFKK

EMKHKLKNDRDFVQANRTRLNEIFGKEII

>MK134375

YLALIEAEDSLFKRQFTITNEDGTLTKSRWIQDANYRKGEGRILVTLTRVVIEHVTKIDG

FEQYFTSYHLKKTADFKSVYAVRLYELLMQWKSVGKTPVYELNKFRSQLGIGVNEYTRME

AFKRRVLDIAVDQINEFSDITVKYEQHKKGRSISGFSFSFKPKKATIRSIETNRDPNTTD

LFSRMTDKQRHLFATKLSELPEMGKYSQGTESYPQFAIRIAEMLQDYQKFQELFPYLQKV

GYQAA

>CP090315

MTESEPLQEESNYIPYCIGNIRHNGVVSTSNRLIRPIELSANEYKALLYAMAVANYSEKN

RVDGEITEQTYIYLYKDDLADLLGLNKRNSINVAIDRIYKELSSRVAHFIIEEPADNGKR

KTKKVHSVVPIIRELRWEDDSKNAIQIRFTSEVLPYFTQLAGGNFTTYQLKHLFALDSVA

SMSLYTYFIKNEFKYANQKSYEVPLLLENLKALIDINETKYDRWVDFRRYVLDKIVAEIN

ENTDLQLEYETVKKGRPIVGVNFKLHHRIADKALDEFAVIEKIYLDVPFEDNASVKELGA

KFDMNVRSWYIFNNDENYQQFKKWFKKVGCLTDSQANIVINDTLFQMDFAEIGMGLNDFK

RNMKHKLKSNPEFVHSIRERLNDIFGKELI

>CP090068

MSELSHNKEELDSIPYCIGNIRHNGVVSTSNRLIRPIELSANEYKALLYAMAVANYGEKN

HIDKEISEQTYIYLYKDDLAELLGLSKRNSINVAIDRIYKELSSRVAHFVIEEPVDDIKK

KTKRVHSVVPIIRELRWEDDSKNALQIRFTSEVLPYFTQLAGGNFTTYQLKHLFALDSVA

SMSLYTYFIKNEFKFKTQEIYEVPLLLENLKALIDINETKYDRWVDFRRYVLDKIVSEIN

ENTDLQLEYETIKKGRPIVGVKFKLHHRGFEKPVIENKKAEKLKIYLDVPFEDNAEVKDL

GAKFDTTVRSWYIFNDDANYLQLEKWFKTVGCLTDSQANVVINDSLFQMDFAEVGMSLSD

FKRKMKNKLKTDSKFVEDIKDRLNEIFGKDII

>CP084298

MTESESLQEELNHIPYCIGNIRHNGVVSTSNRLIRPIELSANEYKALLYAMAVANYSEKN

RVNGEITEQTYIYLYKDDLADLLGLNKRNSINVAIDRIYKELSSRVAHFIIEEPTDDGKR

KTKKVHSVVPIIRELRWEDDSKNAIQIRFTSEVLPYFTQLAGGNFTTYQLKHLFALDSVA

SMSLYTYFIKNEFKYASQKSYEVPMLLENLKALIDINETKYDRWVDFRRYVLDKIVAEIN

ENTDLQLEYETIKKGRPIVGVNFKLHQRITDKTLNETAVIEKIYLDVPFEENALVKELGA

KFDTNVRSWYIFNNDEKYSQFKKWFKKIGCLTDSQANIVINDTLFQMDFAEVGMSLNDFK

RNMKHKLKNNPEFVQSIRDRLNDIFGKELV
