# Supplementary figures and images for: Classification and molecular characteristics of tet(X)-carrying plasmids in Acinetobacter species
Source: Front Microbiol. 2022 Aug 23;13:974432. doi: 10.3389/fmicb.2022.974432 (PMC9445619; doi:10.3389/fmicb.2022.974432)

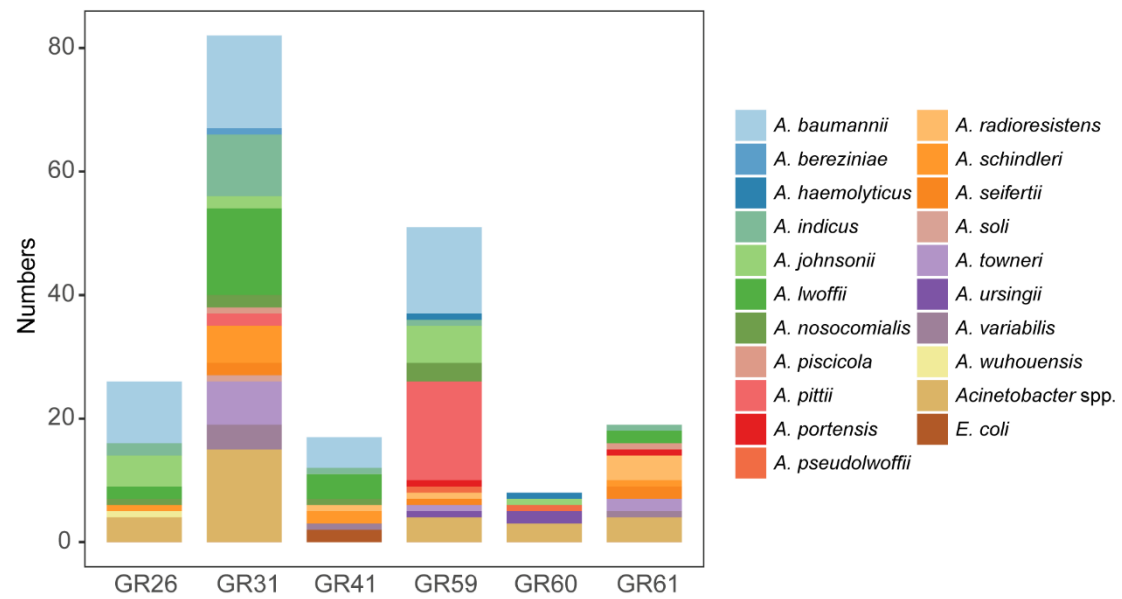

**Supplementary Figure 1** | Distribution of GR26, GR31, GR41, GR59, GR60, and GR61 plasmids.

Supplement: Supplementary file 4 [file Image_1.pdf]
